# Achondroplasia: a comprehensive clinical review

**DOI:** 10.1186/s13023-018-0972-6

**Published:** 2019-01-03

**Authors:** Richard M. Pauli

**Affiliations:** Midwest Regional Bone Dysplasia Clinic, Department of Pediatrics, University of Wisconsin School of Medicine and Public Health, 1500 Highland Ave., Madison, WI 53705 USA

**Keywords:** Achondroplasia, FGFR3, Skeletal dysplasia, Natural history, Care guidelines

## Abstract

Achondroplasia is the most common of the skeletal dysplasias that result in marked short stature (dwarfism). Although its clinical and radiologic phenotype has been described for more than 50 years, there is still a great deal to be learned about the medical issues that arise secondary to this diagnosis, the manner in which these are best diagnosed and addressed, and whether preventive strategies can ameliorate the problems that can compromise the health and well being of affected individuals. This review provides both an updated discussion of the care needs of those with achondroplasia and an exploration of the limits of evidence that is available regarding care recommendations, controversies that are currently present, and the many areas of ignorance that remain.

## Introduction

Explicit guidelines for care of individuals with achondroplasia are available. Such guidelines were first developed by the American Academy of Pediatrics in 1995 and revised in 2005 [1]. These are now again somewhat out of date. Other care guidelines (for example see [2–4]) and clinically oriented reviews (such as [5–7]) are also available. However, none of these explores in detail the bases for recommendations and the uncertainties that exist. Therefore, this review is intended as both an updated discussion of care needs in achondroplasia and a platform for exploration of the evidence for recommendations, current controversies and areas of current ignorance (which are many).

As is the case for virtually all uncommon or rare genetic disorders, the level of evidence for care recommendations in achondroplasia is generally low. No controlled or blinded studies of any sort are available. Very few prospective investigations have been published (such as [8, 9] and a few others). Most care suggestions are based on retrospective series of varying size, or anecdotal information that lacks any rigorous confirmation. Both retrospective studies of large populations and selective prospective studies are much needed. Nonetheless, something has to be recommended for the care of affected individuals. Not surprisingly, lack of rigorous studies also results in considerable variation in the recommendations that are made. Unfortunately, this is not terribly different from much of current medical care. Some of these uncertainties will yield to studies of larger populations, as have been initiated recently [10].

## History

The achondroplasia phenotype has been recognized for thousands of years, as evidenced in the artifacts of many different cultures [11], and remains the most readily recognizable of the dwarfing disorders. The term seems to have been first used in the nineteenth century, and, while the main features were described shortly thereafter [6], it often was used as a generic descriptor of all short-limb dwarfing disorders (in contrast to the short-trunk or Morquio type) for the first half of the twentieth century. Detailed and specific radiologic and clinical features were carefully delineated by Langer et al. [12]. It remains the best characterized and most studied of the hundreds of dwarfing skeletal dysplasias. It is sufficiently common that many pediatricians and family practitioners will help care for one or more individuals in their practices.

Appropriate distinction between this and other short-limb dwarfing disorders was, and remains, crucial, of course. Earlier confusion with thanatophoric dysplasia led to the erroneous conclusion that adults with achondroplasia had risk to have children with a lethal form of achondroplasia; conflating of achondroplasia and recessive short-limb dwarfing processes suggested, incorrectly, that parents of average stature with one child with achondroplasia might have high risk for recurrence.

## Genetics

### Prevalence

Birth prevalence has been estimated in a number of populations [13] (also [14–16]). These studies yield fairly consistent estimates whether these are population based or hospital based assessments. Together they suggest that achondroplasia arises in about 1 in every 25,000–30,000 individuals. That, in turn, translates into around 250,000 affected persons worldwide [3].

### Formal genetics

All instances of achondroplasia arise from mutations that are autosomal dominant. These mutations are fully penetrant and show only modest variability of expression. Because of its dominant inheritance pattern, an individual affected with achondroplasia (and whose partner is of average stature) has a 50% risk for each of their offspring to be similarly affected. However, most instances of achondroplasia – perhaps 80% – arise from new, spontaneous mutations [17]. In turn, then, around 80% of affected babies are born to two unaffected, average statured parents.

One would anticipate that recurrences to average statured parents should be no greater than occurrence in the population as a whole. That does not seem to be the case, however. Quite a number of unexpected instances of recurrence in siblings has been observed [18–22] (and personal observations). While likely increased, almost certainly that recurrence risk is far less than 1% [19]. The likely molecular explanation for this increased risk is discussed below.

### Special considerations regarding recurrence risks

#### Homozygosity and compound heterozygosity

Assortative mating – that is, greater probability of partnering with an individual with a phenotype similar to one’s own – is particularly common within the community of individuals with dwarfing disorders [23]. Because achondroplasia is much more common than any other dwarfing dysplasia, the most common of such matings is between two individuals both of whom have achondroplasia. As for many other so-called dominant processes, pure dominance (having one abnormal allele or having two such alleles resulting in indistinguishable phenotypes) is not observed. Rather, a ‘double dose’ of the achondroplasia-causing mutation results in a far more severe process [24]. Indeed, homozygous achondroplasia is virtually always lethal in the newborn period [24]. These early deaths probably arise through mechanisms that also place babies with heterozygous achondroplasia at risk – restrictive pulmonary disease and craniocervical junction constriction [24, 25], as discussed below.

Risk for homozygous achondroplasia when both parents have achondroplasia is 25% (as well as there being a 50% chance for heterozygous achondroplasia and 25% chance for a child with average stature). Many at risk couples opt for preimplantation diagnosis or prenatal diagnosis solely to rule out homozygosity, while others may elect adoption (often of small statured individuals).

Hypochondroplasia is a generally somewhat less severe small stature disorder that often is caused by mutation in the same gene as the mutations that result in achondroplasia. If one parent has achondroplasia and one has hypochondroplasia, then there is a 25% risk for a child with *compound heterozygosity* for both achondroplasia and hypochondroplasia. This results in a very severe phenotype that includes cognitive disability and substantial medical problems [26–29].

#### Double heterozygosity

Similarly the result of the aforementioned assortative mating, *double heterozygosity* may arise when two parents have two *different* and non-allelic bone dysplasias [30, 31]. Each of these is rare. Each has a distinctive phenotype. Some result in a very poor prognosis (e.g. achondroplasia-SEDC [30, 32, 33], while others may have quite variable outcome (e.g. achondroplasia-pseudoachondroplasia [34] [and personal observation]). Others may actually result in an ameliorating effect [35]. The possible outcomes are sufficiently complex that formal counseling should be recommended in all such instances.

#### Coincidental co-occurrences

It shouldn’t come as a surprise that relatively common disorders may co-occur within the same individual. A number of such coincidental co-occurrences have been described in individuals with achondroplasia. Of particular note is the occurrence of achondroplasia plus Down syndrome. It should be expected that this arises on occasion: Down syndrome is more frequent in the offspring of older mothers, while achondroplasia is more common in children of older fathers; and, of course, maternal and paternal ages tend to co-vary. Seven instances have been reported in the literature [36, 37] but there are certainly many more that have not been reported (including three personal observations). Unfortunately, these two disorders have features that, together, can result in very severe problems – hypotonia in both; craniocervical junction issues in both; restrictive pulmonary disease in both. Not surprisingly, then, this combination often results in death in infancy [36].

### Molecular genetics and molecular pathogenesis

#### Discovery of the molecular cause of achondroplasia

Thousands of years after its recognition, nearly a century after its clinical description, and a quarter century after it clear clinical and radiologic delineation, the molecular basis of achondroplasia was discovered. Shiang et al. [38] showed that individuals with achondroplasia have identifiable mutations in the fibroblast growth factor receptor type 3 (*FGFR3*) gene. Rapidly it was demonstrated that nearly all instances of achondroplasia are caused by *FGFR3* mutations [39, 40]. This locus homogeneity was not particularly surprising. What was unexpected is that virtually all mutations in *FGFR3* arise in the same nucleotide pair and result in the same glycine to arginine substitution (G380R) in the FGFR3 protein [40]. This specific mutation is at least 500- or 1000-fold more frequent than expected [41, 42].

#### Features of FGFR3

**Fig. 1:**
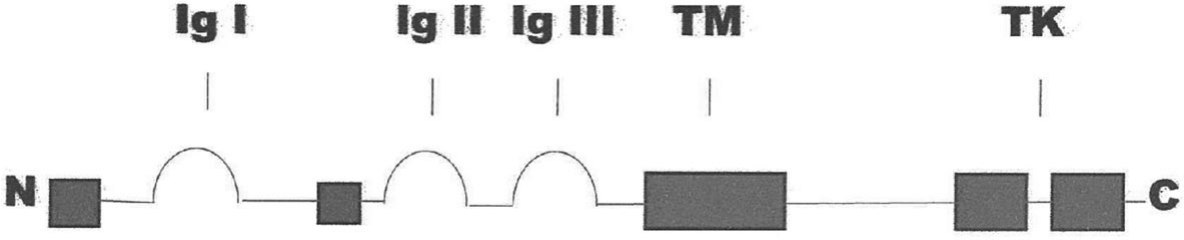
Simplified diagram of the FGFR3 protein, including three immunoglobulin-like domains (Ig), a transmembrane domain (TM), and the split tyrosine kinase (TK) region

FGFR3 is one of four fibroblast growth factor receptors in humans. All are cell surface receptors that influence cellular proliferation. FGFR3 is comprised of an extracellular domain with three immunoglobulin-like regions, a transmembrane domain and an intracellular tyrosine kinase [43] (Fig. 1). It can be pictured as an empty cup sitting on the surface of cells. It is particularly prevalent on the surface of chondrocytes that give rise to cartilaginous bone [44], but is also expressed in calvarial sutures [45], testes [46], and the brain [47].

Under “normal” conditions the typical FGFR3 is silent. However, various fibroblast growth factors (FGFs) – principally FGFs 2, 9, 18 and 23 [48] – can act as ligands, binding to the FGFR3 [44, 48], in effect filling the cup. This results in dimerization of the receptors, transphosphorylation and trans-activation of tyrosine kinases, and propagation of an intracellular signal [43]. Although downstream signaling is complex [48, 49], overall the signal within the growth plate of cartilaginous bones is negative. That is, overall FGFR3 is a negative regulator of chondrocytic bone growth (through shortening of the proliferative phase and accelerating terminal differentiation [49]). The “full cup”, then, results in a net “slow down” signal inside relevant cells.

#### FGFR3 in achondroplasia

The mutation that results in achondroplasia is a gain of function mutation [50] rather than an inactivating mutation. It most likely results in ligand *independent* activation of FGFR3 [50, 51]. This, then, is constitutive activation of an inhibitory signal. Or, one can think of this as a continuous “slow down” signal, released from the usual ligand-based constraints.

Dysplasias can be sorted into families in which members differ mostly by severity [52]. Other disorders within the achondroplasia family (and discussed below) are also caused by different mutations in *FGFR3*. Severity seems to be a consequence of a graded series of relative activation of FGFR3 [53–55].

Virtually all of the clinical features and medical problems of achondroplasia arise because of the consequent abnormalities of cartilaginous bone growth – either directly, or because of disproportionate growth of cartilaginous bone compared with nearby structures derived from other tissues.

#### Origin of the unexpected frequency of the achondroplasia mutation

Why is the mutation resulting in the G380R amino acid substitution so frequent? This is related to the paternal age effect which has already been briefly mentioned. It has been recognized for a long time that certain genetic disorders arising through new mutations occur far more frequently in the offspring of older fathers [56]. That phenomenon is particularly marked in achondroplasia [17]. Both the origin of this paternal age effect and the exceedingly high apparent mutation rate have a single basis [41, 42]. That basis also helps explain why all mutations in sporadic cases of achondroplasia are paternal in origin [57].

It seems that certain mutant protein products, including of *FGFR3*, are positively selected for in sperm precursor cells (spermatogonial stem cells). Once such a mutation occurs there will be clonal expansion of cells containing the mutation and consequent enrichment within the spermatogonial population. This positive selection within germinal precursors, rather than an actual increased mutation rate, probably explains the prevalence of achondroplasia. If, as seems to be the case, such selection only occurs in male germinal precursors, this also explains the paternal origin of virtually all instances of achondroplasia. Furthermore, since clonal expansion will cause more and more enrichment with time [58], fertilization involving a sperm with such a mutation becomes more likely with advancing paternal age. Finally, this also explains why some men have more than one “sporadically” affected child [22].

Achondroplasia is one of a small number of so-called RAMP disorders – recurrent, autosomal dominant, male biased, paternal age effect disorders – all of which likely arise because of their positive selective effect on spermatogonia. Other disorders for which there is convincing evidence of similar effects include Apert syndrome, Noonan syndrome, and multiple endocrine neoplasia type 2B [59].

## Presentation and diagnosis

### Diagnosis in the neonate

The vast majority of individuals with achondroplasia are diagnosed in early infancy, although prenatal recognition has become more frequent and more accurate. It is critical that diagnosis not be delayed since certain complications can only be prevented through assessment in early infancy (see Special Concerns in the Young Infant).

No formal clinical diagnostic criteria have been published, but well defined clinical and radiologic characteristics of achondroplasia [12] usually allow for virtual certainty. In certain circumstances, particularly in the markedly premature neonate [60], clinical diagnosis may be especially challenging.

**Fig. 2:**
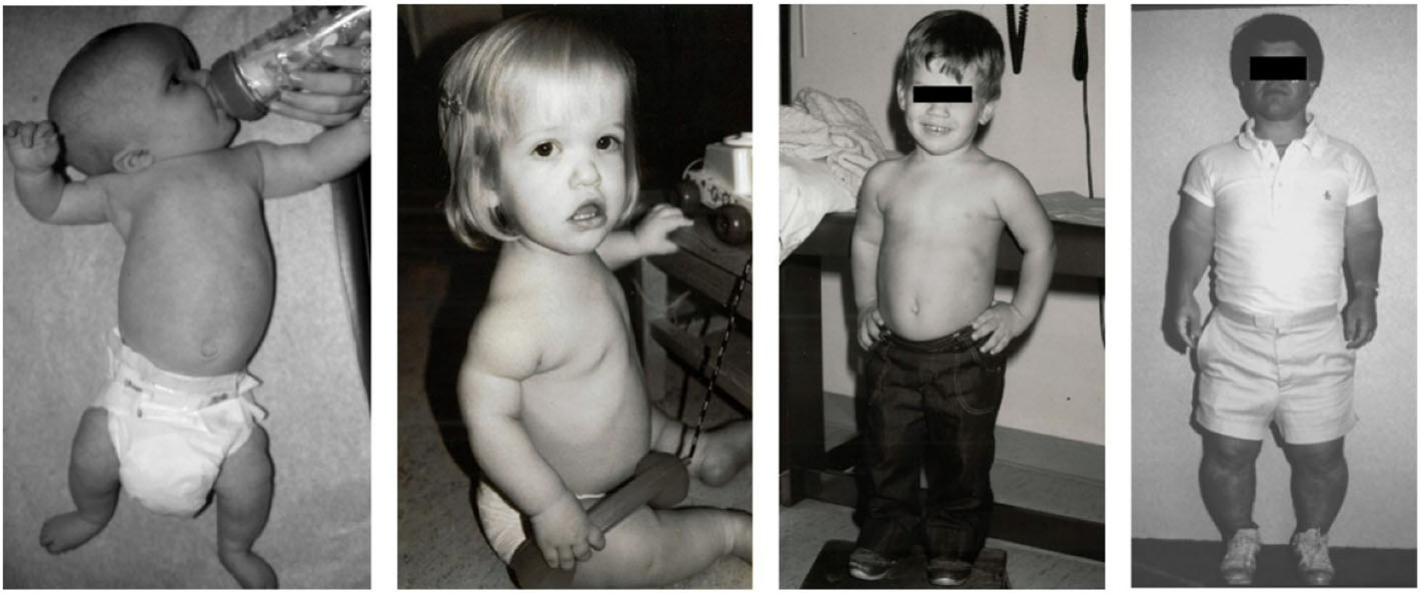
The body phenotype is shown in individuals of different ages: Left to right – infancy, early childhood, childhood and adulthood. In all, note the rhizomelic shortening of the limbs, which are disproportionately short compared with the trunk. In the infant and young child macrocephaly is evident

**Fig. 3:**
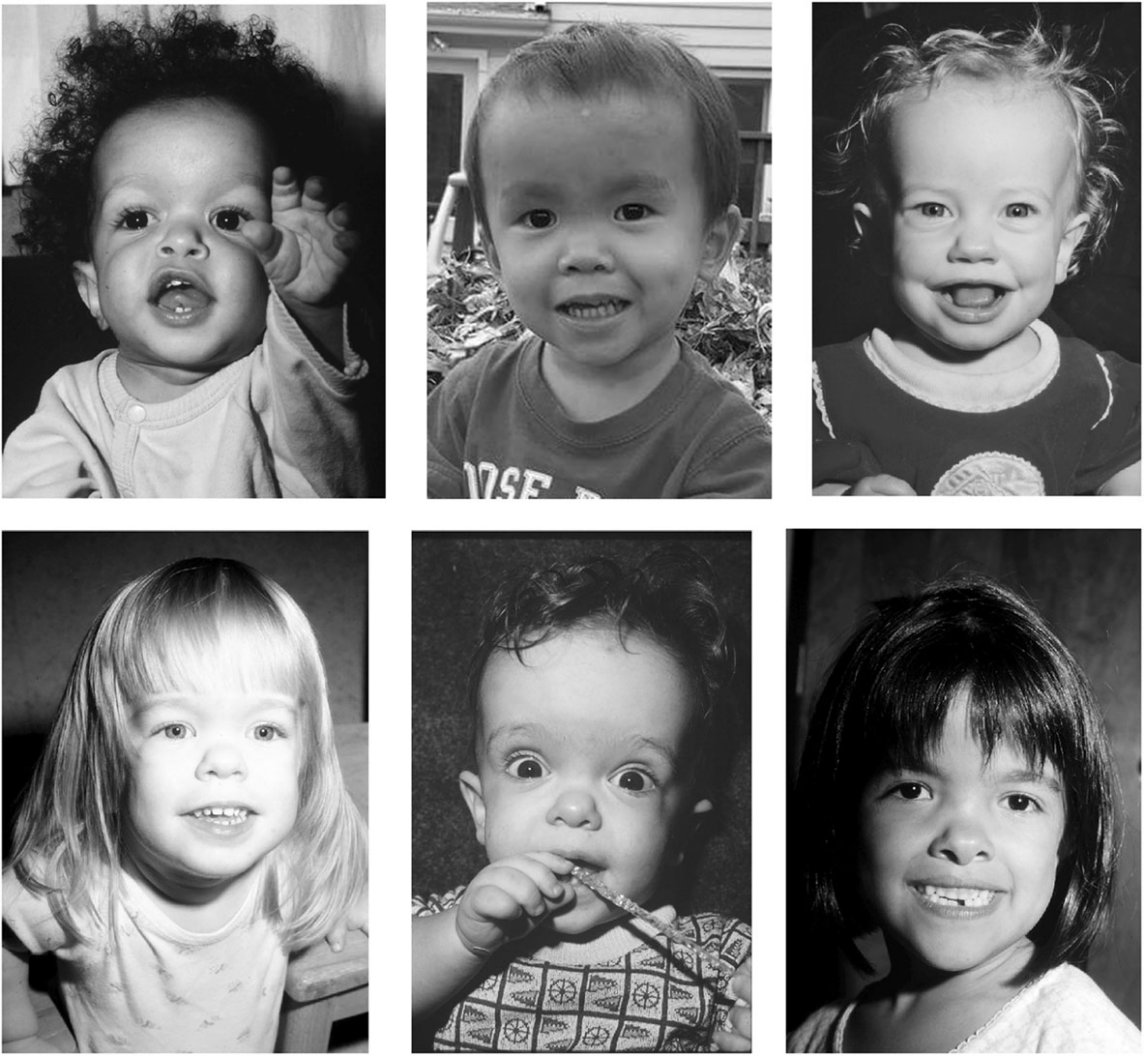
Six portraits of children with achondroplasia. The variability of craniofacial features is evident. Lower left and lower center photographs originally published in Pauli RM (1995) Osteochondrodysplasias with mild clinical manifestations: A guide for endocrinologists and others. Growth Genet Horm 11:1–5

**Fig. 4:**
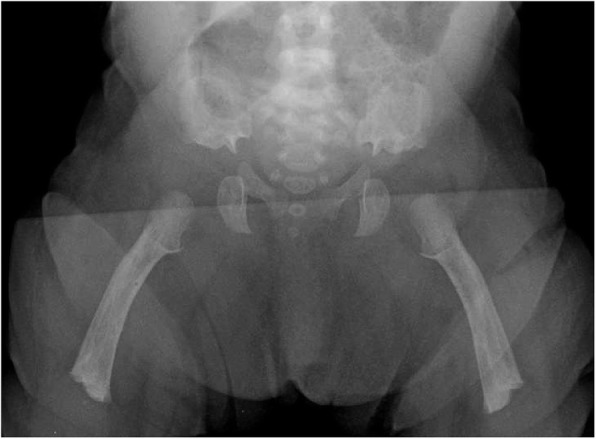
Anteroposterior radiograph of the pelvis and femora in an infant with achondroplasia. Characteristic findings include a squared-off pelvis, horizontal acetabula, very narrow sacrosciatic notch, characteristic proximal femoral radiolucency, and short and robust femora

**Fig. 5:**
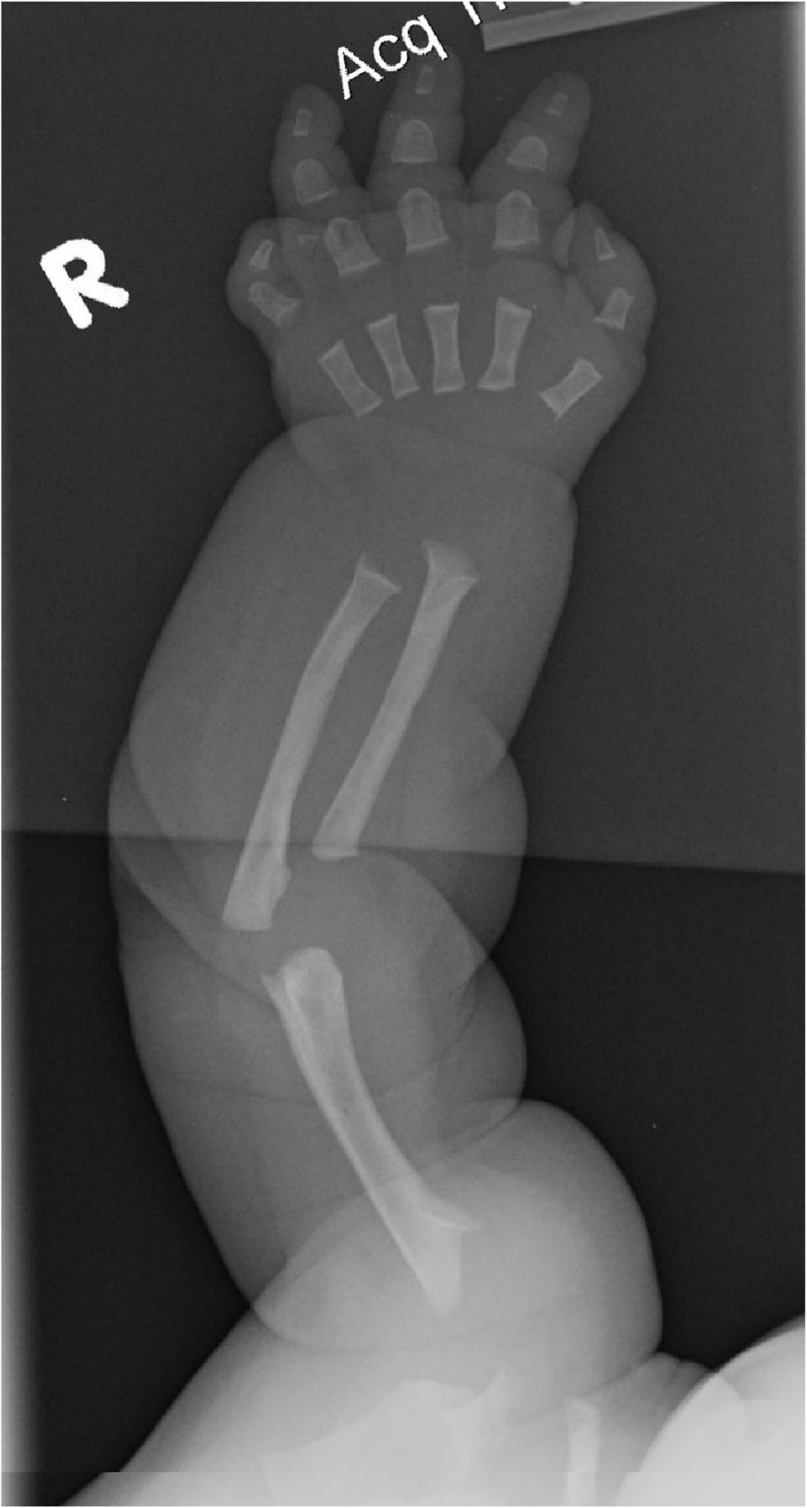
Arm radiograph in a newborn with achondroplasia. Although there are generalized metaphyseal abnormalities and shortening of all of the long bones, characteristics here are not as diagnostically helpful as those shown in Fig. 4

**Fig. 6:**
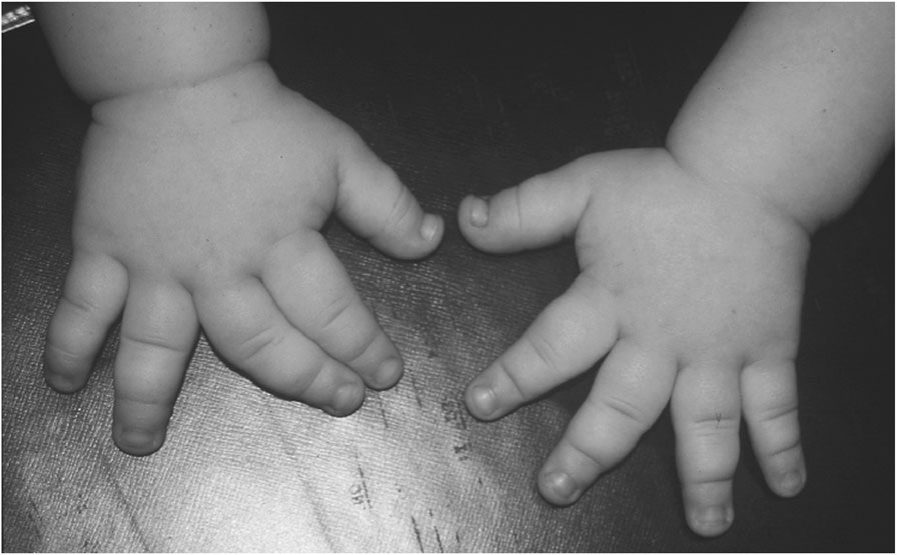
Hands in achondroplasia, well illustrating brachydactyly and (here, asymmetric) trident configuration – excess separation between the third and fourth fingers. Originally published in Pauli RM (2010) Achondroplasia. In Cassidy SB, Allanson JE: Management of Genetic Syndromes, 3rd ed. Wiley-Blackwell

Clinical features (Figs. 2 and 3) and radiologic characteristics (Figs. 4 and 5) are listed here with some comments. Clinical features include:

- *Small stature.* Small size is not a constant feature in infants, who may have lengths within the normal range [61].
- *Short limbs and rhizomelic disproportion.* Rhizomelic (proximal) shortening is uniformly present (at least in the arms [12, 62]), although variable in severity. Often there are redundant skin folds of the upper arms and the thighs.
- *Macrocephaly.* Head size is usually large at birth and remains so throughout life [61]. Variable frontal and parietal bossing (prominence and bumpy protuberance) is usually present (Fig. 3). The anterior fontanel is often large in infancy and may persist to as late as 5 or 6 years of age.
- *Midfacial retrusion.* Underdevelopment of cartilaginous bones of the face result in flattening of the entire midface and a flat nasal bridge, a short nasal spine and anteversion of the nose (Fig. 3).
- *Small chest.* In addition to the chest being often smaller than average [63], the ribs are overly compliant. This results in paradoxical movement with inspiration, which is often misinterpreted as being retractions reflecting respiratory distress.
- *Thoracolumbar kyphosis.* Virtually all infants develop a dynamic thoracolumbar kyphosis in infancy [64], but this is not present at birth.
- *Lumbar hyperlordosis.* Exaggerated lordosis (“swayback”) arises when walking begins.
- *Limited elbow extension.* Unlike most other joints, the elbows are stiff and may, with age, become progressively stiffer.
- *Short fingers and trident configuration of the hands* (Fig. 6).
- *Hypermobile hips and knees.*
- *Bowing of the mesial segment of the legs.* Bowing is not congenital. It most often arises in early childhood and may progress at unpredictable rate and extent until growth is completed.
- *Hypotonia.* Most infants with achondroplasia are hypotonic [65]. The combination of joint hypermobility and hypotonia means that many infants will seem particularly “floppy”.

In summary, those features that are diagnostically most helpful in the neonate and young infant include: *rhizomelic shortening of the arms; macrocephaly; midfacial hypoplasia and nasal anteversion; small chest; short fingers and trident configuration; hypermobility of the hips and knees; hypotonia.* Not all infants will display all of these features.

Diagnostic confirmation requires radiographic assessment. Although achondroplasia is a metaphyseal dysplasia, generalized metaphyseal changes are mild and nonspecific. Diagnostically helpful features include: short, robust tubular (“long”) bones; squared off iliac wings; flat, horizontal acetabula; marked narrowing of the sacrosciatic notch; a characteristic proximal femoral radiolucency; narrowing of the interpediculate distance of the caudal spine (although often not present in the neonate); short proximal and middle phalanges [12]. Typically a complete skeletal survey (or a hemi-survey of one side of the body) will be obtained (Figs. 4 and 5). However, most of the diagnostically critical features will be appreciated on a single anteroposterior radiograph of the pelvis and femora (Fig. 4).

Only rarely should diagnostic uncertainty remain after careful clinical and radiologic assessment. When needed, molecular testing is straightforward. Because nearly all instances of achondroplasia arise from a change in the same base pair of *FGFR3* [40], targeted mutation analysis is the routinely employed molecular test. Around 98% of persons with achondroplasia will have a c.1138G>A gene change, and 1% or so will have a c.1138G>C mutation [7]. Testing is available commercially from a large number of laboratories. Only very rarely and in very unusual circumstances will any additional molecular testing be warranted.

On rare occasions, when molecular confirmation has been sought, a common mutation will not be found. In such an event, further analysis of *FGFR3* is warranted [66], since occasional instances of other *FGFR3* pathogenic variants have been documented previously [67–71]. Note, however, that in some of these there is inadequate clinical documentation [67, 69], while in others such as the case described by Takagi et al. [70] the phenotype is, in fact, inconsistent with typical achondroplasia.

### Differential diagnosis

In the most general sense, any short limb dwarfing disorder would fall within the spectrum of the differential diagnosis of achondroplasia. Only a few conditions are likely to result in any substantial confusion, however.

#### Allelic conditions

**Fig. 7:**
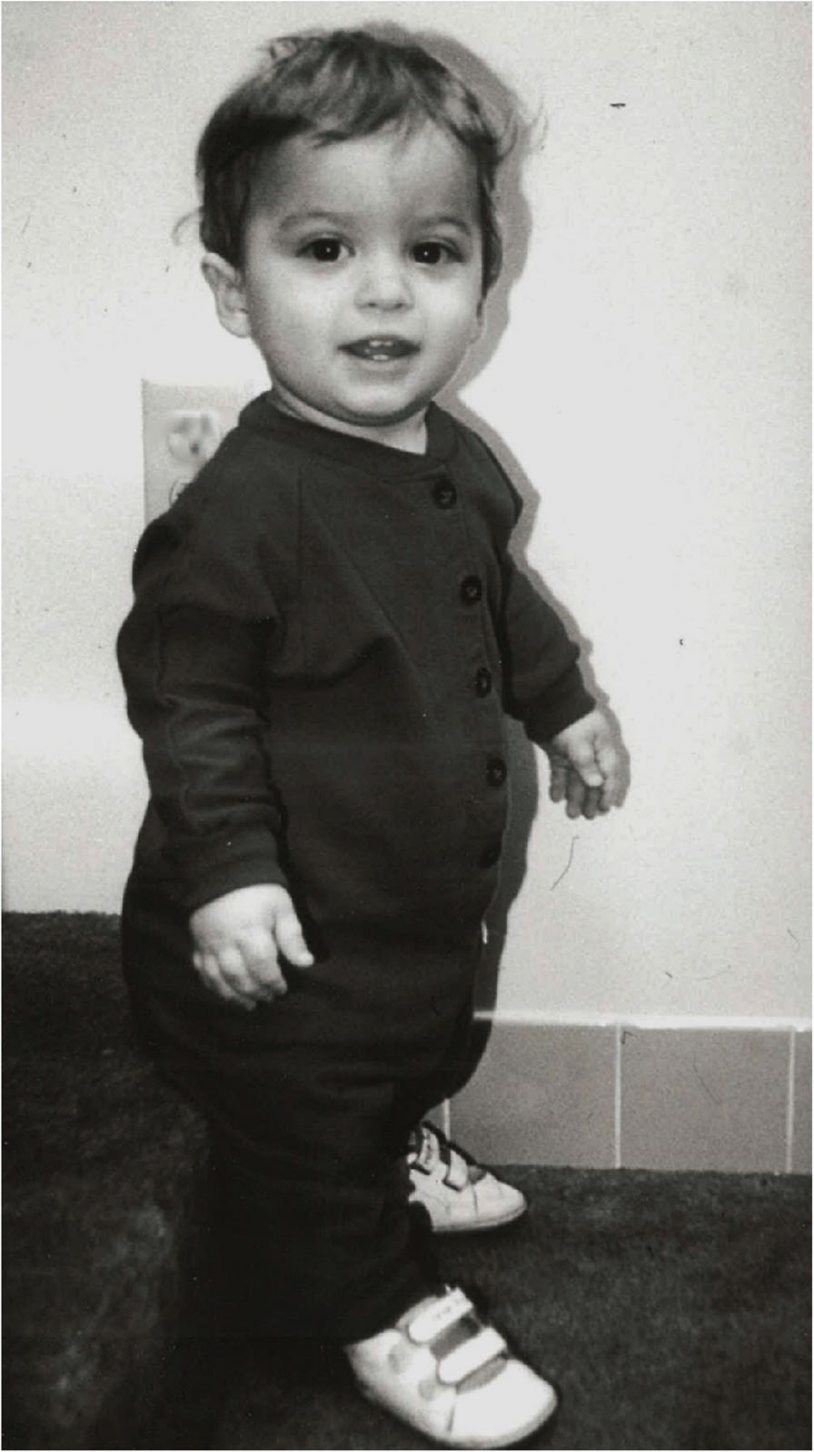
General body habitus present in hypochondroplasia. Cursory examination could easily miss the presence of a bone growth disorder in such a child. Originally published in Pauli RM (1995) Osteochondrodysplasias with mild clinical manifestations: A guide for endocrinologists and others. Growth Genet Horm 11:1–5

Distinct mutations in *FGFR3* may cause a number of allied conditions with shared features and differing mostly in severity [52]. The most important of these is hypochondroplasia (Fig. 7). Hypochondroplasia has been recognized as a distinct clinical entity for only around 50 years [72–79]. While in general clinically significant sequelae are less frequent and less severe than seen in achondroplasia [80], hypochondroplasia is not simply “achondroplasia but milder.” On the one hand, there is a virtual continuum of severity: achondroplasia > severe hypochondroplasia > mild hypochondroplasia > short stature with minimal or no [81] body disproprortion > normal. On the other hand, that the natural history of these two disorders is in certain ways in fact quite different makes issues of differentiating between them in any particular patient sometimes difficult but often critically important [82]. For example, temporal lobe dysgenesis, seizures and cognitive abnormalities are far more common in those with hypochondroplasia [82–84], while issues related to the craniocervical junction are far less frequent in hypochondroplasia than in achondroplasia. Clinically, marked craniofacial disproportion is much less common in hypochondroplasia than in achondroplasia, and the severity of rhizomelia and brachydactyly generally less than that seen in achondroplasia. Radiologically, all features seen in those with hypochondroplasia are also present in those with achondroplasia. However, three radiologic features uniformly present in achondroplasia but virtually never evident in hypochondroplasia help with this distinction: the characteristic proximal femoral radiolucency of achondroplasia is rarely evident in those with hypochondroplasia; rhizomelic disproportion of the arms, uniform in achondroplasia, is usually absent in hypochondroplasia when ratios of long bone lengths are assessed [85]; the moderate to marked abnormalities of facial bone contour of achondroplasia are not present in those with hypochondroplasia.

Nonetheless, occasionally molecular testing is warranted in distinguishing hypochondroplasia and achondroplasia. If a child being assessed clearly has *either* achondroplasia *or* hypochondroplasia but it is uncertain which of these is present, the most parsimonious approach is to test for the achondroplasia pathogenic variant first. If it is present, the diagnosis is confirmed. If absent (and since virtually all individuals with achondroplasia have the so-called common mutation) and the child clearly has one or the other of these diagnoses, then a diagnosis of hypochondroplasia can be made. With such a result, hypochondroplasia may have arisen either because of a mutation in *FGFR3* or at some other locus, but making that distinction is not nearly so important as making the distinction between achondroplasia and hypochondroplasia.

**Fig. 8:**
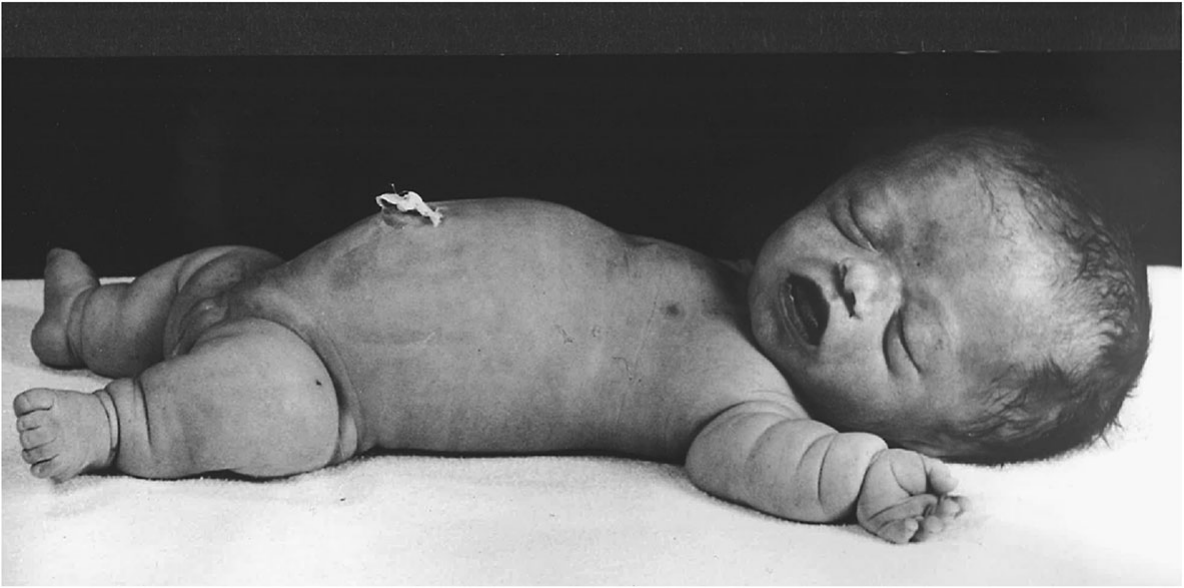
Clinical phenotype of thanatophoric dysplasia, type I. All features are far more severe than those seen in babies with achondroplasia (Fig. 2)

**Fig. 9:**
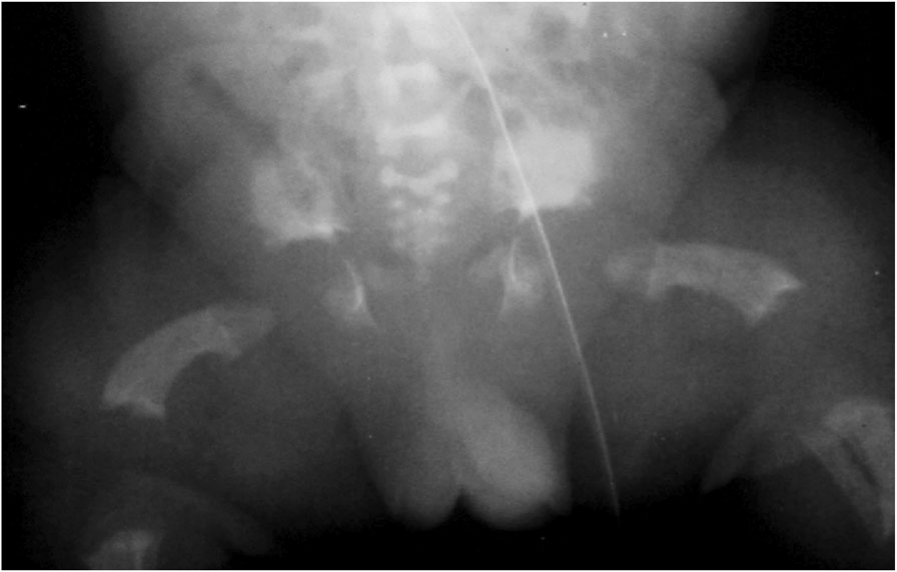
Anteroposterior radiograph of the pelvis and femora in thanatophoric dysplasia, type I. Here, too, qualitatively most of the abnormal characteristics are similar to those seen in achondroplasia, but quantitatively all of them are much more severe. Note the so-called telephone receiver femora

Thanatophoric dysplasia [86, 87] was originally described by Maroteaux et al. [88]. As the meaning of its name implies – “death bearing dwarfism” – it is usually lethal, usually in early infancy. It is probably about as com6mon as is achondroplasia [15, 89]. The clinical and radiographic characteristics are uniformly similar to, but much more severe than the same characteristics in achondroplasia (Figs. 8 and 9). There are two forms of thanatophoric dysplasia. Type I has curved, “telephone receiver” femora (Figs. 8 and 9) and very flat vertebral bodies, while type II has straight femora, taller vertebrae and virtually always has severe craniosynostosis [90, 91]. Both are caused by distinct mutations in *FGFR3*. Rarely should there be diagnostic confusion between thanatophoric dysplasia and achondroplasia.

**Fig. 10:**
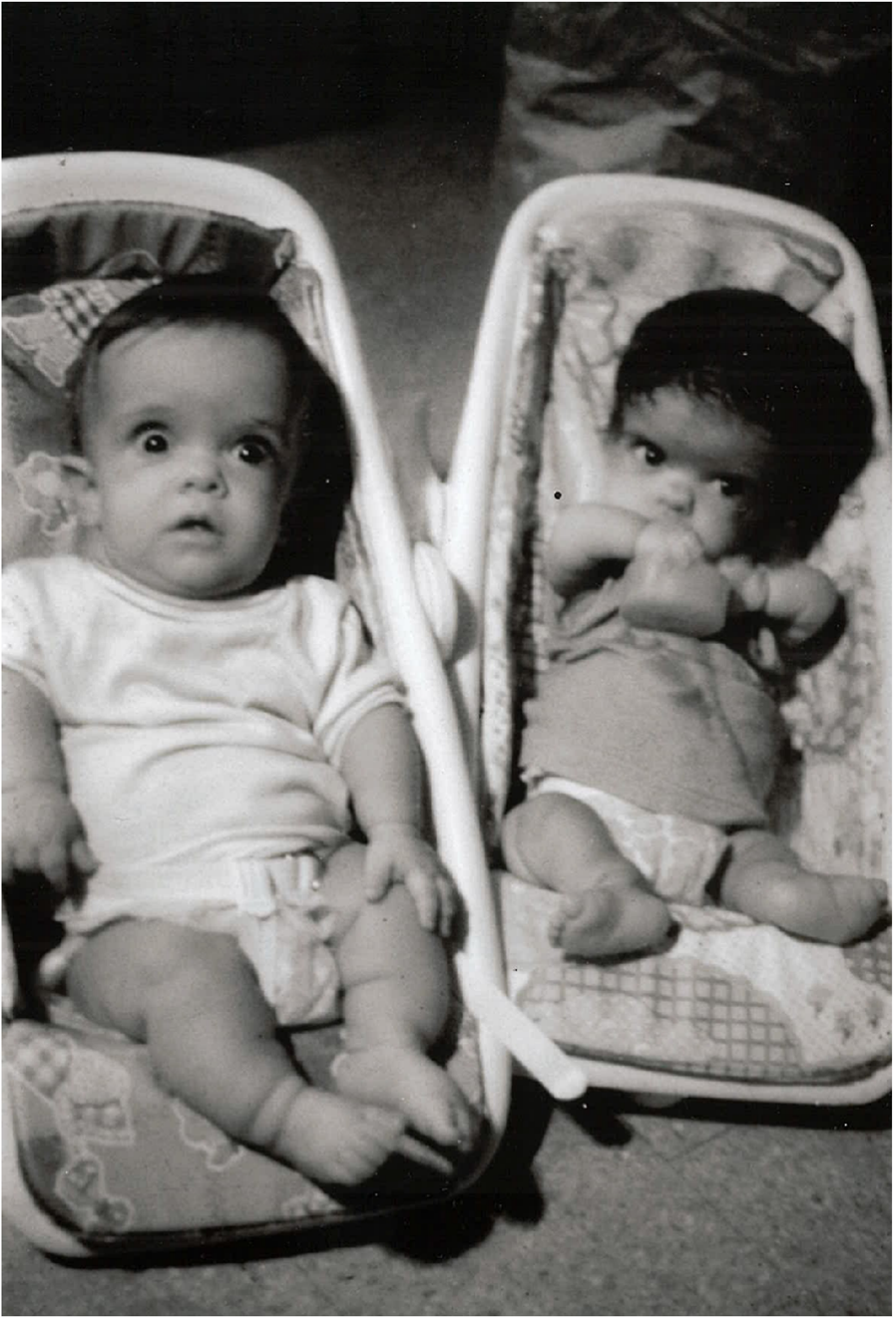
Siblings. On the left is an infant with typical, heterozygous achondroplasia. On the right is his older sister who had homozygous achondroplasia. Note the far greater limb foreshortening and much smaller stature in the latter

Homozygous achondroplasia (Fig. 10) (see above) is quite similar to thanatophoric dysplasia. Of course, it only arises if both parents have heterozygous achondroplasia. (Theoretically, it should arise rarely secondary to a new mutation when only on parent has achondroplasia, or secondary to two mutational events when neither parent has achondroplasia, but those probabilities are remote; in fact, neither event has to date been reported.) Like thanatophoric dysplasia, this should rarely cause diagnostic confusion.

SADDAN syndrome [92–94] is most infelicitously named. “SADDAN” stands for “severe achondroplasia with developmental delay and acanthosis nigricans”. It uniformly results from a mutation that causes a Lys650Met substitution in *FGFR3*. Prior to the age at which developmental disability can be recognized and before acanthosis nigricans develops, confidently differentiating achondroplasia and SADDAN syndrome requires molecular evaluation. Such assessment should be pursued, particularly in instances in which global developmental delays more severe than those typically seen in achondroplasia are identified.

A number of other rare dysplasias secondary to *FGFR3* mutations have been described (e.g., see [31, 95, 96]). None is likely to be encountered.

In addition to the *FGFR3* family of bone dysplasias, other mutations in this same gene can cause Crouzon syndrome with acanthosis nigricans [97], Muenke syndrome [98], isolated acanthosis nigricans with or without slow linear growth [99–101], and slow linear growth without unequivocal features of a bone dysplasia being present [81]. Loss of function mutations (in contrast to the gain of function that results in achondroplasia) cause an overgrowth disorder in both sheep [102] and humans [103].

#### Other conditions

Achondroplasia is a metaphyseal dysplasia. Generally, however, other metaphyseal dysplasias, such as the Schmid type of metaphyseal dysplasia [104] and cartilage-hair hypoplasia [105] are straightforwardly distinguished by clinical features, radiographic features and age of presentation.

Any rhizomelic dwarfing process might occasionally cause diagnostic confusion. Pseudoachondroplasia [106] deserves mention. Despite its name, it is primarily a spondyloepiphyseal dysplasia sharing little except rhizomelic dwarfism with achondroplasia. It has none of the craniofacial features that are present in achondroplasia. It is typically not diagnosed until the 2nd or 3rd year of life. Radiographs are utterly dissimilar.

## Survival

Most of those with achondroplasia will have a normal or near normal life expectancy. However, there is an increased risk for premature death [107–109] related not only to sudden unexpected deaths in infancy (see below) but also, it appears, to cardiovascular complications in mid-adult life [108]. Overall, average life span is about 10 years less than that of the general population [107]. A recently completed study [110] confirms that the highest standard mortality rates are in those less than 4 years of age. However, in addition, that multicenter mortality study shows that there has been a dramatic decrease in deaths, including sudden unexpected deaths, in young children with achondroplasia, most likely secondary to recognition of their special risks and aggressive evaluation and intervention related to the craniocervical junction [110].

## Natural history and management

Most of the medical issues that need to be addressed in individuals with achondroplasia are presented by organ system, below. However, there are two concerns – craniocervical junction constriction and restrictive pulmonary disease – that may be of major concern very early in infancy. These are summarized here. The first of these is a particularly important reason (along with parental needs) that diagnosis be confirmed as quickly as possible in infancy, so that critical evaluations can be completed in a timely manner.

### Special concerns in the young infant: the craniocervical junction

#### Initial recognition

The possibility that infants with achondroplasia are at increased risk for unexpected death was raised as early as 1982 by Pauli & Lebovitz [111] and Bland & Emery (cases 3 and 5) [112]. The single event that precipitated three-plus decades of investigation is as follows [113]. A baby boy was born to a mother of average stature and a father who had achondroplasia. Aside from achondroplasia, the boy was healthy and thrived until 3 months of age when he was found dead in his crib. He had been neurologically normal and had no antecedent illness. Postmortem assessment found no cause of death and a diagnosis of sudden infant death syndrome (SIDS) was made. The family was counseled that the baby’s achondroplasia and SIDS were almost certainly coincidental and unrelated. A new sister, also with achondroplasia, was born a year later. Not because of any suspicion that SIDS and achondroplasia were linked, but rather because of the then favored notion that there was strong familiality on a genetic basis for SIDS [114], polysomnography was completed. It showed substantial abnormality of central respiratory control, which abnormalities resulted in two clinical apneic episodes requiring stimulation, but which resolved by 5 months of age.

**Fig. 11:**
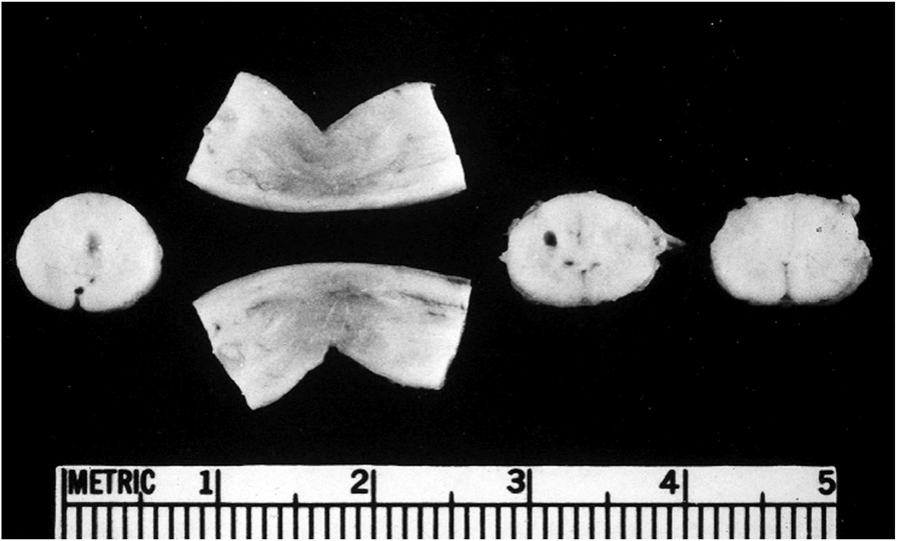
Gross pathologic features from the craniocervical junction of the spinal cord in an infant with achondroplasia who died suddenly and unexpectedly. There is gross indentation of the cord as well as cystic lesions secondary to hypoxic damage. Originally published in Pauli RM et al. (1984) Apnea and sudden unexpected death in infants with achondroplasia. J Pediatr 104:342–348 [113]

**Fig. 12:**
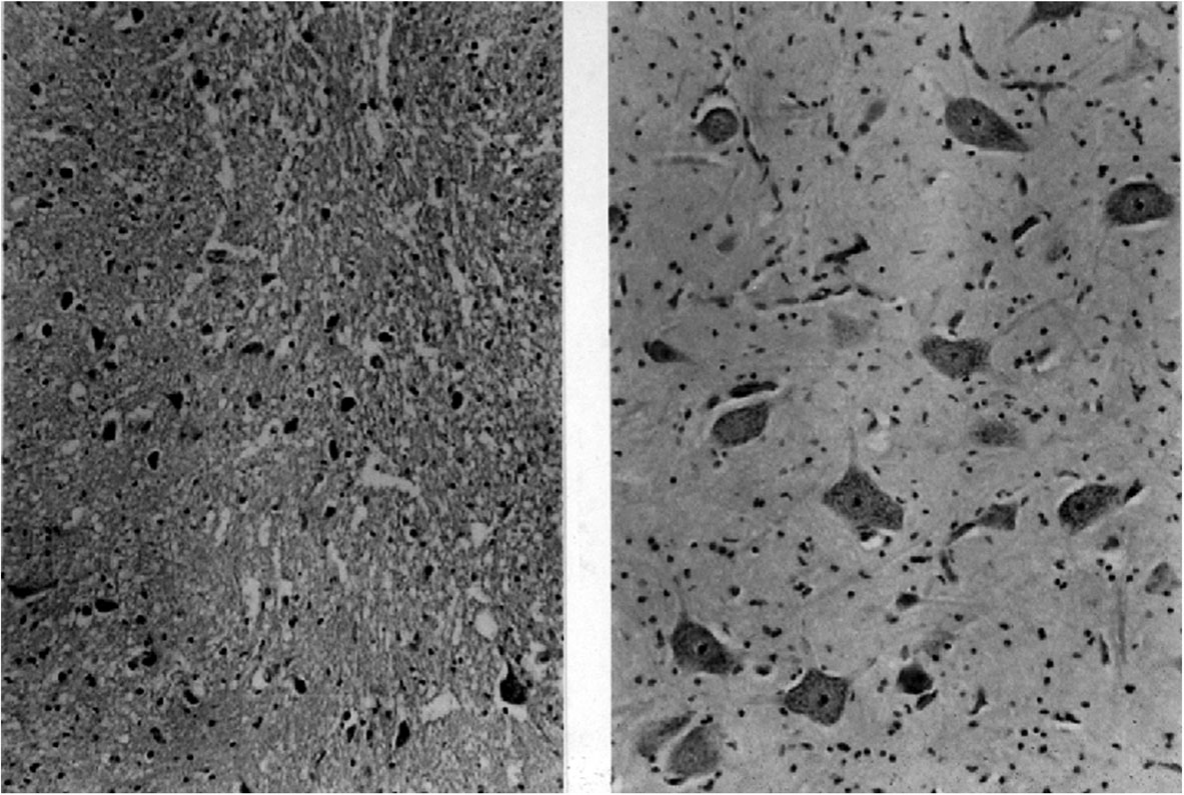
Histologic findings from the cervicomedullary junction in the infant described in the text. *Left* shows severe pyknosis secondary to hypoxic damage, compared with, *right*, a normal control of comparable age. Originally published in Pauli RM et al. (1984) Apnea and sudden unexpected death in infants with achondroplasia. J Pediatr 104:342–348 [113]

This led to consideration of the possibility that it was their shared diagnosis of achondroplasia that placed them at risk. A retrospective inquiry of 20 centers in which substantial numbers of individuals with achondroplasia had been evaluated yielded 10 additional patients with achondroplasia who had died unexpectedly [113]. Of those, four had evidence for severe damage to the medulla and upper cervical cord (Fig. 11). Subsequent reassessment of the craniocervical junction in the original proband showed that histologically he, too, had evidence of hypoxic damage (Fig. 12).

**Fig. 13:**
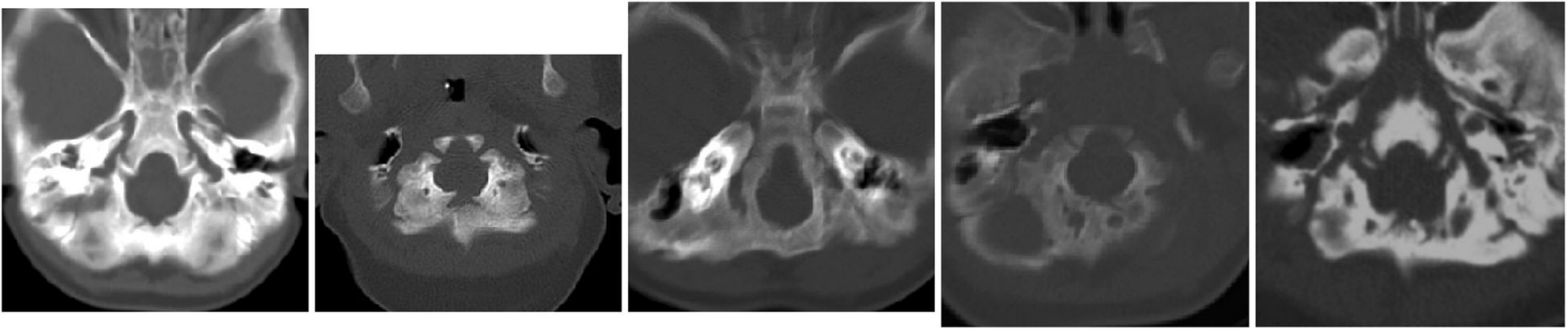
Computerized tomography in five infants with achondroplasia, demonstrating the variability of conformation of the foramen magnum

It was already known that infants with achondroplasia have decreased growth of the basicranium, which is of cartilaginous origin, and a small foramen magnum [115, 116]. The diminution of foraminal size arises directly from the decreased growth of cartilaginous bone as well as, perhaps, from abnormality of the synchondroses [117]. Furthermore, the foramen magnum is often abnormal in shape, frequently being “key holed” in appearance [8] (Fig. 13). This probably effectively diminishes even further the space actually available. Although direct compression of the spinal cord can occur (see below), it is more likely that the apneic deaths arise from compression of vertebral arteries at or near the craniocervical junction. The events surrounding the deaths included ones in which uncontrolled head movement could have resulted in craniocervical compression. Therefore, we postulated that those deaths arose from either acute or chronic compression of vasculature at the craniocervical junction resulting in hypoxic damage to the central respiratory control centers in the medulla. In turn, such hypoxic damage can result in diminished central respiratory control, and, in the most severe instances, irreversible apnea.

All of the deaths in the initial report were between 3 and 11 months of age [113], suggesting that if preventive assessments are to be effective they must be completed early in infancy.

Subsequent experience has clearly demonstrated that without careful assessment some infants with achondroplasia will die because of craniocervical junction issues [8, 118]. A number of studies have provided important additional information. For example, Reid et al. [118] confirmed that craniocervical compression can cause non-lethal but severe respiratory problems, and that a complex interplay of restrictive, obstructive and centrally caused respiratory issues in infants with achondroplasia can be difficult to sort out in practice. They also showed that the non-lethal respiratory problems were alleviated by suboccipital decompressive surgery [118]. Although the interpretation by Tasker et al. [119] of their patients’ signs and symptoms are likely in some ways incorrect, they did demonstrate that basicranial hypoplasia seems not only to cause central apnea but can also result in obstructive apnea [119] (what can be termed centrally mediated obstructive apnea); this might also be the reason for the observations of Sano et al. [120], which are otherwise inexplicable. Further, Tasker et al. pointed out that gastroesophageal reflux can additionally complicate the picture in infants with achondroplasia [119].

#### Estimate of risk

Although the risk of death remains uncertain, consensus has developed that it is substantial. Hecht and her colleagues [107, 108] have estimated that the risk for death in the first year of life may be as high as 7.5%. While that may be a maximal risk and may, indeed, be an overestimate, nonetheless, without special assessment and, when needed, surgical intervention, that risk is likely at least 2–3%. Although there was early disagreement about whether this is a real phenomenon [121], subsequently a consensus arose (D. Rimoin, personal communication, 2004) at least to the fact that this is a real concern.

#### Prospective assessment of level of risk and of risk factors

Between 1983 and 1994 we prospectively evaluated 53 infants with achondroplasia who were referred without explicit neurologic or respiratory concerns [8]. Uniform, comprehensive assessments demonstrated that 5 of the 53 had problems of sufficient severity to justify suboccipital decompressive surgery. In every such instance, marked abnormality of the upper cervical cord was demonstrated intraoperatively. Replicable predictors of need for decompression included: (1) small foramen magnum compared with achondroplasia standards [122]; (2) hyperreflexia and/or clonus; (3) central hypopneic events resulting in oxygen saturations below 0.85 by polysomnography [8]. Therefore, anatomic, neurologic and respiratory characteristics, together, allow identification of those babies who likely are at highest risk to experience life-threatening events related to the craniocervical junction.

#### Recommended evaluation – standard assessment

As already noted, if any assessment is to make a difference regarding sudden unexpected deaths, it must be completed very early in life, preferably prior to 3 months of age [123].

A ‘standard’ assessment needs to include the following components [4]:

- ▪ Neurologic history and neurologic examination, the latter emphasizing whether there is hypotonia more severe than typical for babies with achondroplasia, and whether hyperreflexia or clonus is demonstrable.
- ▪ Imaging of the craniocervical junction. Initially this was most often by computerized tomography [124] (Fig. 13). This approach continues to offer certain advantages. First, it allows measurement of the size of the foramen for which standards are available [122] and which was a demonstrable predictor of need for decompression [8]. Secondly, most often it can be completed without sedation or anesthesia. This may be a serious consideration given concerns about the effects on synaptogenesis of anesthesia in young babies [125].
- ▪ Polysomnography (sleep study). This should be performed in a sleep center comfortable with assessing infants. Interpretation can be complicated by restrictive pulmonary issues with decreased respiratory reserve. Emphasis, of course, should be on assessing *central* apnea and hypopnea.

**Fig. 14:**
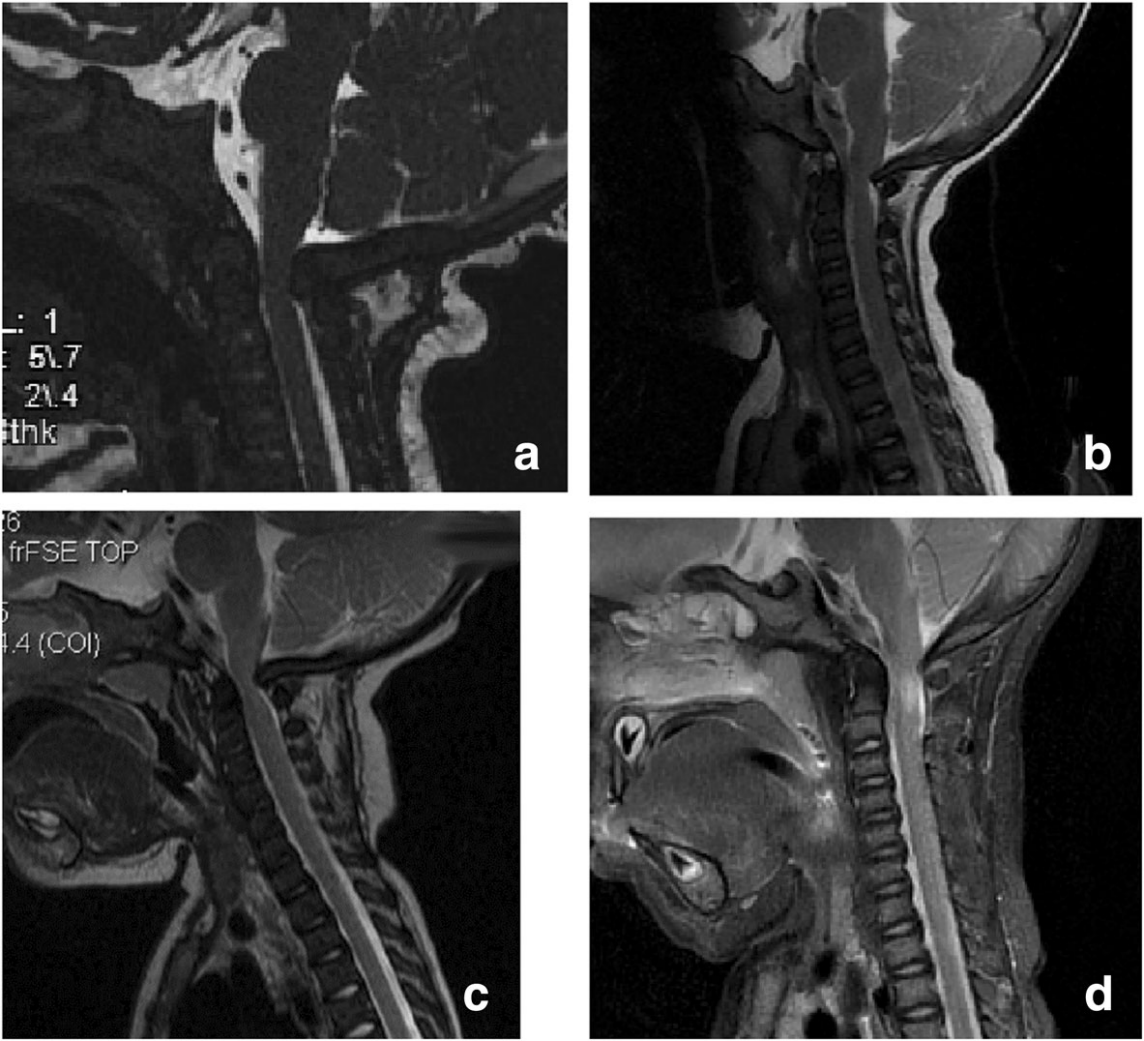
Sagittal views of magnetic resonance imaging of the cervical cord in four infants with achondroplasia. **a** There is obliteration of the posterior subarachnoid fluid layer; **b** In addition to obliteration of the fluid layer posteriorly, there is “nicking” of the posterior cord; **c** Narrowing of the cord is evident secondary to “waisting” – that is, there is some indentation anteriorly, as well; **d** An obvious T2 signal abnormality is present

In our center, only in infants who have worrisome features based on these initial assessments is magnetic resonance imaging (MRI) completed. Generally, we now obtain the MRI in both flexion and extension [126, 127]. MRI, too, requires careful interpretation specific to achondroplasia. All infants will have narrowing at the foramen magnum. Most infants with achondroplasia will have obliteration of the posterior subarachnoid fluid layer (Fig. 14). Some will have posterior ‘nicking’ or ‘waisting’ of the cord (Fig. 14). These features must be interpreted in light of clinical characteristics, since clearly some babies with all of these features do well and thrive without decompressive surgery (personal observations). Prudence commends that MRI studies be reviewed by a neuroradiologist with experience and expertise in achondroplasia in order to limit unneeded surgeries. The presence of either a T-2 signal abnormality (Fig. 14) or a syrinx probably justifies surgical intervention regardless of clinical status.

#### Alternative approaches to initial assessment

Various alternatives have been suggested and used. Note that none has prospectively demonstrated utility, but are mostly based on ‘reasonableness’. Such prospective investigations of what evaluation scheme is most helpful are desperately needed but very difficult to develop.

Many centers have elected to proceed directly to MRI rather than CT [128]. Better visualization of neural tissue is forthcoming, but usually sedation or general anesthesia will be needed because of the length of the procedure. Often multiposition MRI is elected [126, 129]. Flow studies may be of some help as well in determining whether surgical intervention is warranted [126, 128]. Three dimensional CT might be another alternative [130], as might be diffusion tensor MRI imaging [131]. Another alternative that has been considered is so-called fast-MRI (or quick-MRI) [132]. This obviates the need for anesthesia and the possible risks that this entails both immediate and, at least speculatively, long term [133]. However, while sufficient for many purposes, detail obtained by fast-MRI is not sufficient to definitively determine the need for surgery related to the craniocervical junction.

It has even been suggested that no imaging at all be routinely done in infants with achondroplasia [134]. However, this recommendation appears to be based on no objective, published evidence [135].

Table 1 summarizes the advantages and disadvantages of various approaches to imaging in infancy. There is clear need to objectively assess which approach or approaches are most advantageous. At a minimum, standards for MRI (or fast-MRI) features and measurements in infants with achondroplasia need to be generated [136], if this is to become the routine method of anatomic evaluation.

**Table 1:** Comparison of advantages and disadvantages of various imaging approaches in young infants with achondroplasia

Study:	Advantages	Disadvantages	Comments
CT	Usually not requiring sedation	Poor delineation of neural structures	
	Only study method for which there are diagnosis-specific standards (FM size) [122]	Substantial radiation exposure, particularly if not performed in children’s facility	
	Only study method for which prospective assessment of value in ascertaining risk is available [8]	In substantial minority, along with results of other non-radiologic studies, will lead to MRI subsequently	In our experience, about 20% of those following protocol including CT will go on to MRI
MRI	Excellent delineation of neural structures	Virtually always requires sedation or anesthesia	Because of respiratory concerns present in most infants with achondroplasia, anesthesia is usually needed
	No radiation exposure	No diagnosis-specific standards	
		Substantial risk of over-reliance in determining if surgery is needed	Although not prevalent in our center, there are many anecdotes of electing to have decompressive surgery based on MRI craniocervical features alone, which we would judge to be non-actionable without other indications
Fast MRI	Fair delineation of neural structures	Detail may be insufficient for decision making	
	No radiation exposure	No diagnosis-specific standards	
	No sedation or anesthesia needed	In substantial minority will lead to full MRI before deciding if surgery is needed	At current level of detail, findings on fast MRI will always need to be confirmed by routine MRI if surgery is contemplated
No routine imaging	No sedation or anesthesia	Under-ascertainment of those needing decompression	This is, in my opinion, an unacceptable risk
	No radiation exposure	Ignores what prospective and reasonably well controlled trial data as are available to prevent further neurologic injury or sudden death.	

Some have also suggested a step-wise protocol. For example, one might only image those children in whom worrisome features are present by clinical examination and/or polysomnography (M. Bober, personal communication 2014). While logical, such stepwise assessment has not yet been rigorously assessed and should not be embraced without evidence to support it. Likewise, the suggestion that polysomnography is not predictive of craniocervical junction concern, and so implying that it is not an essential assessment [137], is based on a small, retrospective series of patients, of whom many were well outside the age range of relevance, and is not worthy of further consideration.

Somatosensory evoked potentials could be of considerable benefit in identifying infants at high risk. Early experience, however, suggested that most infants with achondroplasia showed abnormalities of somatosensory evoked potentials, and that it failed to discriminate between those at high risk and others [8]. However, other investigations suggest that there may be a role of evoked potentials in the assessment of the craniocervical junction in infants with achondroplasia [138–140]. Should any prospective studies of efficacy of evaluations be initiated in the future, somatosensory evoked potential testing should probably be included in such a protocol.

#### Prevention

Counseling regarding cautions that should be exercised with every infant with achondroplasia are based, in part, on the presumed mechanism of injury at the craniocervical junction and, in part, on the circumstances that have been observed in instances of unexpected infant deaths. As noted, risk likely is related to a combination of foramen magnum constriction, the typically large head of achondroplasia and poor head control, which often takes longer to develop in infants with achondroplasia. Uncontrolled head movement should, then, be avoided. There is anecdotal evidence that deaths are particularly likely to arise in babies who have been placed in vertical automatic swings [4, 113]; in fact, I am aware of at least six instances in which babies with achondroplasia have died in vertical automatic swings. There also have been multiple instances of life-taking or life-threatening events in car seats [141] (and personal observations).

Precautions should include:

- Careful head and neck support, particularly with transitions.
- Prohibition of automatic swings (“swingomatics”).
- Use of solid back strollers rather than umbrella strollers that may more forcibly flex the neck.
- Use of a neck roll in strollers, and, particularly, in car seats. When restrained, infants with achondroplasia, who have large and prominent occiputs, will have their necks in a forced flexed position.

#### Management

In those infants where assessment demonstrates unequivocal cord compression resulting in clinical abnormalities, then suboccipital decompression should be completed urgently [142, 143]. Operative intervention may be particularly challenging because of the unique anatomy of the skull in achondroplasia [116, 142].

At major centers in which large numbers of children with achondroplasia are evaluated, most report a decompressive surgery rate in infants of around 10% (11% [144]; 8% [145, 146]; 13% [147]; 10% at our institution). Although these rates likely exceed the total risk, it isn’t clear how they can be further reduced. That certain centers report far higher rates of surgery [141, 148–150] likely reflects referral biases and/or overly aggressive intervention. Major complications of decompressive surgery are rare [142] and the quality of life of those undergoing decompression is not compromised long term [151].

If, as suggested, such intervention is lifesaving, then with universal assessment and intervention 4–5 lives per year should be spared in the United States, and around 100 per year worldwide. As noted, there is evidence that routine assessment and intervention as outlined does decrease mortality in infants with achondropasia [110].

### Special concerns in the young infant: restrictive pulmonary disease

Infants with achondroplasia often have small chests [63]. In addition, there is increased compliance of the rib cage, sometimes dramatically so. That excess compliance is manifest as paradoxical movement with inspiration in most young infants with achondroplasia – sinking inward principally of the inferolateral part of the chest, but also often substernally. Mild deformity of the chest may also be present [152], including lateral indentations and pectus excavatum. The combination of these features – small chest, presumably but not certainly reflecting smaller anatomic lung volumes, inefficient chest mechanics, and chest deformity – together may result in smaller *effective* lung volumes.

Despite these features, most babies suffer no evident consequences. Predictably it does result in more rapid desaturations with physiologic sloppiness of central respiratory control or with minor obstructive events, making interpretation of polysomnography more challenging in young infants. Small chest volume also causes diaphragmatic descent and “pseudo-organomegaly” (with a liver edge often palpable well below the inferior costal margin).

In a small proportion this set of features can result in chronic hypoxemia. This is particularly likely in those living at high altitude (personal observations). Chronic hypoxemia can be of sufficient severity to result in failure to thrive (presumably because of increased work of breathing), rarely respiratory failure, and, potentially, cor pulmonale if not addressed [118, 152, 153].

Assessment is straightforward. Clinically persistent marked tachypnea, secondary feeding difficulties because of that tachypnea, additional signs of chronic respiratory distress and failure to thrive may be present. In all babies with achondroplasia polysomnography needs to be completed for other indications (see above). This also will provide a long oximetry sample. Saturation dips into the high 80s are normal in infants with achondroplasia (personal observation). Low baseline oxygen saturation and/or frequent desaturations unaccompanied by recordable respiratory events will likely reflect restrictive pulmonary disease. In addition, daytime spot oximetries, both during active alert time and, particularly, during feedings for example, may be helpful. Chest circumference measures compared with achondroplasia standards may also be of some help [63].

In those with restrictive pulmonary disease, the help of a pediatric pulmonologist should be sought. Oxygen supplementation alone may be sufficient to maintain saturations and reverse failure to thrive. If not, tracheostomy may be needed. Restrictive pulmonary disease has been the principle condition resulting in need for tracheostomy in a small subset of babies with achondroplasia: At our institution ~ 1–2% of babies have required tracheostomy, primarily to eliminate dead space in those with restrictive disease. In all, it has been temporary.

### Growth

Given that the activating mutation of *FGFR3* that results in achondroplasia causes a general inhibition of endochondral bone growth, of course one would anticipate that all of the long bones of the body will grow slowly; and they do. Small stature is the signal characteristic of achondroplasia.

#### Linear growth

Although length at birth may be normal [61, 154], slow growth is evident shortly thereafter. Moderate to marked short stature is present in all affected individuals. In adult males average height is about 130 cm (4′3″) with a range from around 120 to 145 cm. Similarly, in females average height is 125 cm (4′1″) with a range of 115 to 137 cm. Average adult heights are about − 6 to − 7 S.D. below the mean for average stature individuals [155].

**Fig. 15:**
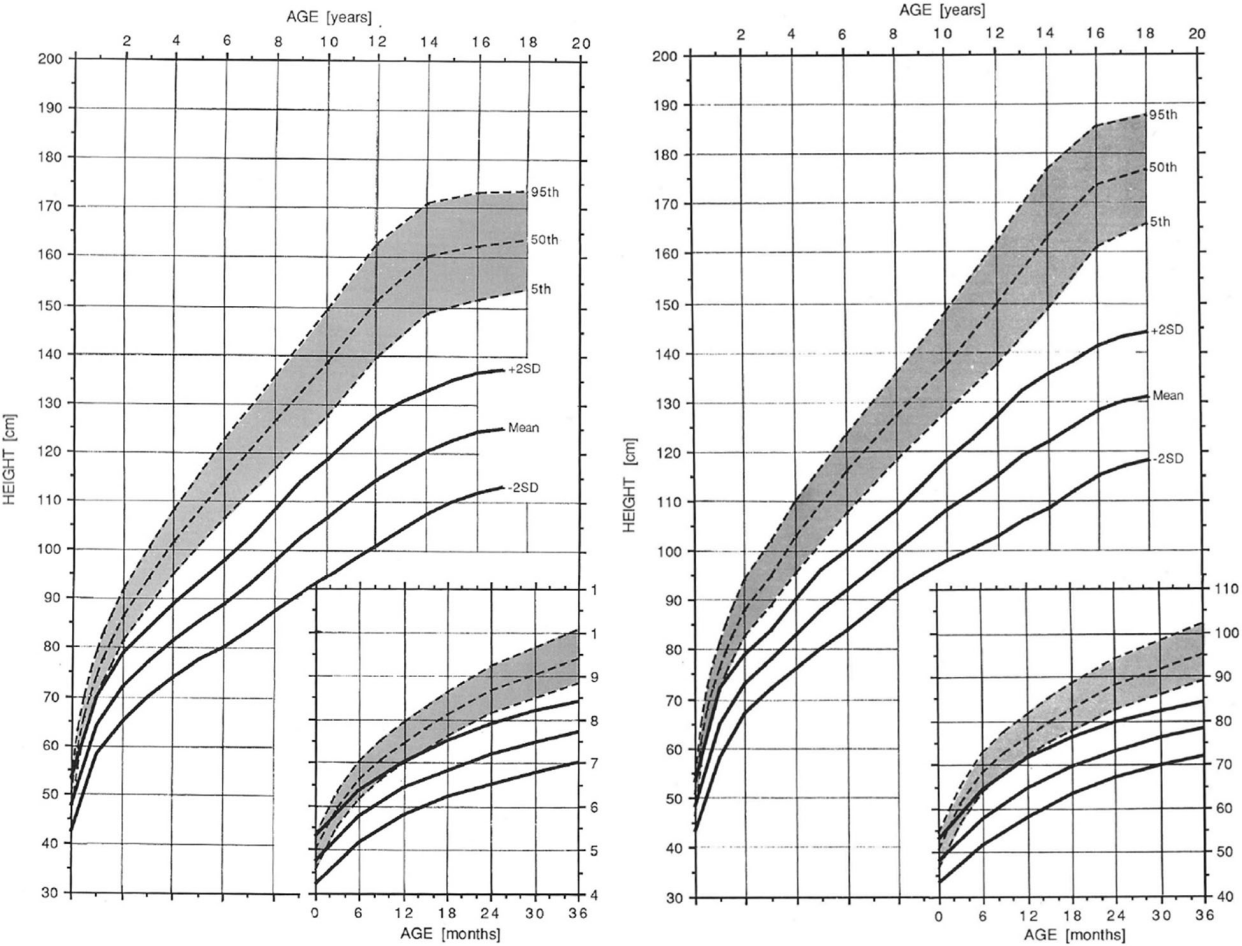
Diagnostic specific linear growth charts for females (*left*) and males (*right*) with achondroplasia. Comparable curves for average statured individuals are shaded. Reproduced with permission from Greenwood Genetics Center (1988) Growth References from Conception to Adulthood. Clinton, SC: Jacobs [156]

Remarkably few parents of average children understand the importance of routine measurement of growth – that growth is an excellent, nonspecific indication of general well-being. Plotting the growth of a child with achondroplasia on average stature charts will only confirm shortness and won’t offer the same opportunity to use growth as a measure of health as it is used in average statured individuals. Therefore, standard growth charts specific for achondroplasia [61, 156] should be used (Fig. 15), and length or height measured at each encounter with the child’s primary care provider. In addition to these hand-smoothed curves, statistically more rigorous growth curves for a U.S. population are available [154], which are of particular value since the supplied Z-scores allow translation of these growth charts into electronic records.

These standards are based on a U.S. population, and should be used with caution in other populations. Growth references for other populations are also available [157–159].

The achondroplasia mutation modifies rather than negates other genetic determinants of growth [160]. Height variability in individuals with achondroplasia seems to correlate just as strongly with parental heights as it is in average individuals. That is, tall parents will tend to have tall achondroplastic offspring and short parents, shorter than average achondroplastic children.

There is uncertainty whether individuals with achondroplasia do [155] or do not [154] show a normal adolescent growth spurt.

Small stature has substantial consequences for the affected individual. There may be psychological sequelae, which possibility needs to be addressed with parents and the affected individual. Physical limitations result, as well, and there will be need for environmental adaptations, particularly in school (see below). Note, however, that *sitting* height is near normal [161]. This is of relevance with respect to, for example, safe transitions from carseat to booster and regarding adaptive needs for driving.

##### Possible treatment of small stature

To date no treatment has been devised that will negate the effects on growth of achondroplasia (but see also Possible future therapies below). A substantial number of studies have been published regarding the use of growth hormone therapy to enhance growth in children with achondroplasia (for example [162–164]). A transient increase in growth velocity is predictably found, but the effect diminishes with length of treatment. Of the many published studies, virtually none has followed treated children to the completion of their growth [165]. However, Harada et al. recently described an uncontrolled observational study in which the final heights of achondroplasia patients who were treated with recombinant growth hormone were assessed. Following long-term therapy, average increase in adult height was + 2.8 cm in females and + 3.5 cm in males [166]. So, one can anticipate around 1 to 1 ½ inches of additional adult height following years of injections. Most families recognize the disadvantages of both daily injections and the accompanying medicalization of small stature, and few elect to pursue growth hormone therapy.

Extended limb lengthening is offered as another option for height enhancement [167–170]. A variety of techniques have been used, generally through osteotomies and gradual distraction using external fixators. Substantial lengthening and height enhancement can be achieved, so that potentially one can achieve an increase in adult height of 25 cm or more. This is a long and arduous process. High complication rates can be expected [170]. It has been used in many patients with achondroplasia; most of these lived outside of the United States, perhaps because of different attitudes toward outwardly evident physical differences. Most care providers and ethicists in this country have advocated that such surgery be postponed until the affected individual can participate in decision making (that is, in preadolescence or later), and that it should only be completed in a center offering comprehensive, multidisciplinary care [171].

#### Ponderal growth

Early in life feeding difficulties may arise because of tachypnea, gastroesophageal reflux, oromotor hypotonia or some combination of these. Together with increased work of breathing (see above), failure to thrive may result. Thereafter, however, obesity is prevalent, probably far more prevalent than in the population at large [172]. Excess weight may be particularly problematic in persons with achondroplasia related to potential neurologic and orthopedic sequelae [173].

**Fig. 16:**
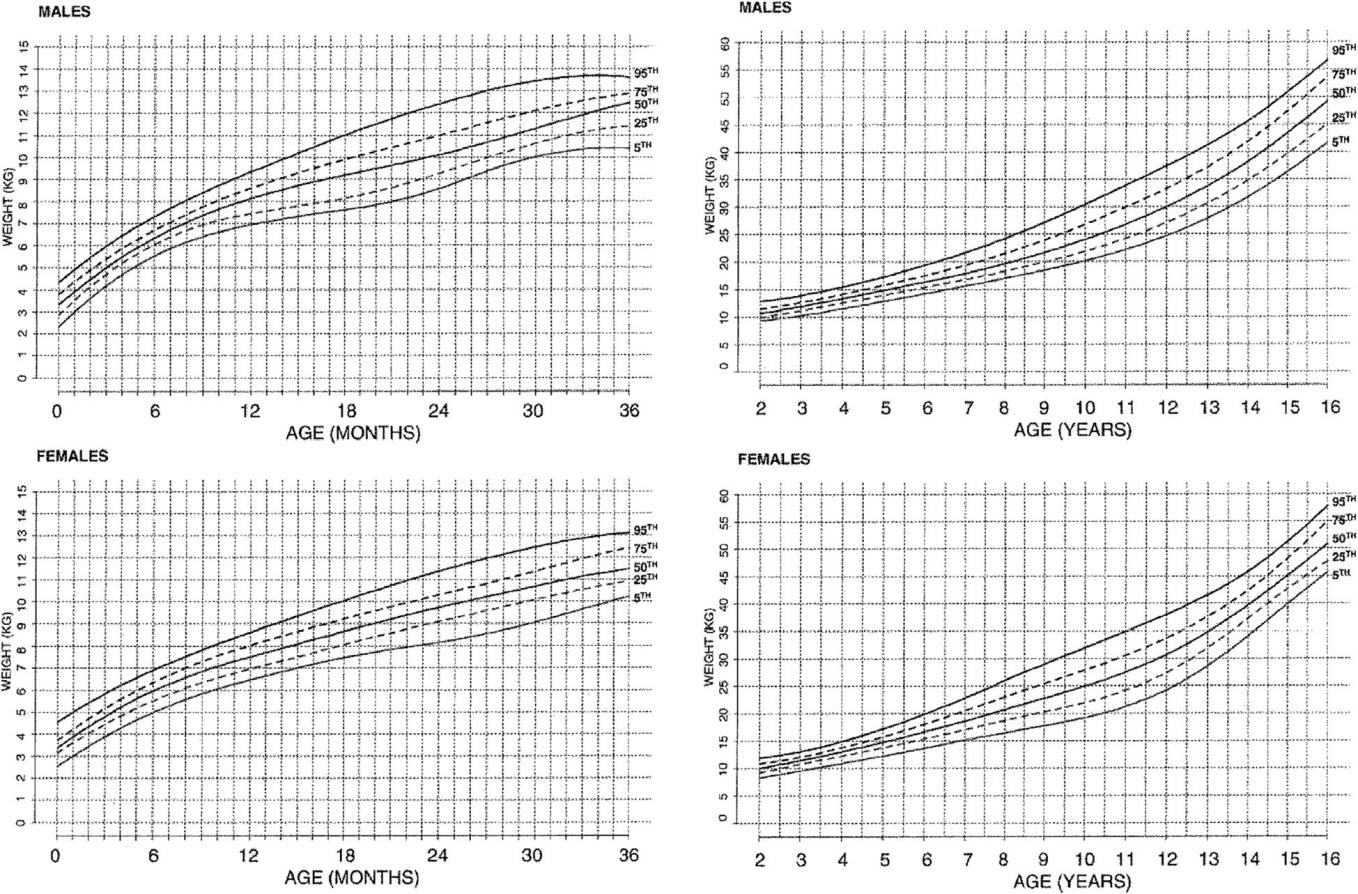
Diagnostic specific weight charts for children 0–36 months (left) and from 2 to 16 years (right). Reproduced with permission from Hoover-Fong JE et al., (2007) Weight for age charts for children with achondroplasia. Am J Med Genet A 143A:2227–2235 [173]

**Fig. 17:**
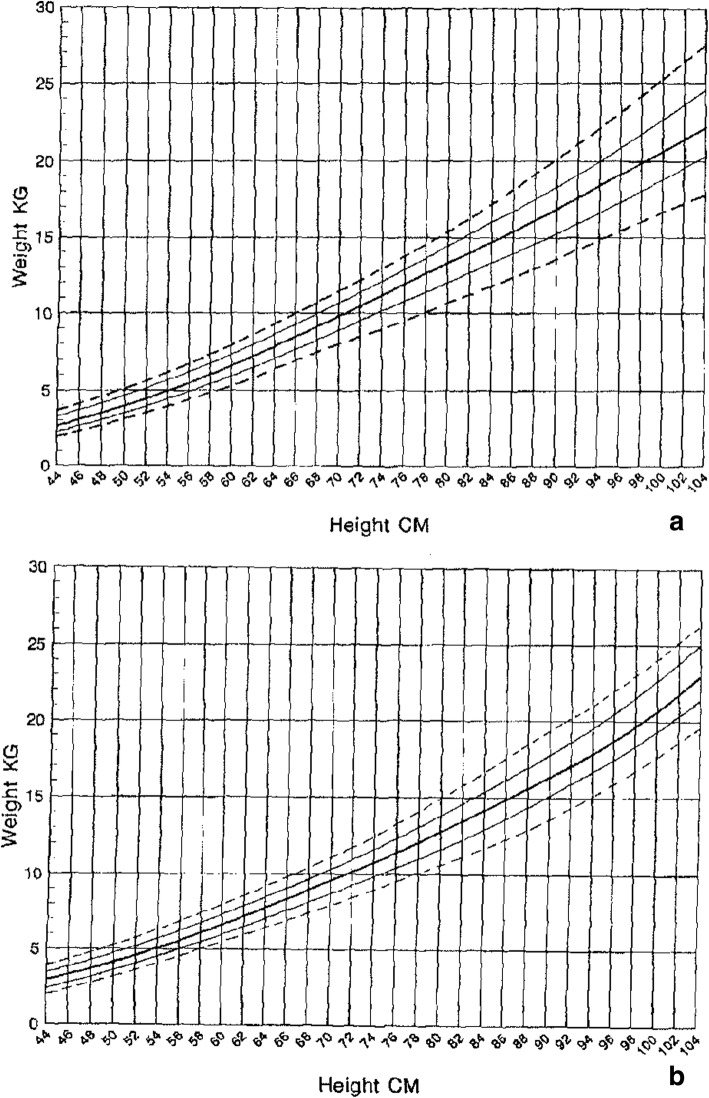
Weight by height charts for children with achondroplasia up to 104 cm in height. **a** Top is for males and **b** lower is for females. Reproduced with permission from Hunter et al. (1996) Standard weight for height curves in achondroplasia. Am J Med Genet 62:255–261 [174]

**Fig. 18:**
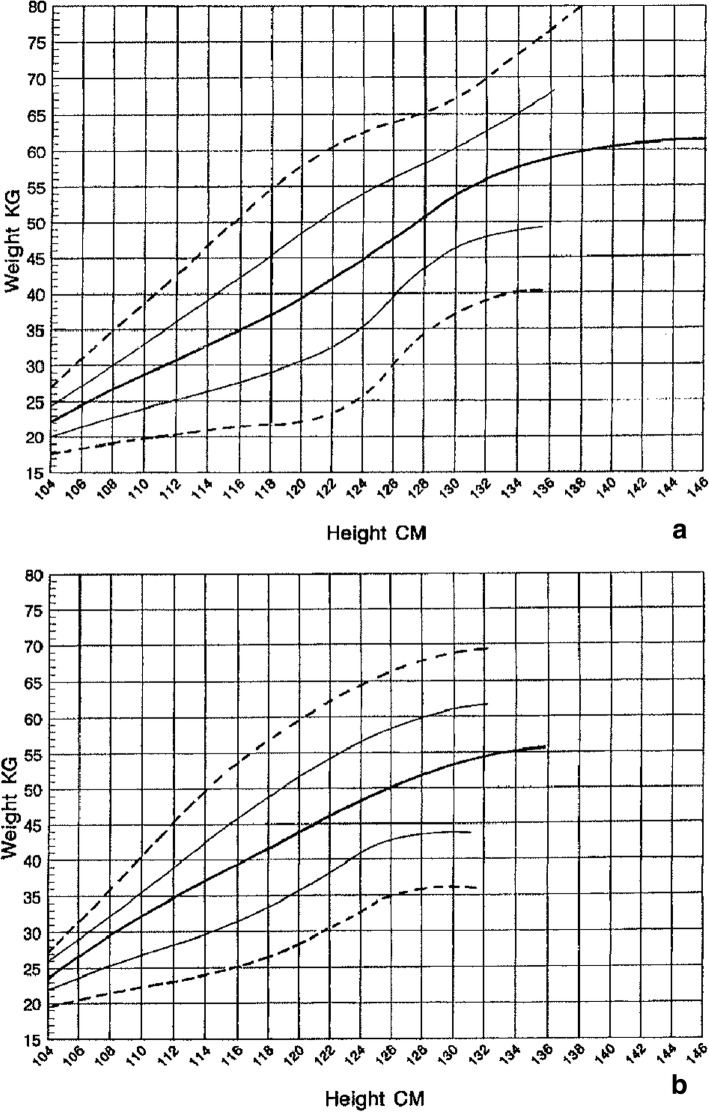
Weight by height charts for individuals with achondroplasia greater than 104 cm in height. **a** Top is for males and **b** lower is for females. Reproduced with permission from Hunter et al. (1996) Standard weight for height curves in achondroplasia. Am J Med Genet 62:255–261 [174]

Both weight for age [173] (Fig. 16) and weight-by-height [174] (Figs. 17 and 18) charts are available and should be used in all children with achondroplasia. The weight-by-height charts are helpful in caring for adults as well.

**Fig. 19:**
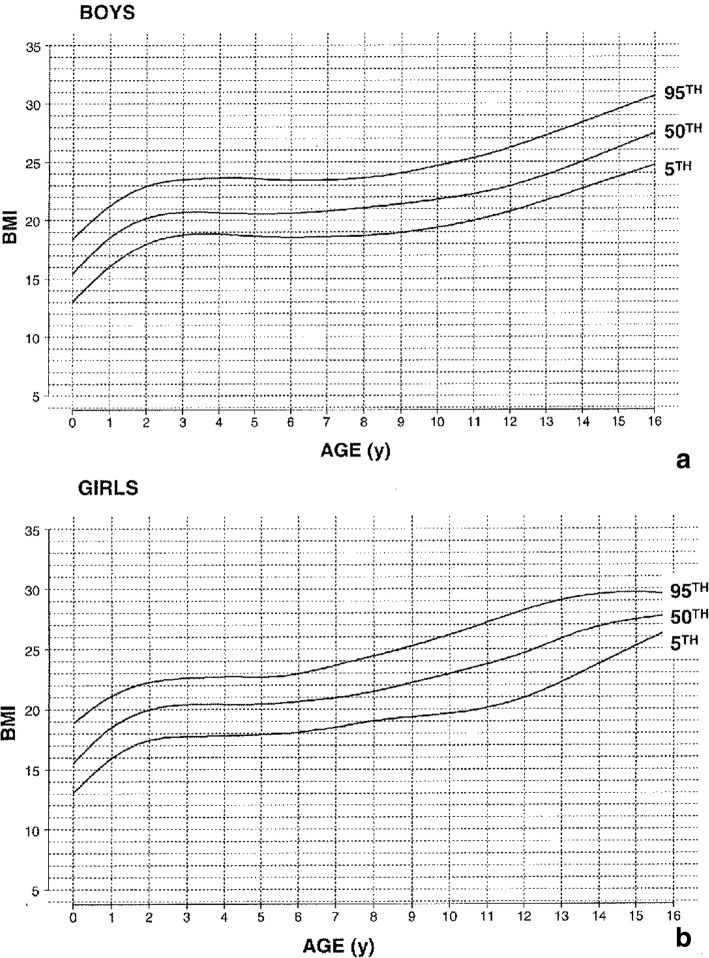
Diagnosis-specific body mass index standards for children with achondroplasia. **a** Top is for males and **b** bottom for females. Reproduced with permission from Hoover-Fong JE et al. (2008) Age-appropriate body mass index in children with achondroplasia: interpretation in relation to indexes of height. Am J Clin Nutr 88:364–371 [176]

For those who elect to use the Body Mass Index (BMI) to assess for obesity [175], note should be made that standards for average individuals will incorrectly define most individuals with achondroplasia as being obese. This arises because of the marked differences in body proportions [176]. Diagnosis specific BMI standards are now available (Fig. 19) [176].

All currently available ponderal standards are population based – they reflect what *is*, not necessarily what *should* be.

##### Interventions for obesity

Energy expenditure and caloric need appear to be less in those with achondroplasia [177]. Although typical interventions to prevent or treat excess weight are usually effective, this means that efforts at weight loss may need to be more rigorous and aggressively supported. Experience suggests that, in general, caloric need in those with achondroplasia is about 2/3 that of individuals with average stature.

Bariatric surgical procedures have been successfully carried out in obese adults with achondroplasia [178] (and personal observations).

### Development

Cognitive function is normal in most persons with achondroplasia [179, 180], although it has long been recognized that developmental delays, particularly motor delays, are common [179, 181]. Of course, cognitive issues may arise secondary to other sequelae of achondroplasia – hydrocephalus, hypoxic injury and so forth. Furthermore, untreated obstructive sleep apnea may have serious developmental consequences in children with achondroplasia [182].

The first attempt to provide standards for comparison of development in a child with achondroplasia to similarly affected peers was that of Todorov et al. [181] The resultant tool, patterned after the Denver Developmental Screening Test [183], served only as a very rough guide to expected development; this is particularly so because it was generated using temporally remote retrospective recall. Furthermore, it addressed possible *delays* in development but not *differences* in development.

**Fig. 20:**
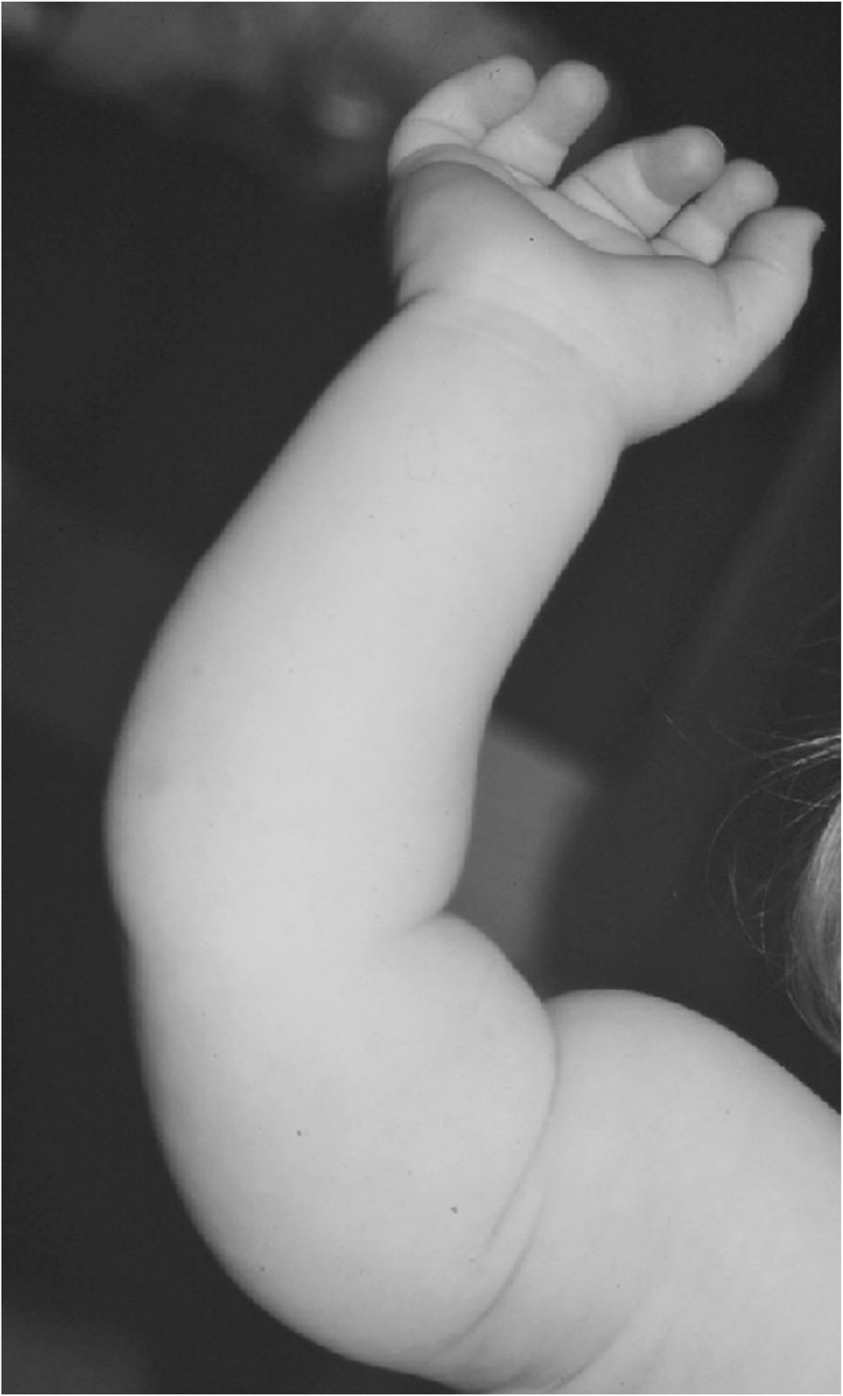
Limited elbow extension in a young child with achondroplasia

**Fig. 21:**
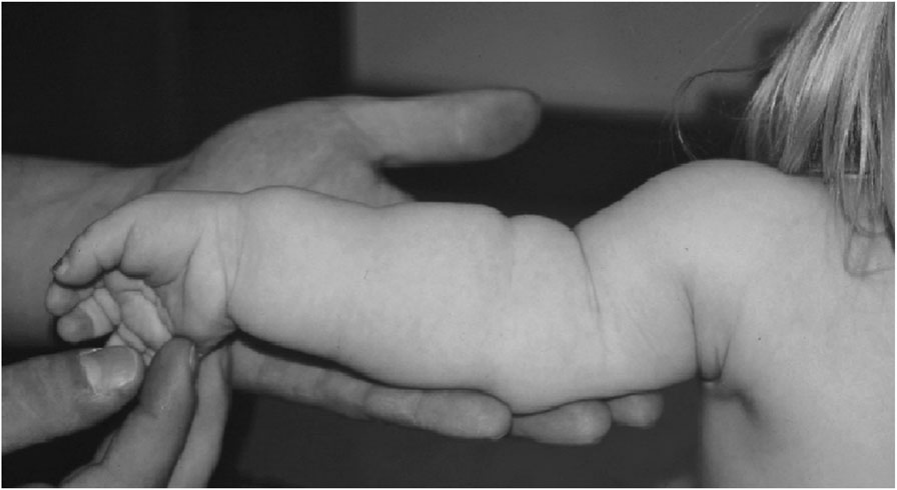
Marked rhizomelia in a child with achondroplasia

**Fig. 22:**
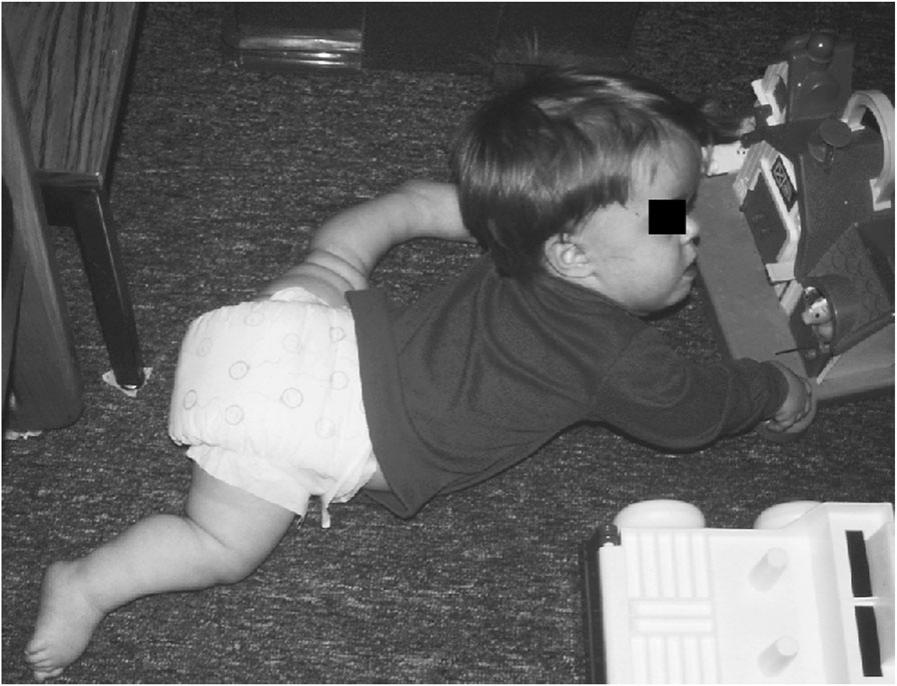
Position – remarkably, a comfortable one – illustrating the large joint hypermobility that is present in younger children with achondroplasia

**Fig. 23:**
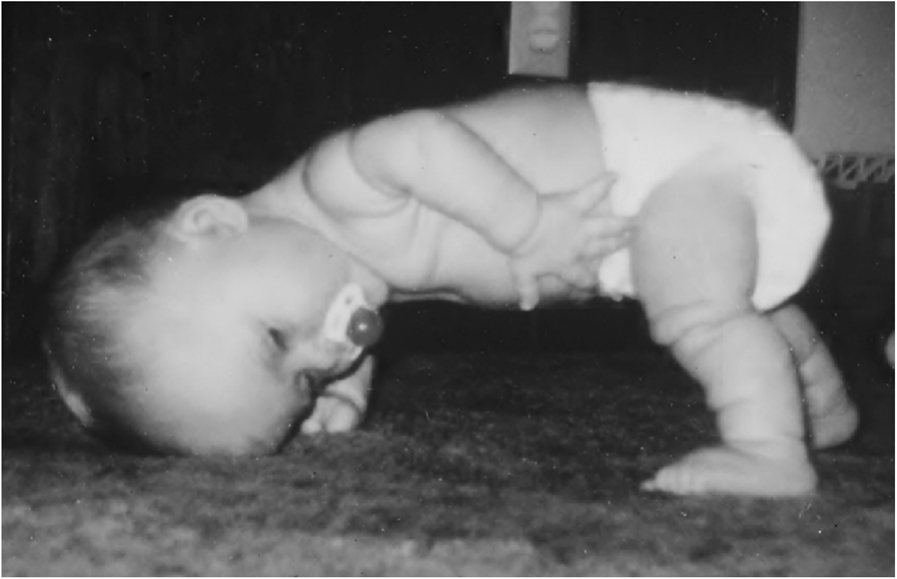
Snowplowing. As described in the text, movement is effected by pushing with the feet, sliding the head forward

**Fig. 24:**
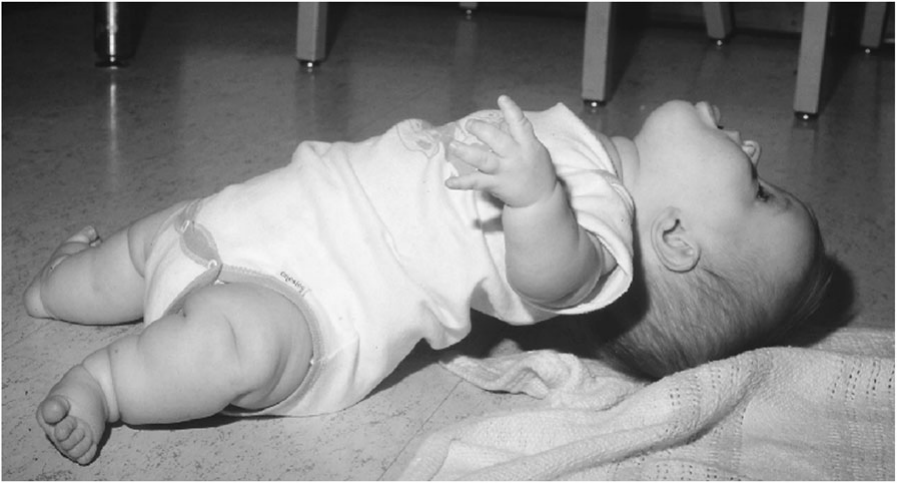
Reverse snowplowing. Here pushing with the heels propels the child who is also supported by the back of the head. Originally published in Fowler ES et al. (1997) Biophysical bases for delayed and aberrant motor development in young children with achondroplasia. J Dev Behav Pediatr 18:143–150 [184]

**Fig. 25:**
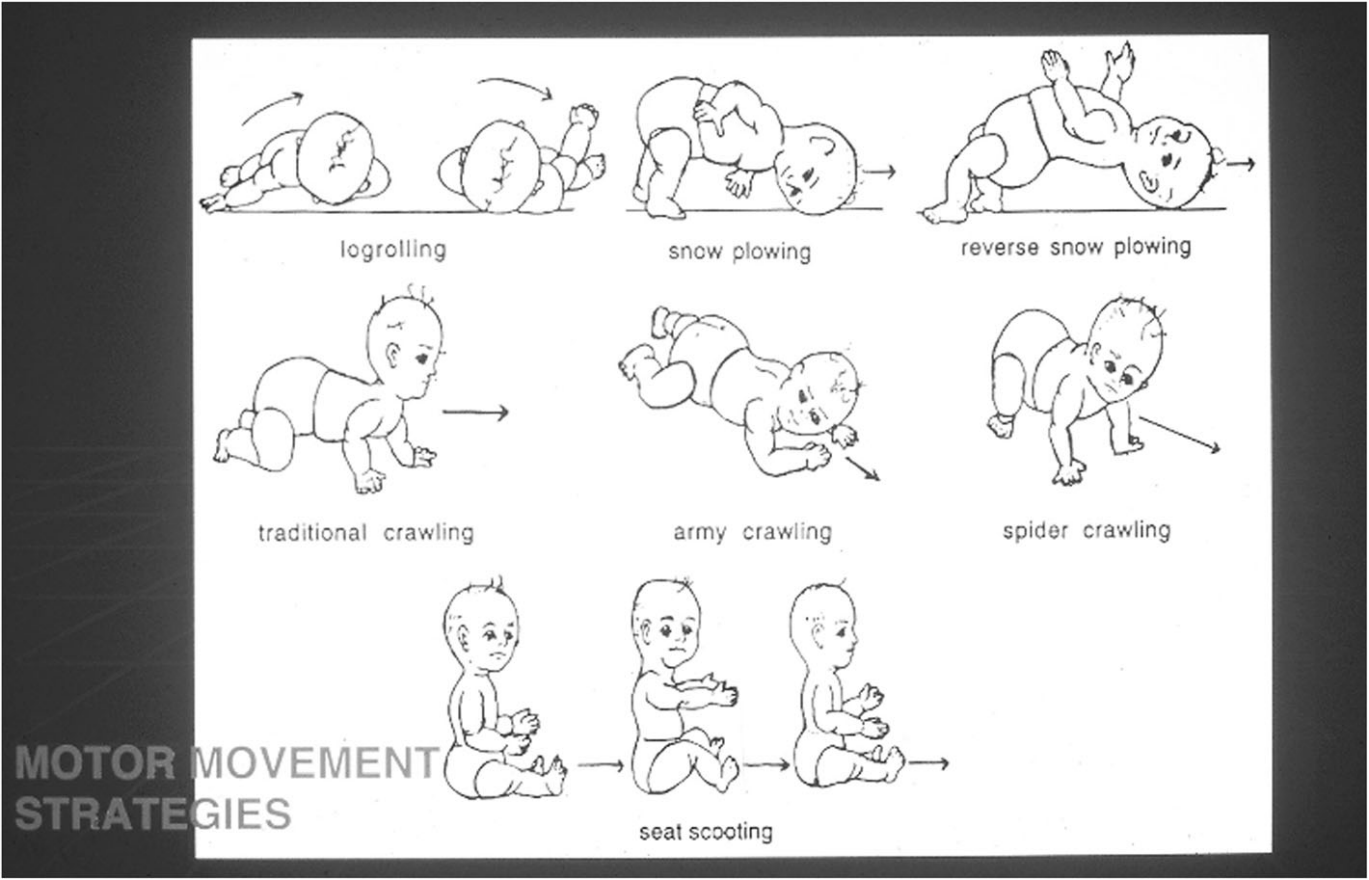
Preorthograde motor movement strategies for infants with achondroplasia. Originally published in Fowler ES et al. (1997) Biophysical bases for delayed and aberrant motor development in young children with achondroplasia. J Dev Behav Pediatr 18:143–150 [184]

Subsequent studies have emphasized the motor issues that are often present in young children with achondroplasia [179, 184]. Children with achondroplasia are not only uniformly motor delayed, but display unusual patterns of motor development [184]. A number of bioanatomic differences appear to underlie these differences – including marked rhizomelic shortening (Fig. 20) of the arms and legs, limited elbow extension (Fig. 21), generalized joint hypermobility (Fig. 22), macrocephaly and hypotonia [184]. Together such features make typical pre-orthograde movement strategies senseless for a baby with achondroplasia. Many children, instead, elect “snowplowing” (Fig. 23) (movement with support provided by the feet and forehead) or “reverse snowplowing” (Fig. 24) (support provided by the heels and the occiput). Although such strategies may elicit parental concern, in fact they should be viewed as normal and adaptive differences in children with achondroplasia. Fowler et al. [184] quantified the frequency of such strategies (Fig. 25) by parental questionnaire and demonstrated that most babies with achondroplasia seat scoot or snowplow, and that many reverse snowplow as well. The occurrence of such unique movement strategies has been confirmed in a prospective study of Ireland et al. [9] This is one of a series of important contributions regarding development in achondroplasia by Ireland and her colleagues [9, 185–187].

Gross motor delays are substantial. Median age of walking independently is around 18 [184] or 19 [9] months. Those medians hide a remarkably broad range, so that first independent walking may not occur until well after the 2nd birthday [9]. Gross motor issues may be sufficient that with increasing age they result in greater caregiver dependence [186].

**Fig. 26:**
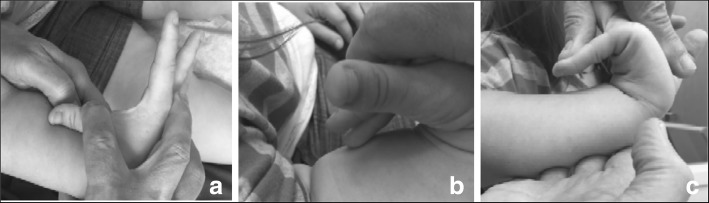
Demonstration of wrist hypermobility in a school-aged child with achondroplasia. **a** Positive thumb sign (with wrist flexion the thumb touches the wrist); **b** positive envelope sign (with wrist flexion all of the fingers can touch the wrist); and **c** positive 5th finger sign (with wrist extension the 5th finger can be brought parallel to the wrist)

**Fig. 27:**
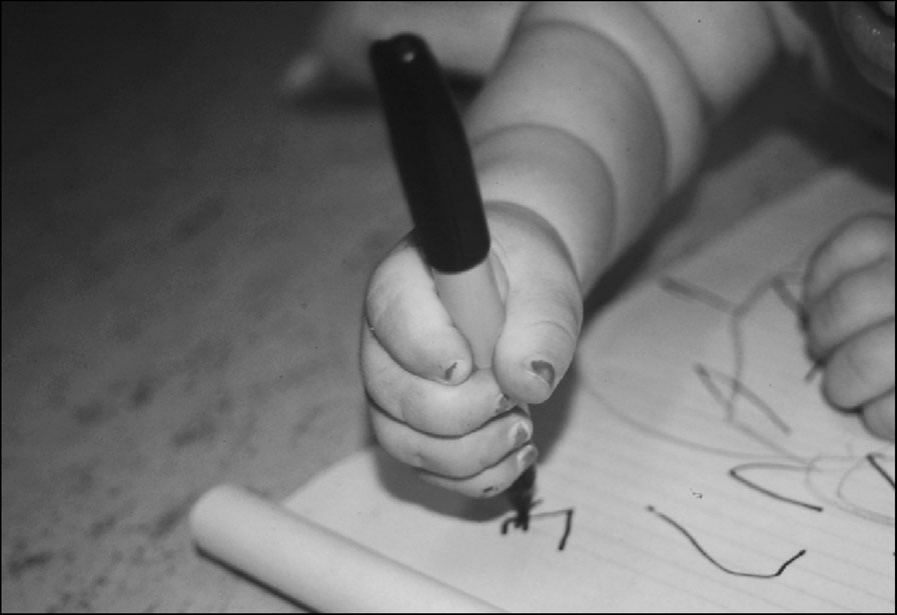
Four-finger grasp. Originally published in Fowler ES et al. (1997) Biophysical bases for delayed and aberrant motor development in young children with achondroplasia. J Dev Behav Pediatr 18:143–150 [184]

**Fig. 28:**
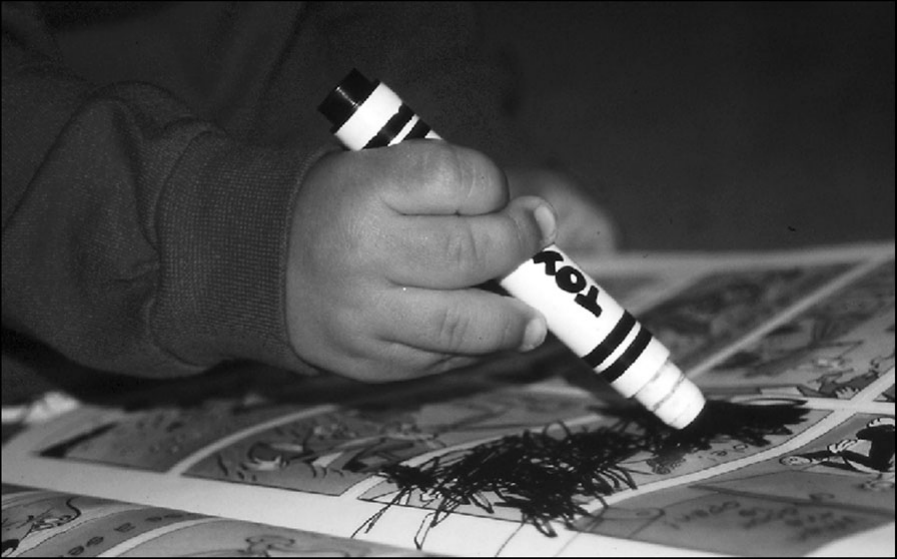
Two-finger grasp taking advantage of the trident configuration gap between the third and fourth fingers. Originally published in Fowler ES et al. (1997) Biophysical bases for delayed and aberrant motor development in young children with achondroplasia. J Dev Behav Pediatr 18:143–150 [184]

Fine motor differences also appear to have biophysical bases, including brachydactyly and trident configuration of the fingers (Fig. 7) and small joint hypermobility (Fig. 26). While fine motor issues are less marked and attainment of fine motor skills much less delayed than gross motor ones [9, 184], differences are frequently observed. For example, because of brachydactyly and hypermobility of the wrists and fingers, there often is persistence of a four-finger grasp (Fig. 27) or two-finger grasp (Fig. 28), the latter often taking advantage of the trident deformity (Fig. 7). As children get older there often are complaints of fine motor fatigability, inability to exert sufficient pressure with pencils, etc.

A larger than expected number of children with achondroplasia have language delays [188, 189]. Documented delays are most often of expressive language [9, 190]. Unrecognized persistent or fluctuating hearing loss is common in those with achondroplasia (see Ears and hearing), and may explain much of these expressive delays [188]. It also may, in part, arise from how adults interact with children with achondroplasia [9]. It may in part be related to the expression of *FGFR3* in the brain [191]. In the vast majority of children with such expressive language delays, with appropriate speech and language therapy normalization will occur by 5 or 6 years of age (personal observation).

It appears that quite infrequently, but still at a higher frequency than in the general population, children with achondroplasia may have autism spectrum disorders [190]. This possibility has not yet been adequately documented or confirmed.

**Fig. 29:**
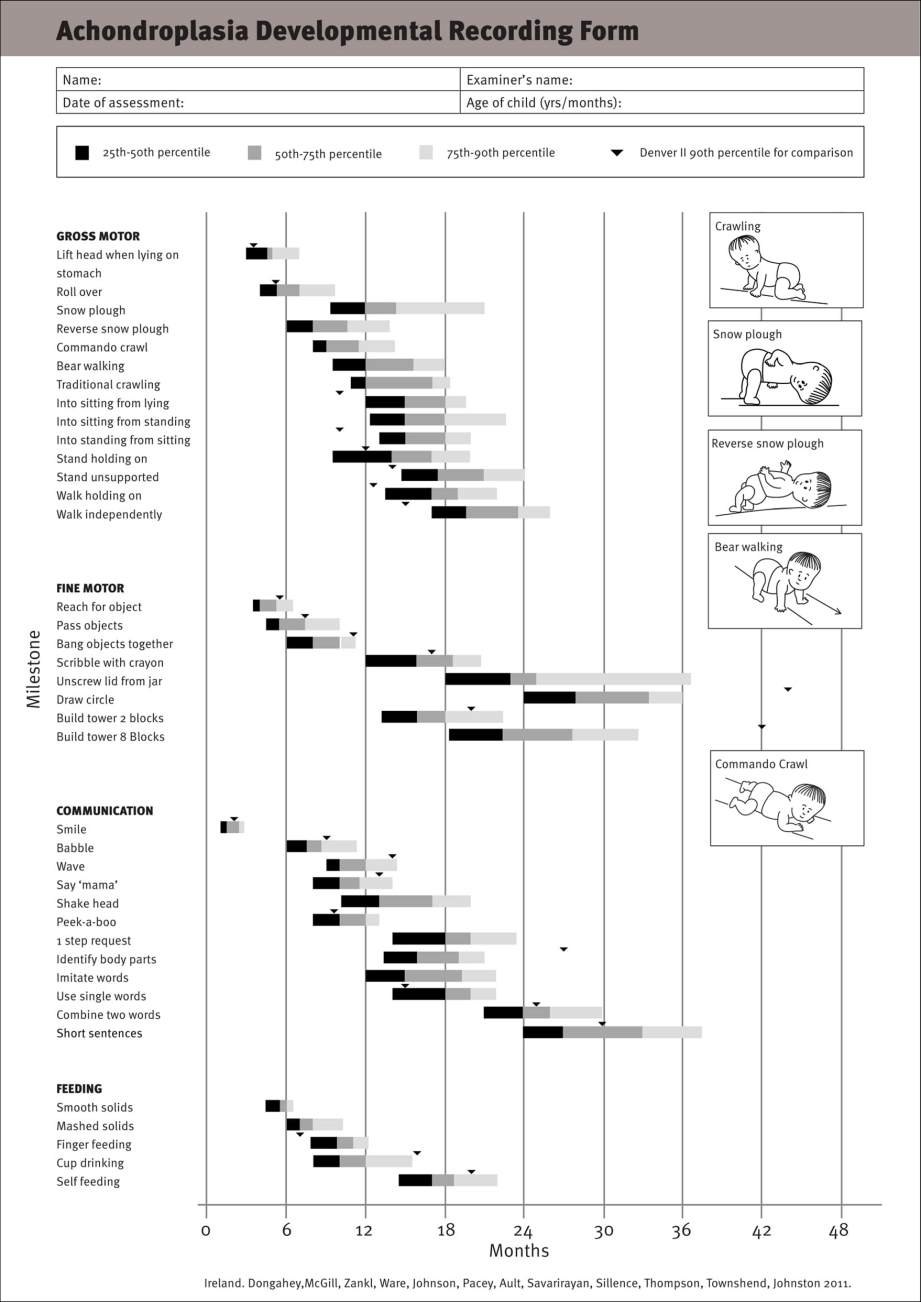
A developmental screening tool developed by Ireland et al. It is currently the best alternative for developmental screening of children with achondroplasia. Reproduced with permission from Ireland PJ et al., (2012) Development in children with achondroplasia: a prospective clinical cohort study. Dev Med Child Neurol 54:532–537 [9]

Currently the most helpful screening tool is that of Ireland et al. [9] It is replicated in Fig. 29. This, or other standards, should be used in screening every child with achondroplasia.

### Neurologic risks: hydrocephalus

Most individuals with achondroplasia are macrocephalic [61]. Large head size appears to have multiple contributing factors. Megalencephaly of mild degree is typical [192], perhaps because of direct effects of FGFR3 on brain morphogenesis [47]; typically there is both ventriculomegaly and excess extra-axial fluid [193], presumably a result of a mechanism shared with the process that sometimes results in hydrocephalus [194] – see below. It is critical to distinguish between “normal” macrocephaly with large ventricles under normal pressure and excess extra-axial fluid, and clinically significant hydrocephalus.

**Fig. 30:**
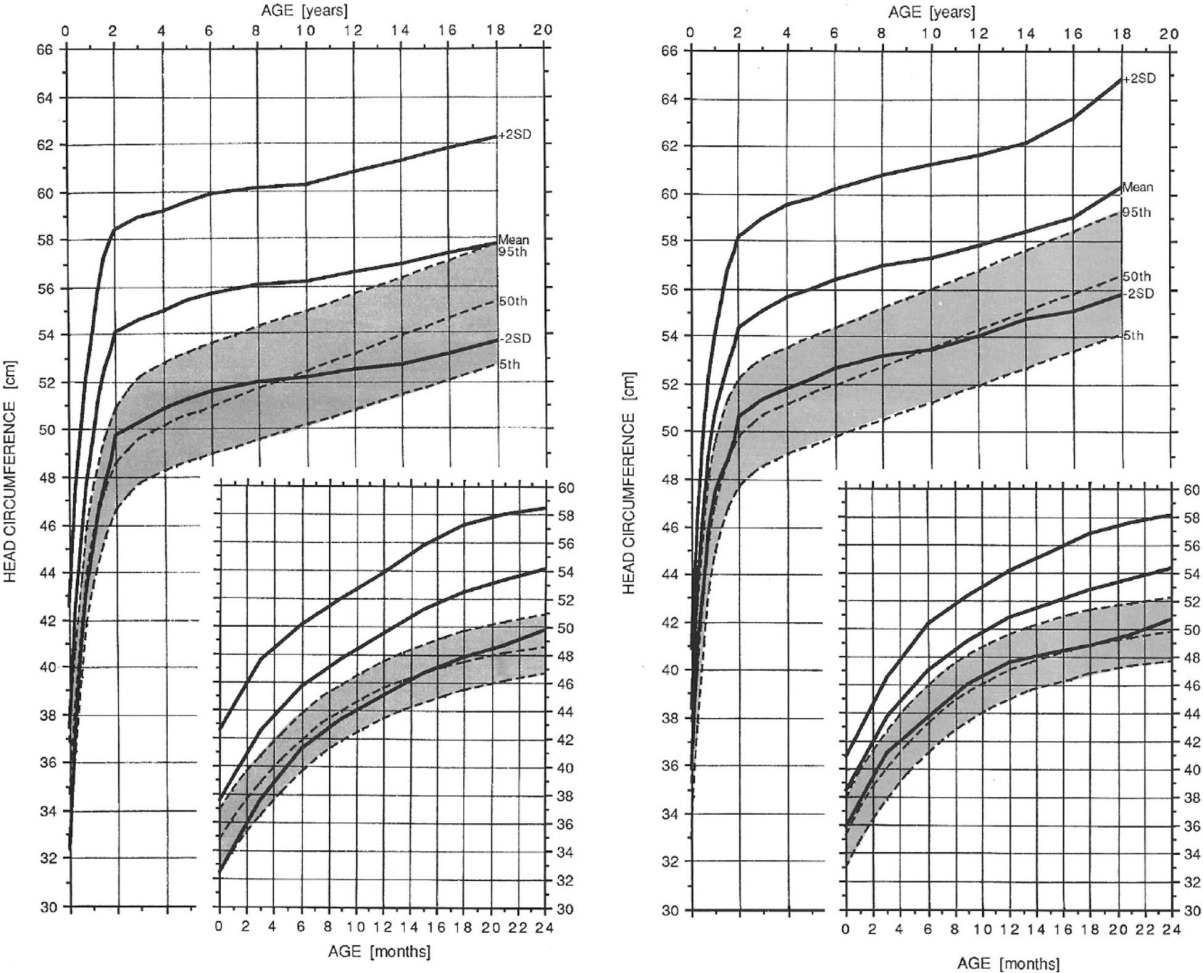
Head circumference reference standards for females (left) and males (right) with achondroplasia. Comparable measurements for average stature individuals are shaded. Reproduced with permission from Greenwood Genetics Center (1988) Growth References from Conception to Adulthood. Clinton, SC: Jacobs [156]

All children with achondroplasia should have head circumference measurements at every health care contact, with those plotted on achondroplasia specific head circumference standards [61] (Fig. 30). This should continue until at least 5–6 years of age, since there is delay of sutural maturation in achondroplasia (including persistence of the anterior fontanel until as late as 5 or 6 years [personal observation]) and, so, increased intracranial pressure can result in acceleration of head growth for far longer than in other children. Plotting head circumferences on typical standards will give the *spurious* impression of accelerating head growth with crossing of centiles.

#### Level of risk

Although Hunter et al. reported that ~ 10% of individuals in a multicenter review had a ventricular shunt placed [195], because this retrospective assessment covered many decades, it is likely this includes persons in the earlier part of the period who were shunted without what would now be considered unequivocal need. A more recent study reported an incidence of 4.3% of children with achondroplasia requiring shunting [144]. This is more in keeping with our own experience. Diagnosing those 3–5% of children who require treatment is challenging.

#### Presentations

**Fig. 31:**
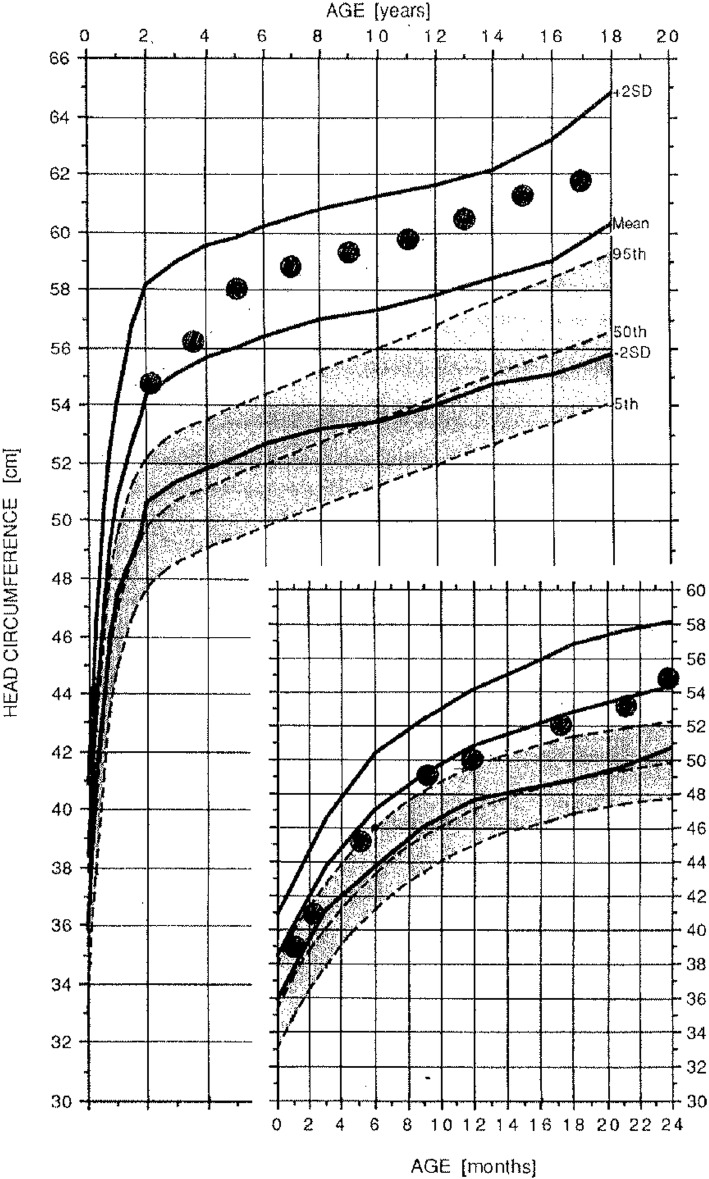
Sequential head circumference measurements in a boy with achondroplasia. Transient acceleration of head growth occurred at around 4–5 years of age (accompanied by non-specific symptoms including occasional emesis). Neuroimaging at that age did demonstrate increased ventricular size compared with imaging completed in the first year of life. There was subsequent equilibration of head growth without intervention. Now an adult, the individual is of normal intelligence and without any indicators of any harmful effects of this transient acceleration and re-equilibration

In some individuals there is transient acceleration of head growth with few or no accompanying symptoms suggestive of increased intracranial pressure. Then there may be re-equilibration, rechanneling of head growth (Fig. 31) and a benign course [196]. This suggests that in symptom free individuals a period of watchful waiting is appropriate [145], even if imaging demonstrates increasing ventriculomegaly compared with imaging obtained in early infancy.

In a few children it appears that there is intermittent, episodic increased intracranial pressure [25, 197, 198]. This may result in acute and severe symptoms, but without persistence. Whether this is present and relevant can only be assessed with intracranial pressure monitoring [198].

Rarely infants will have acute and dramatic signs and symptoms of hydrocephalus. More often, its development is more insidious, with mild and difficult to pinpoint symptoms such as lethargy, irritability, headache etc. In those instances signs become more important. Parents should be taught that a bulging and tense anterior fontanel, or increasing prominence of superficial venous patterning (see below), along with lethargy or irritability and/or emesis requires urgent assessment.

#### Mechanism

**Fig. 32:**
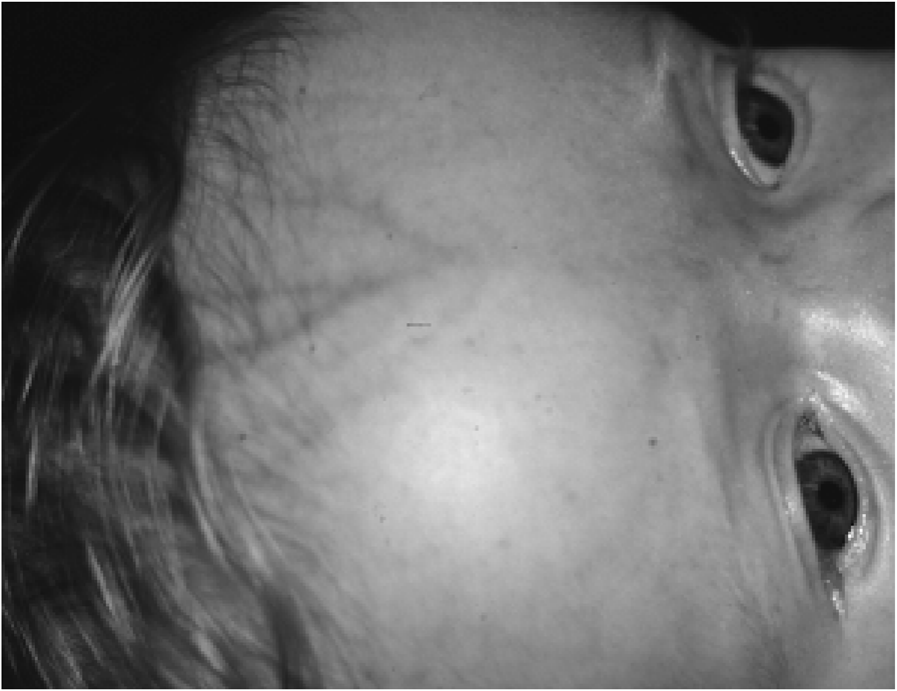
Typical superficial venous prominence in an infant with achondroplasia. This arises from increased flow through emissary veins secondary to increased resistance to flow at the jugular foramina

Unlike the obstructive hydrocephalus typically encountered, the mechanism of development of hydrocephalus in achondroplasia is thought to be distinctive. Just as the foramen magnum is of diminished size because the cranial base is endochondral bone, so, too, the jugular foramina on either side of the foramen magnum are smaller. Evidently, this can lead to partial obstruction of venous flow through them [128], which in turn results in increased intracranial venous pressure. Intracranial venous hypertension causes limitation of venous resorption of cerebrospinal fluid [193, 194]. Along with causing increasing cerebrospinal fluid accumulation and, at some critical tipping point, increased intracranial pressure, the obstruction of venous outflow at the jugular foramina causes alternative flow to become more important. The emissary veins assume that role of collateral channels [199, 200] resulting in prominence of superficial veins of the scalp and skull (Fig. 32). Sudden increase in superficial venous prominence probably is indicative of worsening outflow obstruction at the jugular foramina and increased risk that hydrocephalus is developing.

Although this is likely the most important mechanism, there is also evidence that obliteration of cerebrospinal fluid flow at the craniocervical junction may be a factor in development of hydrocephalus, too [128, 129, 201, 202]. It is not unreasonable to posit that there are two distinct processes that can give rise to increased intracranial pressure in those with achondroplasia.

#### Management

In those who clearly need treatment, ventriculoperitoneal shunting is standard [1, 4]. Surgical and post-surgical care are no different than in others without achondroplasia who require shunting.

One might think that jugular foramenotomy would be the logical approach, and this has been successfully done [203]. However, this is challenging surgery and has not supplanted use of ventriculoperitoneal shunting.

Third ventriculostomy has been carried out in a few children with achondroplasia, apparently successfully [196, 204]. Good outcomes with this procedure (which should not be effective if intracranial venous hypertension rather than obstruction is the mechanism of hydrocephalus) could mean either that intervention was not really needed, or that a second, obstructive mechanism such as flow restriction at the craniocervical junction may sometimes be important [129].

If there are, in fact, two distinct mechanisms giving rise to hydrocephalus in those with achondroplasia, distinguishing which mechanism or mechanisms is operative in an individual is not straightforward.

Distinguishing which intervention is most effective is, in theory, uncomplicated. Prospective randomization to two alternative treatment arms of patients presenting with signs and symptoms of hydrocephalus is easy to envisage. However, in practice such a trial is virtually impossible. Over the course of 30 years, the largest specialty clinics assess around 400 unique individuals with achondroplasia [10]. Each such clinic, then, would identify only around 0.7 eligible patients ([400 ÷ 30] × .05) per year. Not only would a collaborative venture be needed, but even so only after many years would sufficient numbers be accumulated.

#### Subdural hematomas

The subarachnoid spaces are enlarged in most children with achondroplasia. Because of this increased extra-axial fluid, the bridging vessels (which may also be distended because of obstruction at the jugular foramina) may be especially susceptible to shearing trauma. Several instances of subdural hematoma formation in children with achondroplasia [205] (and personal observation) following minimal trauma probably arose because of this increased susceptibility. Such an occurrence should not be interpreted as necessarily implying that nonaccidental trauma has occurred.

### Neurologic risks: the craniocervical junction after infancy

Although the risk for unexpected death secondary to craniocervical constriction nearly disappears after the first year of so of life [8, 107, 113], the foramen magnum remains small. This results in a residual risk that an individual may experience spinal cord damage, either insidiously or acutely. That high cervical myelopathy develops in some individuals with achondroplasia has been recognized for a long time [115, 206, 207]. Such cervical myelopathy can arise at any age [129], although it seems more frequent in childhood – perhaps because of continued, albeit slow, growth of the chondrocranium with age [122] or perhaps because precipitant trauma is more likely in children. No estimate is available for level of risk.

In young children clinical features that may suggest cervical myelopathy include long persisting hypotonia, asymmetric resistance and strength, asymmetric reflexes, ankle clonus, or upgoing response to Babinski stimulation [8]. After a child begins to ambulate, note may be made of classic features of cervical myelopathy [208] including orthograde fatigability, decreased endurance, apparent sudden, transient pain in the arms or legs, decreased fine motor function or changes in bowel or bladder continence. Alternatively, a child may present with acute, severe myelopathy secondary to injury [209] which requires urgent neurosurgical involvement.

Given risks related to craniocervical constriction, all individuals with achondroplasia must be managed as having a small foramen magnum and increased risk for trauma-based cord compression. It is crucial that parents be counseled about physical activities and sports activities that create unacceptable risk [1, 4]. Activities that should be prohibited, or at least strongly discouraged, include: trampoline use; vaulting in gymnastics; diving off of diving boards; sparring in martial arts; (American) football; rugby; downhill skiing; heading in soccer, etc. On the other hand, many physical activities are reasonably safe and can be encouraged, such as: swimming; golf; tennis; basketball; soccer (except for heading and only at younger ages, since competitive soccer becomes much more risky in older children); baseball; softball. If parents understand that what one is trying to do is limit activities that cause substantial risk for forceful head and neck injury, they usually can assess whether a particular endeavor is unsafe. Note that because what creates risk is violent neck movement, helmet use is not preventative.

In younger children, continuing rear facing of the car seat for as long as tolerated is probably prudent. Safety precautions such as gated stairways are also especially important.

Some healthy and asymptomatic children when examined carefully have “leftovers” of presumed temporally distant and minor cervical cord injury, including for example, ankle clonus, upgoing response to Babinski stimulation, etc. (personal observation). The only relevance of these findings probably is to document in medical records and to explain to the parents their presence, since they may be cause for undue concern in some circumstances. There are reports of MRI T-2 signal abnormality at the craniocervical junction in as many as 40% of apparently asymptomatic adults with achondroplasia [140, 210, 211]. These were often associated with thinning of the cord in the same region [211]. Although the origin of these lesions is unknown, it is conceivable that they reflect gliosis arising secondary to such temporally distant, minor cord injury [211], which injury could be either direct compression or of vascular compressive origin. Because careful neurologic examinations are not reported, postulating a relationship between these lesions and the clinically demonstrable “leftovers” remains speculative, however attractive.

In contrast with some other bone dysplasias, atlanto-axial instability is exceedingly rare in achondroplasia with only a handful of cases reported in the literature [212, 213]. It is so infrequently a concern that it probably does not need to be evaluated in any routine circumstance.

### Neurologic risks: miscellaneous

#### Seizures

Although far more common in those with hypochondroplasia [77], seizures occasionally arise in those with achondroplasia [214, 215]. In fact, paroxysmal events with apnea in infants with achondroplasia may arise from a variety of causes: secondary to abnormalities at the craniocervical junction and consequent abnormality of central respiratory control; from primary seizures; from airway obstruction related to macrocephaly and hypotonia (e.g. when in a car seat); or from gastroesophageal reflux. Particularly in infancy, distinguishing primary apnea from seizure-precipitated apneic events may be challenging.

#### Temporal lobe abnormality

Temporal lobe dysgenesis is common in individuals with hypochondroplasia secondary to *FGFR3* mutations [83, 84]. Given that achondroplasia and hypochondroplasia belong to the same family of bone dysplasias [52], it would not be surprising to identify similar dysgenesis in occasional individuals with achondroplasia. In fact, that was recently reported by Manikkam et al. [215] In neither disorder has the frequency of temporal lobe structural abnormalities been determined. Nor has the frequency with which temporal lobe dysgenesis results in seizures, or whether temporal lobe abnormalities are a marker for more severe central nervous system consequences of *FGFR3* been determined.

### Obstructive apnea

Both central apnea and restrictive breathing problems, which for the most part are exclusively issues in young infants, have already been addressed. In addition, children and adults with achondroplasia have an exceedingly high frequency of obstructive sleep apnea [153, 216, 217].

Estimating the frequency with which people with achondroplasia have sleep apnea is challenging. Ranges (in all age groups) have been from 10 to 87% [217, 218]. Most series suffer from inadequate sample size and/or ascertainment and referral biases [219]. Nonetheless, in published sequential series, most find that obstructive apnea of clinical significance arises in around 1/3 of all individuals (combining all ages) with achondroplasia (e.g., 38% in Sisk et al. [218]; 34% in Afsharpaiman et al. [219]; 32% in Collins & Choi [141]).

#### Clinical presentation

Obstructive sleep apnea may present at any age. In fact, there is a remarkably high rate of obstruction even in children less than 2 years of age [219]. Not surprisingly, however, there is a dramatic increase as physiologic hypertrophy of the lymphatic ring – and particularly the adenoids – arises between around 2 and 10 years of age.

Screening for possible obstructive apnea is challenging [220]. Nonetheless, either a parent (in children) or a sleep partner (in adults) should be taught the features that suggest that clinically significant increased upper airway resistance is present. In children with achondroplasia, soft and regular snoring occurs in around 95%, and so is not a valuable marker; this probably simply reflects air turbulence associated with anatomically small airways. Likewise, most infants with achondroplasia perspire profusely and this is not indicative of any medical issue. Features of significance in children include: neck hyperextension; loud and irregular snoring; glottal stops; observed apnea; deep, compensatory sighs; self-arousals; secondary enuresis; night-time emesis; morning headaches [218] (and personal observations). Of these, Sisk et al. [218] noted that glottal stops and observed apnea were the most predictive of finding clinically significant abnormalities by polysomnography. Daytime features in the very young are difficult to discern, but may include increasing sleep duration per 24 h period. In older children, there may be change in school performance or changes in behavior, including new onset of distractibility and poor attention [221].

In adults, there are daytime behavioral scales that can be used to query about symptomatic apnea [222]. Results using such a scale along with the sleep partner’s description of breathing pattern in sleep can help guide whether polysomnography should be done.

#### Consequences of untreated obstructive apnea

Parents need to be assured that obstructive apnea is almost never an *acutely* life threatening problem, but rather has long term effects that must be mitigated. Consequences of sleep apnea are not markedly different in individuals with achondroplasia compared with the general population. As mentioned, in children there may be negative learning and behavioral consequences [221, 223]. Because pulses of growth hormone secretion occur during sleep [224], sleep disruption can negatively affect growth, independent of the primary diagnosis of achondroplasia.

In adults daytime symptoms arising from poor sleep quality at night markedly increase the risk of various kinds of accidents [225].

Physiologic consequences can arise at any age. Particularly concerning are the cardiovascular consequences of long term sleep apnea [226], which are observable in affected individuals with achondroplasia [152]. Not only may obstruction resulting in recurrent and prolonged desaturations result in pulmonary hypertension and eventual cor pulmonale, but it may be a critical contributor to hypertension risk [227].

#### Mechanism

In children, a number of factors conspire to make obstructive apnea far more likely. In everyone there is physiologic decreased muscular tone in sleep [228] resulting, effectively, in smaller airway size. In children with achondroplasia there is hypoplasia of the midface with consequent diminution of anatomical airway size [152, 229, 230]. The anatomy of the face is predictive of likelihood of obstructive apnea in average children [231] and in children with achondroplasia, too [232] – the flatter and retruded the midface, the more likely that sleep apnea may develop. After around age 2 years there is physiologic hypertrophy of the lymphatic ring [233]. These factors are likely the major contributors to pathogenesis of obstructive apnea in most children with achondroplasia.

Other factors may play a role in some. Centrally mediated obstruction (as well as decreased respiratory effort) may arise because of cranial base abnormality [119, 142]. Gastroesophageal reflux is sometimes a critical contributor [119]. Lower airway malacia (tracheobronchomalacia) has recently been recognized to be of substantial frequency in achondroplasia and does complicate the management of other factors resulting in obstruction [234].

In adults, midfacial abnormality persists, while lymphatic obstruction is usually not significant. The most critical additional factor in many adolescents and adults is the onset of obesity, a feature strongly related to sleep apnea [235].

#### Assessment

The first steps in assessment is to elicit observational history regarding all of the features discussed above under Clinical presentation. General clinical assessment should include evaluation of severity of midface hypoplasia, degree of tonsillar hypertrophy and evidence for nares patency. In addition, contributing factors not unique to achondroplasia such as allergic rhinitis need to be ruled out.

If concern is present, two approaches can be considered. Polysomnography will objectively document the presence of and severity of obstructive apnea and disordered breathing, and is often the first elected investigation [4, 218, 219]. In children with severe and unequivocal historical symptoms, alternatively one might choose to have otolaryngologic evaluation including nasopharyngoscopy [236] completed. The latter approach, however, while allowing more rapid initiation of intervention if needed, does not provide objective data against which post-treatment studies can be compared.

When serious obstructive abnormalities are demonstrated by polysomnography, then referral should be made to a pediatric otolaryngologist for evaluation.

#### Management

Stepwise management of obstruction in children typically begins with adenoidectomy with or without tonsillectomy. Although one would expect that adenoidectomy along would usually suffice, outcome appears to be better in those who undergo tonsillectomy as well [218]. Children with achondroplasia may have an increased risk for post-operative complications [218] and probably should be hospitalized overnight following any procedure requiring intubation. While a majority will show marked improvement after surgical intervention [218] nevertheless polysomnography should be completed a few weeks after surgical intervention, since in many individuals obstruction persists at a level requiring additional treatment [216]. In those in whom additional treatment is needed, step two is the use of positive airway pressure (cpap, bipap). Positive airway pressure treatment is effective in those with achondroplasia [219, 229], including in very young children (personal observation). In a large majority, those interventions are sufficient to correct the obstructive apnea and prevent sequelae.

Rarely additional treatment may be needed. Uvulopharyngopalatoplasty [237] has occasionally been done, but the numbers are so small that benefit is difficult to assess. Rare individuals may require temporary tracheostomy (personal observation), although this is more likely to be needed for restrictive disease in infancy than because of inability to otherwise treat obstructive apnea. Occasionally surgery has been done to correct the midfacial hypoplasia, when severe, by either midface advancement [238] or distraction [239, 240].

In adults, most often positive airway pressure treatment is the primary and most important intervention. If appropriate, it needs to be accompanied by efforts at weight loss. The role of surgery in adults with obstructive apnea is unclear.

### Ears and hearing

Middle ear dysfunction is exceedingly common in both children and adults with achondroplasia. This presumably is so because of poor functioning of abnormally oriented Eustachian tubes, which abnormality, in turn, arises because of aberrant growth of the chondrocranium [241].

#### Frequency

A number of studies have assessed middle ear function and hearing in achondroplasia. All are limited, because of sample size (e.g. Glass et al. [242]; Shohat et al. [243]; Collins et al. [141]), ascertainment bias (e.g. Glass et al. [242]; Collins et al. [141]), incomplete documentation (e.g. Hunter et al. [195]), or self-referral bias (e.g. Tunkel et al. [244]). A well-designed, prospective study is very much needed. Nonetheless, currently available information provides a reasonably clear picture of middle ear issue5s in achondroplasia.

Middle ear dysfunction arises in 50–70% of individuals with achondroplasia [141, 242]. In turn, this results in approximately 50% of individuals having pressure equalizing tubes placed at some point [4, 141, 245].

Estimates of the frequency of hearing loss range from 38 to 60% in the achondroplastic population overall [195, 242, 244, 246]. In a large, cross-sectional study of a convenience sample, Tunkel et al. [244] demonstrated that 40% of the tested population failed hearing screening (even with relaxed criteria). This issue is not solely one of childhood [244], although greatest concern is appropriately centered on the period of language acquisition in early childhood, during which hearing loss can be a major factor contributing to speech and language delays.

#### Assessment and management

A high level of suspicion of possible hearing loss is appropriate at all ages. Formal behavioral hearing assessment and tympanometry should be completed by around 1 year of age [1]. These should be repeated at least yearly at least until school age [1, 244].

Because medical management of middle ear dysfunction is usually ineffectual [4], pressure equalizing tube placement is usually appropriate. Once need is demonstrated, tubes will usually be needed at least until 7–8 years of age [4].

There is an increase in the occurrence of jugular bulb dehiscence into the middle ear space [247, 248] (again because of abnormality of the chondrocranium). This can sometimes causes persistent, unilateral hearing loss [247]. Occasionally is can cause unexpected, brisk bleeding from myringotomy if not recognized before surgery [247].

When hearing loss is documented in childhood, standard approaches to habilitation are appropriate. These include parental awareness, preferential seating in school, use of environmental amplification if needed, etc. In only a few children are hearing aids warranted. However, note should be made of the remarkably high frequency of hearing loss in adults and, as well, the remarkably low frequency of hearing aid use in them [244]. It is likely that many adults would benefit from amplification.

### Orthopedic concerns: kyphosis

**Fig. 33:**
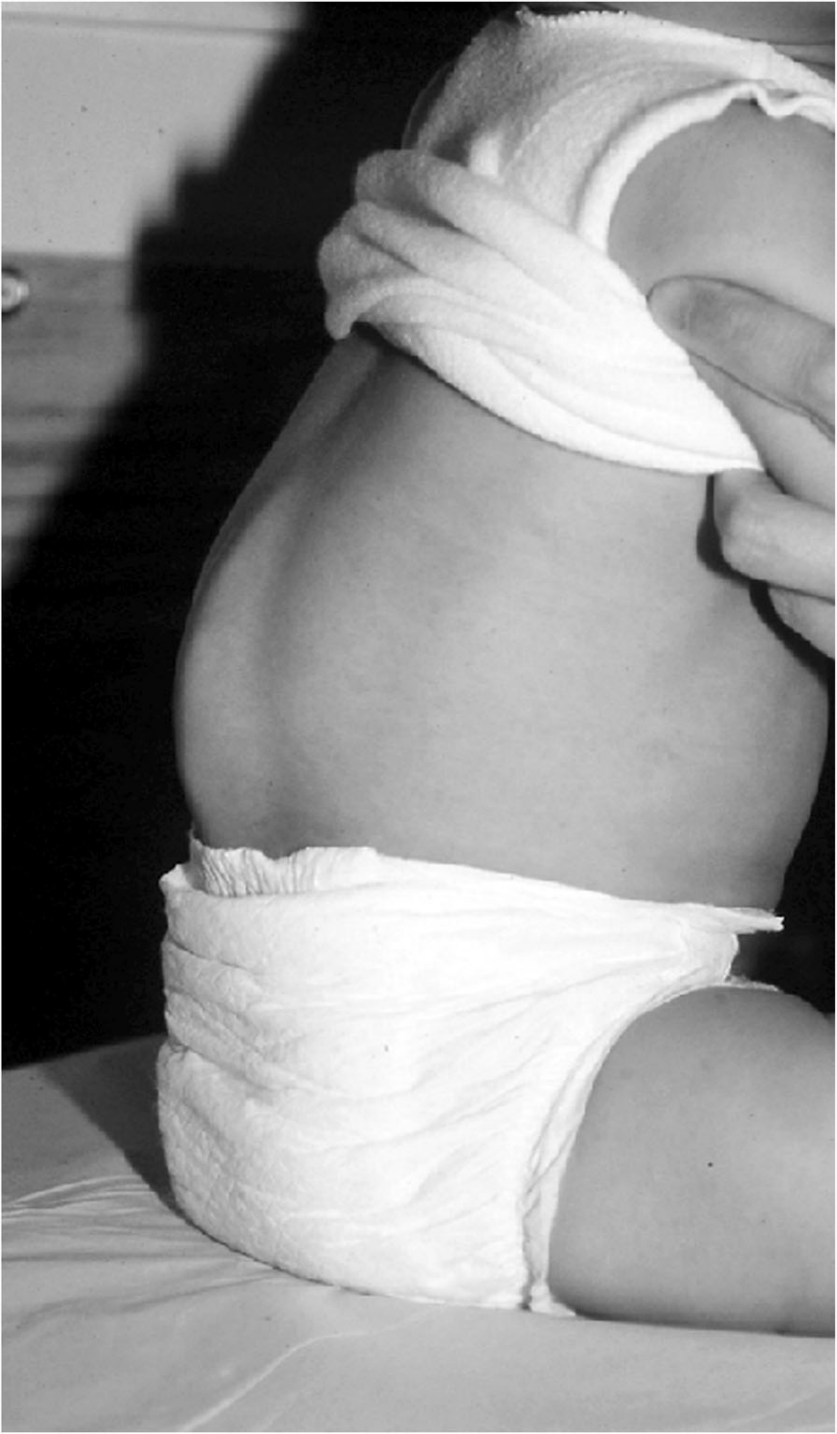
Typical flexible kyphosis seen in infants and young children with achondroplasia

**Fig. 34:**
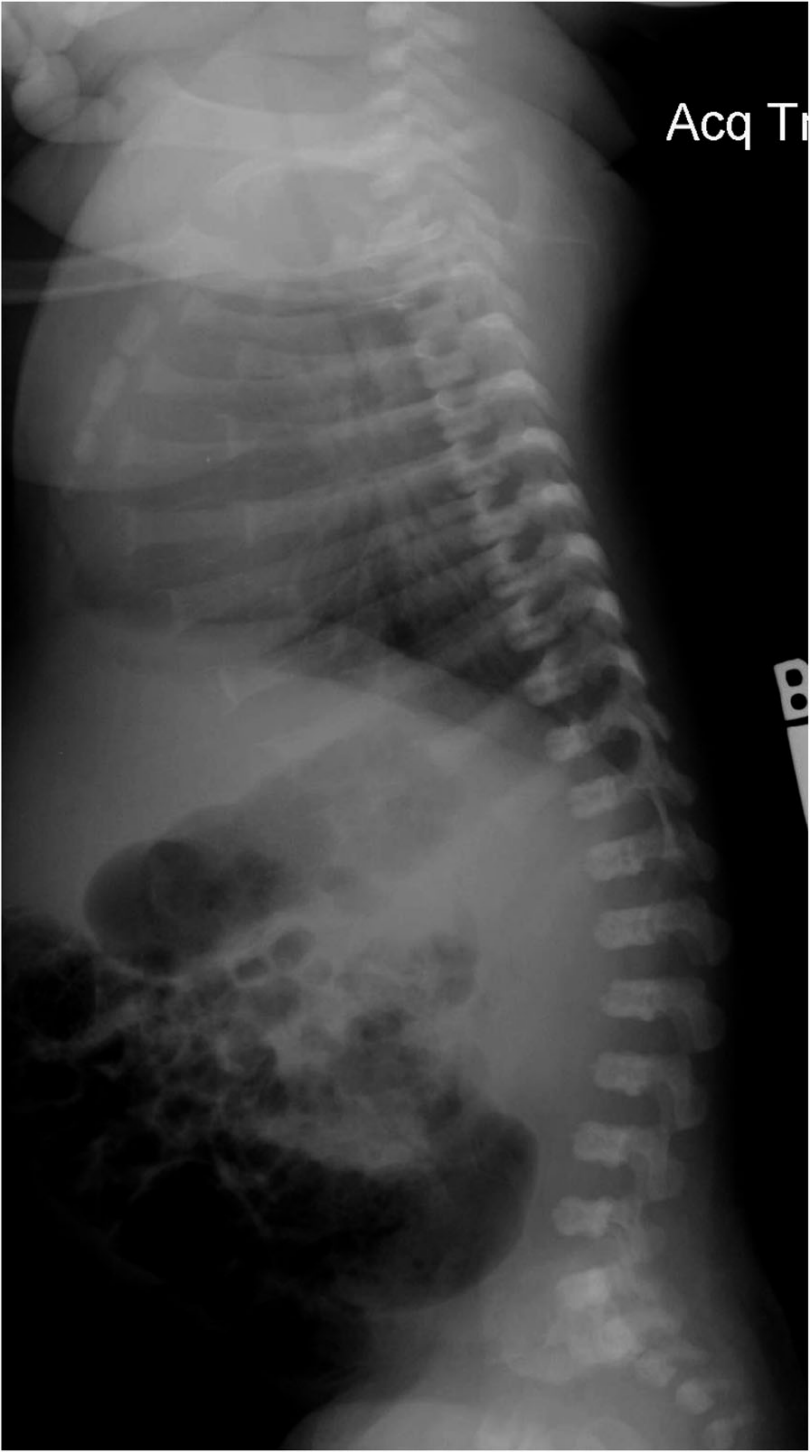
Lateral radiograph of the spine of an infant with achondroplasia. While there is obvious kyphosis, there are no secondary changes of the vertebral bodies at the apex of the curve

**Fig. 35:**
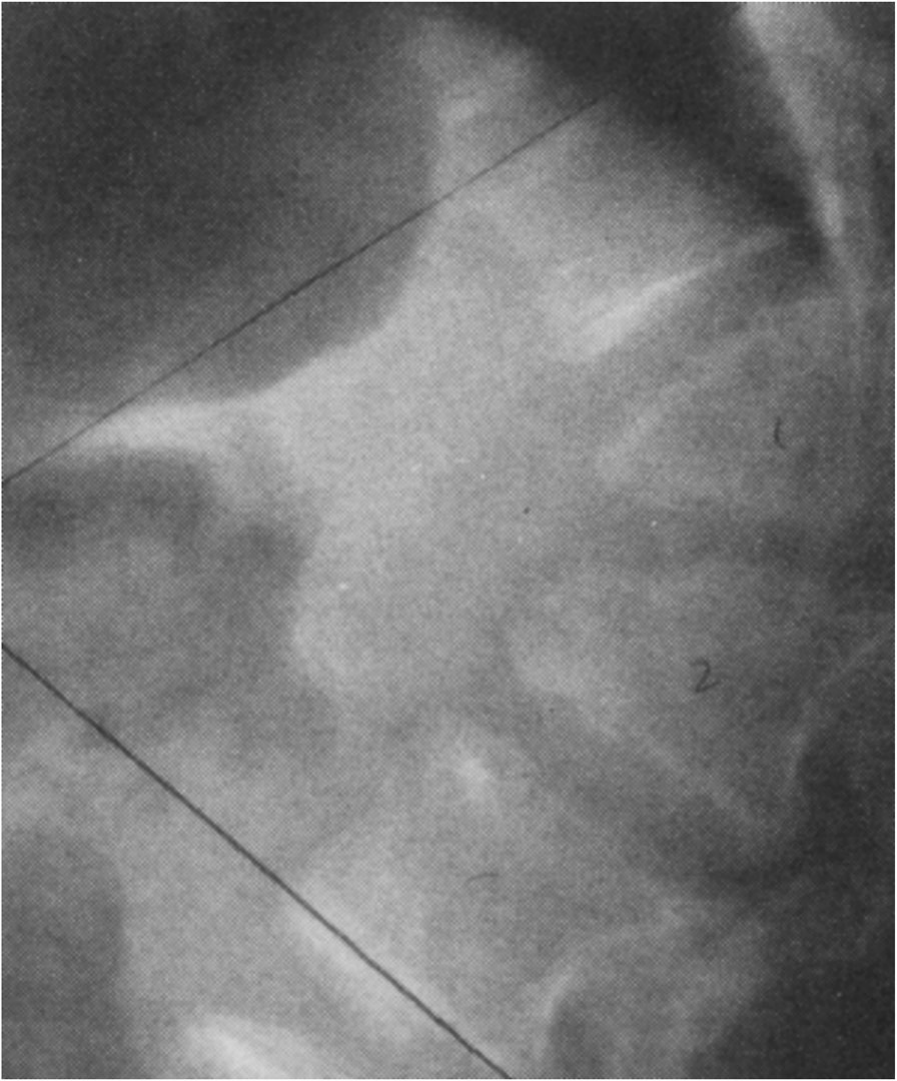
Severe, fixed angular kyphosis of the type that can be prevented by appropriate counseling and intervention in early childhood. Originally published in Pauli RM et al. (1997) Prevention of fixed, angular kyphosis in achondroplasia. J Pediatr Orthop 17:726–733 [64]

That most infants with achondroplasia develop a transient kyphosis has been recognized for nearly a century [249–251]. In fact, nearly every infant with achondroplasia under a year of age has a kyphosis at the thoracolumbar junction [64, 252] (Fig. 33). This is a non-congenital deformity unassociated with any primary structural defect of the vertebrae (Fig. 34). It usually becomes more obvious with the onset of sitting. In most it spontaneously resolves with the onset of orthograde function (standing and walking) [64, 252, 253], but 10–15% of adults have a fixed, angular kyphosis with marked secondary deformity of one or more vertebrae [252] (Fig. 35). Such wedging results in high risk for significant neurologic consequences: about ½ of adolescents and adults so affected have problems such as weakness, paralysis, bladder or bowel incontinence, etc. [252, 254, 255] These problems arise because of draping or tethering of the cord, so that with continued growth the normal “ascent” of the cord is prevented because it is now fixed at two sites (the medulla and the kyphotic apex) and with that stretch, damage to the cord results [64, 256].

**Fig. 36:**
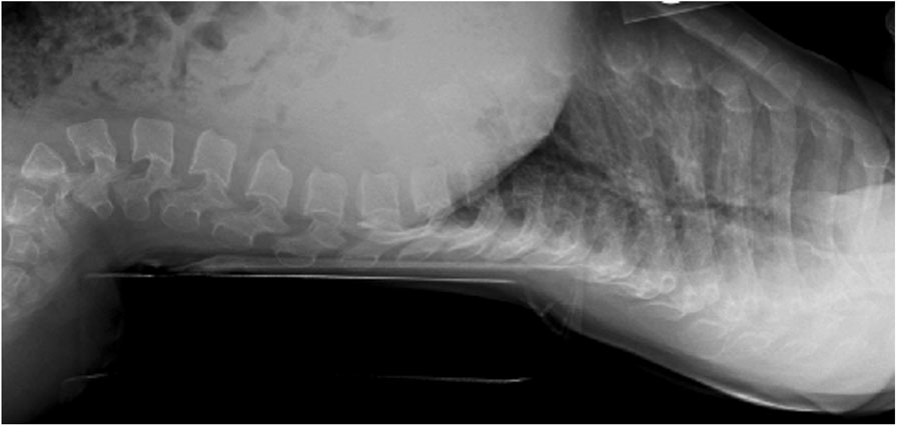
Cross table supine over-a-bolster lateral radiograph shows a mild irreversible kyphotic curve and mild loss of anterior substance of two vertebrae. This view or, alternatively, a cross table prone lateral radiograph, can be used to assess the irreversible component of kyphotic curvature

Wedging or beaking of vertebrae at the apex of the curve with loss of substance of the anterior vertebral body is indicative of the beginning of fixation of the curve [64, 252] (Fig. 36). However, neither development of such beaking, nor progression to a fixed curve is inevitable.

That progression arises from deleterious effects of gravity acting at a disadvantageous angle because of positioning of the infant was first suggested by Beighton and Bathfield [257]. We suggested that a biophysical explanation of progression was likely secondary to a number of nearly uniform features in infants with achondroplasia: hypotonia; macrocephaly; generalized ligamentous laxity. These features mean that when placed in a sitting position, a slumped, C-sitting posture will arise, which can lead to anomalous gravitational forces causing remodeling of an intrinsically abnormal spine [64].

**Fig. 37:**
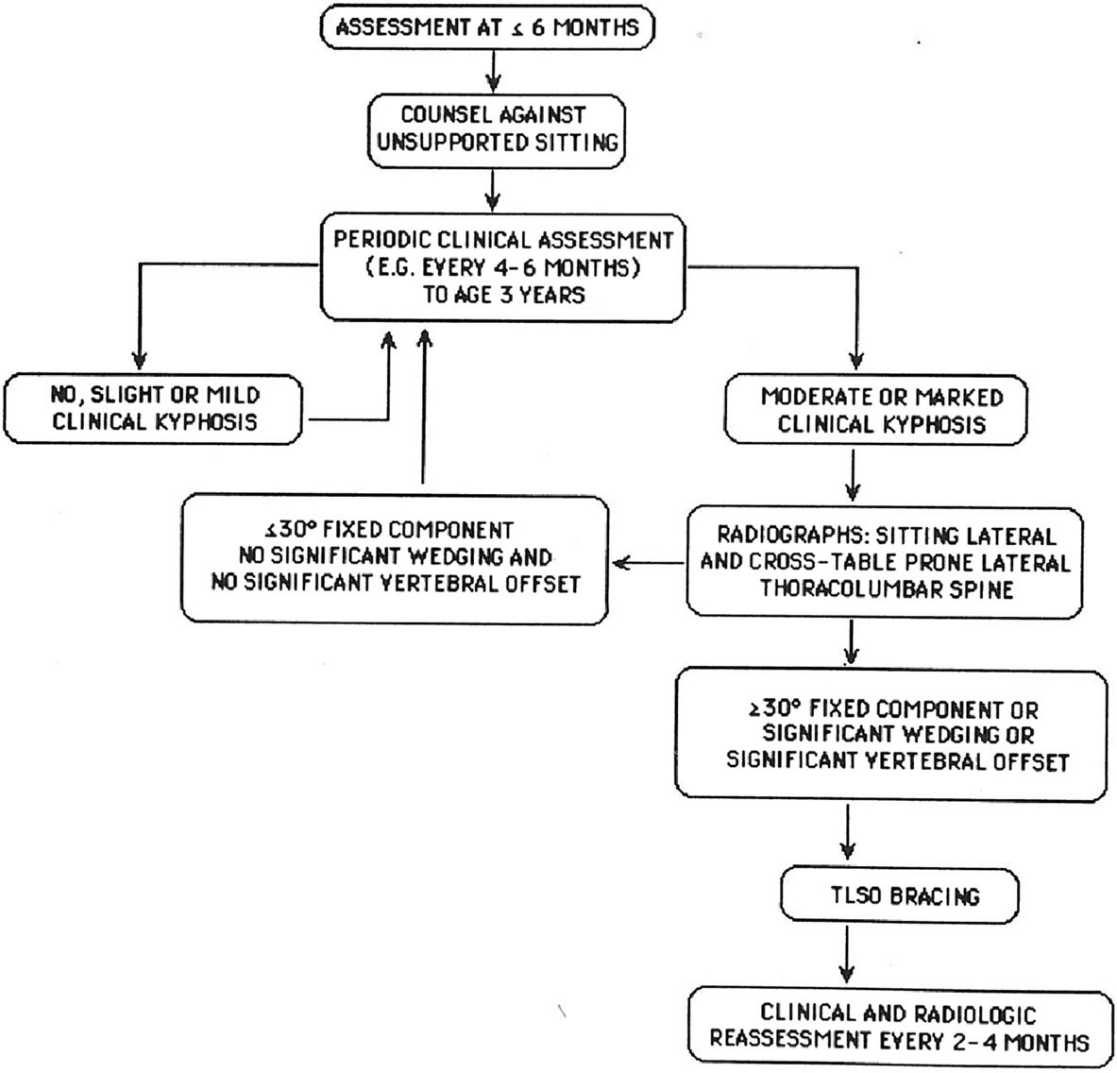
Suggested algorithm for the assessment and management of kyphosis in infants and young children with achondroplasia. Originally published in Pauli RM et al. (1997) Prevention of fixed, angular kyphosis in achondroplasia. J Pediatr Orthop 17:726–733 [64]

If that mechanism is true, then prohibition of unsupported sitting and other strategies to decrease the time spent with gravity exerting disadvantageous force should decrease the likelihood for kyphosis to progress [258]. In the only consecutive, longitudinal study to date, counseling against unsupported sitting in the first 12–15 months of life was effective in markedly lowering the incidence of kyphosis progression [64]. Prohibition of unsupported sitting, good back support in other circumstances, avoidance of umbrella strollers and emphasis on lots of prone positioning (“tummy time”) appear to be generally effective in reducing risk that a kyphosis will progress. (A descriptive guide for parents is available from the author on request or online at the Little People of America website). A protocol has been developed that can act as a guide to the prevention of fixed kyphosis (Fig. 37). Others have more recently confirmed the benefit of such behavioral strategies, as well as demonstrating that those with more severe motor delays (presumably because of more severe hypotonia) are more likely to have persisting kyphotic curves [259]. With such intervention, nonetheless, about 30% of individuals will have a persistent curve [259]. It is for that reason that we developed treatment for those in whom more than a mild, fixed component of the kyphotic curve develops with a modified thoracolumbosacral orthosis (TLSO). (A descriptive guide for physicians and orthotists is also available from the sources cited above). With use of such a protocol very few children should have need for surgical intervention (although, admittedly, compliance with bracing is challenging for some families). In only one of more than 200 children managed in this manner did recurrence of a clinically significant curve arise after following such a protocol (personal observation). Similarly, Xu et al. [260] in a retrospective evaluation showed that bracing, if initiated early enough in life, appeared to be effective in reversing kyphotic curves that otherwise might subsequently require surgery.

In those individuals who were not counseled regarding preventive strategies, or in whom prevention and bracing fails, surgery is appropriate [252, 254, 261]. The aims of surgery are to reduce the severity of the curve, decompress the spine and stabilize it [261]. Spinal arthrodesis with instrumentation seems to be the most effective approach [261], although, of course nothing approaching a controlled study regarding alternative options has been published. Generally surgery is undertaken at around 10–12 years of age [252, 261] in order that late growth not precipitate worsening neurologic status, although some have advocated initial surgical intervention far earlier in life [262].

### Orthopedic concerns: lordosis

**Fig. 38:**
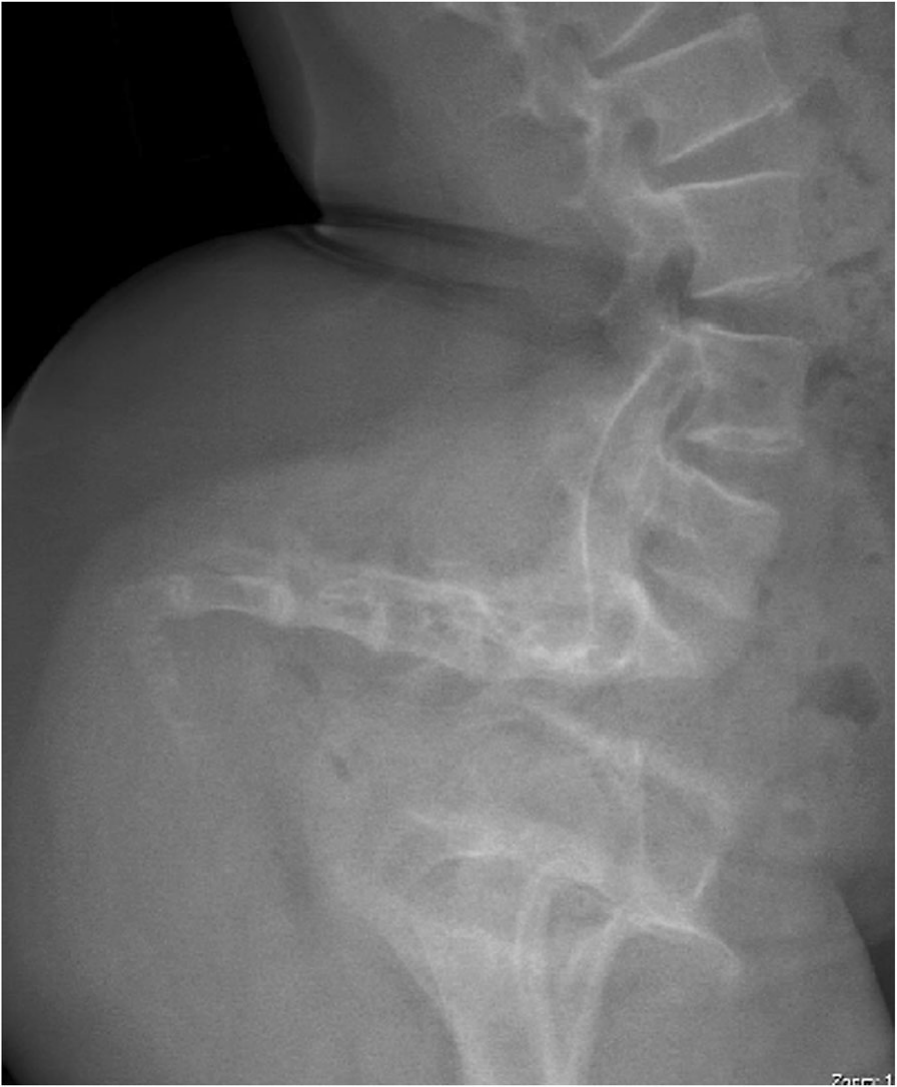
Lateral radiograph of the lumbar and sacral spine. It shows the horizontal sacrum and marked hyperlordosis often seen in those with achondroplasia

Most children develop an exaggerated lumbar lordotic curve (sway back) when they begin to stand and walk (Fig. 38). This hyperlordosis, combined with usual physical characteristics of all 2–3 year olds, often causes parental concern because of the marked abdominal prominence that results. They should be reassured that this is a normal characteristic of children with achondroplasia.

Hyperlordosis is usually asymptomatic and requires no treatment. When marked, there may be an increased incidence of pain at the apex of the curve. When marked, it may also increase the likelihood for intermittent spinal claudication or symptomatic spinal stenosis in adolescents and adults (see below) [263, 264]. Because it may result in a fully horizontal sacrum (Fig. 38), an occasional child may develop pressure-induced, chronic coccydynia (which can be managed with padding of underwear [personal observation]).

Severe hyperlordosis may be diminished in severity by a physical therapeutic exercise program – low back and lower abdominal muscle strengthening and pelvic rotations (personal observation). Because cooperation and compliance are unlikely in young children, generally physical therapy is not appropriate before 5–7 years of age.

### Orthopedic concerns: lumbosacral spine

All individuals with achondroplasia have substantially diminished caliber of the spinal canal along its entire length [265, 266]. A combination of factors contribute to this and to foraminal narrowing. The spine shows decreased interpediculate distances, short and thick pedicles, and many affected individuals have some degree of thoracic kyphosis and lumbar hyperlordosis. The latter two features may make symptomatic spinal stenosis more likely [267].

For the most part, issues arising from the anatomic lumbosacral stenosis are problems of late adolescence and adulthood, with average age of symptoms onset in the 4th decade [256, 268]. The proportion of individuals experiencing symptoms rises from about 20% by age 20 to 80% in the 6th decade [195]. Likely, this is because with age secondary problems, such as arthritis, disk disease, etc. that arise in the general population, similarly arise in those with achondroplasia [263, 264]. Earliest symptoms typically are back pain and buttock pain, with gradual distalward progression of discomfort [195, 269].

It is important to distinguish between intermittent spinal claudication [270] and potentially irreversible symptomatic spinal stenosis, a distinction that is often not made in the surgical literature.

#### Intermittent spinal claudication

Exercise induced intermittent spinal claudication (neurogenic claudication) is a disorder of the elderly in the general population [270]. In contrast, it commonly arises in older children and young adults in those with achondroplasia.

Critical in its differentiation from bony compressive changes in the spine, claudication arises with orthograde activities and resolves (often quite quickly) with rest. Symptoms may include exercise induced tingling, numbness, pins and needles, pain, or a heavy feeling in the legs. Stopping the precipitating activity (standing, walking, running) results in resolution in seconds or minutes. Often persons with achondroplasia find that more rapid relief arises with squatting (personal observation). With careful questioning, a majority of adults report some of these features, but many seek assessment only if walking limitations become marked.

Neurogenic claudication probably arises because of vascular congestion that is increased by blood flow changes associated with exercise and which results in transient nerve root ischemia [271]. As such a mechanism implies, neurogenic claudication results in no permanent damage to the cord or nerve roots. Therefore it generally can be treated non-operatively. No studies have adequately assessed non-operative treatments in the general population [270], and their effectiveness in those with achondroplasia is utterly unexamined. Things seemingly of benefit include weight loss in the overweight and obese, low back physical therapy, and efforts to decrease the severity of hyperlordosis through an exercise program. Because no harm will accrue from physical activity, patients should be encouraged to continue to walk and otherwise exercise. Only if spinal claudication causes marked compromise of physical ability and of quality of life should surgery be considered.

#### Lumbosacral spinal stenosis

A smaller proportion of individuals develop more serious symptoms for which surgical intervention should be more often considered. Bony compression can be of nerve roots or the cord and cauda equina. Signs and symptoms that do not abate with rest suggest that problematic stenosis is developing. Features of particular importance include persistent leg weakness, clumsiness, changes in gait, and development of bladder or bowel incontinence [264]. Clinical examination may demonstrate overt neurologic abnormalities at rest (which are not seen with intermittent spinal claudication), including asymmetries of strength, weakness, abnormal reflexes and/or sensory changes in the legs [264].

Such symptoms and signs should precipitate neuroimaging. However, every person with achondroplasia will have spinal stenosis [266], and that in itself should not be seen as justification for surgical intervention. Furthermore, imaging is remarkably insensitive and nonspecific in assessing spinal stenosis [271]. What imaging can do is identify what levels are most severely anatomically affected, and what additional factors likely precipitated the clinical deterioration. Such documentation is critical when surgery is considered.

Generally, surgical treatment involves extensive and wide posterior laminectomy [254, 264]. Given that virtually all studies regarding operative intervention are retrospective and are subject to ascertainment biases, recall biases, incomplete follow-up and so forth, all conclusions about surgery remain subject to debate and will continue to be so until (and if) prospective, controlled studies are carried out. Tentative conclusions include the following. While lumbosacral spinal stenosis may occasionally require surgery in childhood [267, 268], more often surgery is carried out in the 4th and 5th decades [195, 268]. How quickly after onset of symptoms surgery should be done (versus a trial of non-operative interventions including physical therapy [270]) is controversial [264], with some data supporting aggressive operative intervention soon after onset of symptoms [268]. Improvements following laminectomy do generally arise [264]. As in the general population [272], there is less apparent benefit with time post-surgery, with Pyeritz et al. reporting for example that only ½ of treated individuals show long term benefit [264].

### Orthopedic concerns: knees and lower legs

#### Knee hypermobility

As in the wrists and hips, most children with achondroplasia have excess mobility of the knees. Usually young children show both genu recurvatum (hyperextension beyond 180°) and mediolateral instability. Recurvatum is often between 20° and 70°, apparently secondary to abnormality of form of the tibial plateau [273, 274]. Although this feature is usually insufficient to cause major symptoms or require surgery [273], rarely there is overt tibiofemoral subluxability (personal observation), which is one of the few circumstances in which transient bracing of the knee in a child with achondroplasia may be appropriate. Mediolateral instability is nearly uniformly present in young children with achondroplasia. This often seems to contribute to focal pain precipitated by orthograde physical activity (personal observation). Usually it lessens with age, disappears by adulthood, and rarely, in itself, requires any intervention other than non-specific treatments for pain (rest, warmth, massage and nonsteroidal anti-inflammatories). Lateral instability may be an integral part of the bowing deformity that is often present in those with achondroplasia, both contributing to this problem and responding to its treatment.

#### Leg bowing

Bowing of the legs is a normal feature of average statured children in the first 2 years of life [275]. In average children, early bowing presumably arises secondary to intrauterine positional effects [276], becomes apparent with first walking but then transitions to overcorrected valgus deformity by around 3 or 4 years of age [275, 276].

This is in contrast to what is seen in achondroplasia. In the child with achondroplasia there is often inexorable, continued progression of varus deformity. Between 1/3 and ½ of all children with achondroplasia have substantial bowing at the knees and of the lower legs [273, 277, 278]. Around ¼ will require surgical intervention related to symptomatic bow leg deformity [195, 273]. The severity of bowing is often asymmetric [279, 280]. There is some suggestion that males may be more often affected with clinically relevant bowing than are females [279].

Although referred to as ‘bowing’, in fact the knee and lower leg deformity is not an abnormality within a single, lateral plane. Rather, there is usually lateral, dynamic instability of the knee, varus of the tibia, internal tibial torsion and tibia recurvatum [280]. The complexity of the dynamic deformity is well illustrated using gait analysis [280].

##### Consequences

A major concern in average statured individuals is that substantial varus deformity (of 15° or more) predisposes to knee osteoarthritis [281]. In contrast, arthritis does not seem to be common in adults with achondroplasia [282], although no substantive study has been done to confirm this.

Symptoms that arise most frequently include activity-precipitated pain and self-limitation of walking and other orthograde physical activities [283].

##### Evaluation

Clinical assessment should include asking about activity induced discomfort or pain. Often children will report pain particularly after physically busy days with onset in the afternoon, evening or awakening them from sleep.

**Fig. 39:**
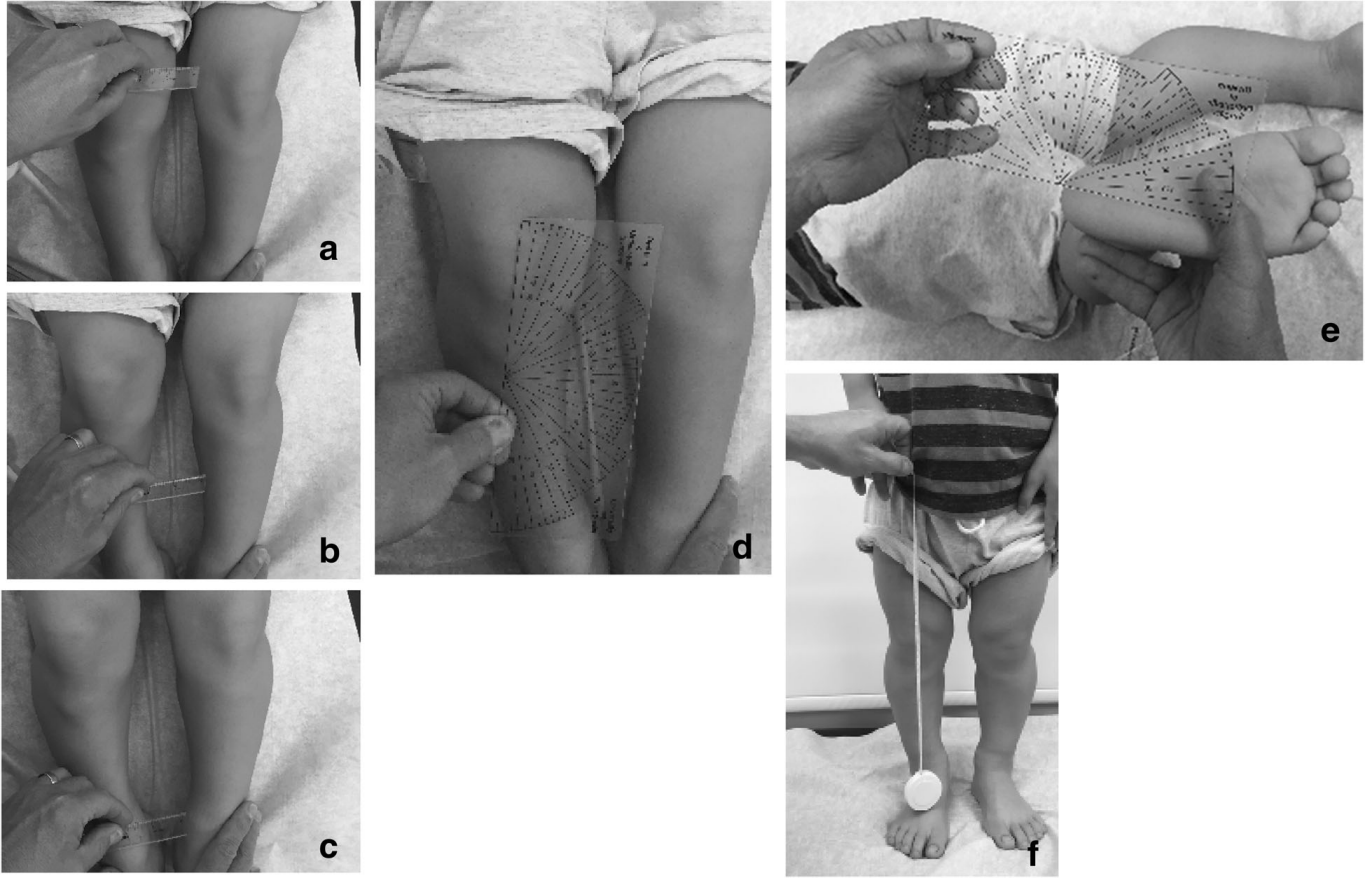
Methods that can be used to monitor progression of varus deformity without repeated radiologic studies (which, however, are needed if the bowing is sufficiently severe that intervention is being considered). **a**–**c** Measurements between the knees, mid-tibiae and medial malleoli with the legs straight and at rest and the feet together; **d** Measurement of maximal varus angle by placing the goniometer at the approximate apex of the tibial bow; **e** Measuring of thigh-foot angle, that is, the angle made by the longitudinal axis of the thigh and the longitudinal axis of the foot when the foot is in its neutral position; this assesses the presence of and severity of internal tibial torsion; **f** Rough assessment of whether the three joints of the leg are in plumb

Examination should assess the following features:

- Severity of genu recurvatum and of mediolateral instability of the knees;
- Measurements of distances between the knees, mid-tibiae and medial malleoli (Fig. 39) (with serial assessment to identify progression and its rapidity through serial clinical measures without the need for frequent radiographs);
- Measurement of thigh-foot angle (Fig. 39) to assess the severity of internal tibial torsion;
- Evaluating whether the weight bearing joints remain ‘in plumb’ in a standing position (Fig. 39), which is helpful in deciding if orthopedic surgical assessment is warranted – in those who are out of plumb, such assessment is indicated [283];
- Evaluation of gait, particularly to ascertain if there is sufficient lateral knee instability to cause a “thrust” – sudden outward movement of the knee with weight bearing.

In addition, in those in whom there is concern about the severity of bowing or who have serious symptoms referable to bowing, radiographic evaluation should be done. Most helpful is a standing, full leg length image with the patellae pointing forward (irrespective of the resultant foot position) [279].

Other assessments that have been used by some include arthrography [284], magnetic resonance imaging of the knee [285], and gait analysis [280].

##### Mechanism of development

There is no consensus about the relative importance of a number of factors that may contribute to the complex varus deformity of achondroplasia. That is unfortunate, since strategies for intervention are at least in part predicated on assumptions about mechanism.

Bowing probably is two discrete processes. In young children the varus deformity is usually primarily proximal – just distal to the knee, while in adolescents varus tends to develop just above the ankle [286]. In young children factors that have been suggested as being important include fibular overgrowth [287], lax lateral collateral ligaments [288, 289], as well as true deformity of the tibia. The relative contribution of each of these and how they interact [289] has yielded conflicting evidence. For example, while Ain et al. [279] found no relationship between fibular overgrowth and severity of bowing, Lee et al. [282] showed just the opposite. Absent clear association, and most certainly absent any evidence that fibular overgrowth is actually a primary cause of leg bowing, there is little to suggest that primary surgery on the fibula is likely to be effective.

##### Treatment

In those in whom any of the following features are present, surgery should be considered: three weight bearing joints out of plumb; persistent lateral knee pain not relieved by conservative measures; development of a lateral thrust [283]. Standard surgery is a valgus producing and derotational osteotomy of the proximal tibia as well as some method to shorten the fibula in order to effectively tighten the lax lateral collateral ligament [286, 289]. Obviously, intervention is tailored to the evaluative findings in a particular child; some may require multi-level osteotomies; in some older children distal tibial osteotomy is appropriate [286]. All such surgery has risk for substantial complications such as infection, malunion, compartment syndrome, peroneal palsy, and need for additional surgery because of recurrence [290].

Alternatives have been suggested in the past but not enthusiastically embraced currently. It was suggested that proximal fibular epiphyseodesis could be a simple surgical way to prevent development of bowing [287]. Of course this assumes that fibular overgrowth is the primary pathogenetic precipitant of bowing. Partial fibulectomy (alone) has also been considered [283]. This would, presumably, both decrease the tension of the fibula and tighten the lateral collateral ligament. There seems to be no literature suggesting that direct operative treatment of the lax lateral collateral ligament is a reasonable option.

Guided growth (or “growth modulation”) using ‘8-plates’ is a means of effecting gradual correction of angular deformity by temporarily limiting growth on one side of a growth plate [291, 292]. Its role in treating varus deformity in achondroplasia is currently unsettled. On the surface, use of 8-plates appears to be illogical since one might expect that they would only correct one dimension of the complex deformity that it usually present. In addition, questions remain whether a ‘sick growth plate’ will respond similarly to that in children of average stature [293]. Certainly, given the slow rate of growth in bone dysplasias, correction will be far slower and symptoms will not be relieved for the time needed for correction. Nonetheless, impressive outcomes have been demonstrated (P. Stevens, personal communication 2015). Limited published data regarding its use in those with achondroplasia [294] suggest that at least partial correction of malalignment can be accomplished. There even is anecdotal evidence that correction in one plane (varus deformity) will secondarily results in improvement of the other components (internal torsion and lateral instability) (P. Stevens, personal communication 2015). Whether this (surgically far simpler and far less debilitating) option is a good alternative remains uncertain.

##### Unanswered questions

Treatment of bowlegs in achondroplasia is a good example of the many unknowns that remain. For example, questions that have not been adequately addressed to date include:

- What is the pathogenetic mechanism giving rise to varus deformity?
- How much genu varum is tolerable in those with achondroplasia?
- What is the prevalence of knee osteoarthritis in adults with achondroplasia, and does it correlate with the severity of varus deformity? Recent animal studies suggest that activation of FGFR3 may confer protection against development of osteoarthritis [295, 296]. If this is true in humans as well, then those with achondroplasia may tolerate more severe bowing without debilitating secondary arthritis than is the case in the general population.
- What are rational indicators for surgery?
- At what age is surgery best undertaken?
- What should be the role of 8-plates in treating bowing?
- Etc.

### Other features of the knees in those with achondroplasia

The availability of imaging techniques – particularly magnetic resonance imaging and direct visualization by arthroscopy – has resulted in recognition of a number of anatomic variants of the knee in achondroplasia [285, 297]. Of these, the finding likely of greatest significance is an exceedingly high prevalence of discoid lateral menisci.

Although relatively common in the general population [298], there seems to be a much higher prevalence in children and adolescents with achondroplasia who present with knee pain. There are a host of causes for knee pain in this population, the most common of which is varus deformity. A discoid lateral meniscus, which may predispose to meniscal injury [298, 299], should be considered particularly in those with lateral joint line pain and tenderness [285, 299, 300]. Unfortunately, to date the data related to discoid lateral meniscus suffer from the usual problems – small series, ascertainment biases, uncontrolled interventions, etc. However, that treatment of torn discoid lateral menisci does seem to result in symptom resolution suggests that this may, in fact, be another cause of knee pain in children and adolescents with achondroplasia.

### Orthopedic concerns: shoulders, elbows and wrists

#### Shoulders

Shoulder hypermobility is virtually constant in individuals with achondroplasia [301] (and personal observation). This evidently arises because of unusual shape of the humeral head so that anteroinferior subluxability out of the glenoid fossa may occur, but, while subluxability is common, pain because of this instability is rare [273]. Nonetheless it is probably prudent to limit the frequency of induced subluxation. Two activities (and likely others with similar dynamics) seem to particularly precipitate subluxation – butterfly stroke in swimming and dead-lifting of weights (personal observations). Therefore, in those with clinically demonstrably unstable shoulders (anteroinferior subluxability, apprehension sign) those activities should be avoided. Increasing subluxability has also been noted during humeral lengthening [301].

#### Elbows

In contrast to nearly all other joints, the elbows are stiff in those with achondroplasia. Most individuals develop limitation of elbow extension beginning in childhood [273]. Usually this is moderate, ranging from around 20° to 60° of decreased extension [273]. This results in effective shortening of the arms even more, and limits reach accordingly. In the minority in whom radial head dislocation arises, even greater functional consequences will result, since not only will reach be diminished, but pronation and supination will also be limited (although this can in part be compensated for because of excess mobility at the wrists). Nevertheless, surgical treatment is almost never appropriate, unless humeral lengthening is elected because of reaching problems. Such reaching problems often can be addressed by using various adaptive devices (see section Adaptive needs), Nonetheless, humeral lengthening (as part of general extended limb lengthening or alone to treat issues related to reach limits and adaptive needs) has been carried out in a large number of individuals with achondroplasia [302]. This may be necessary in order to assure independence for perineal hygiene, particularly in those who have limitation of trunk mobility (e.g. because of surgical spine fusion). About 6–12 cm of lengthening (2–5 in.) of lengthening can be expected [302–304]. Although modest, this may be sufficient to allow for greater independence for personal hygiene.

#### Wrists

The wrists in almost all children and in some adults are hypermobile [278] (Fig. 26). Some of those with hypermobility demonstrate marked dorsoventral instability (personal observation), which seems to correlate with functional difficulties related to wrist stabilization. Excess wrist movement and the need to stabilize the wrist for fine motor tasks can cause a number of problems. Some children complain of fatigability after very short periods of writing; others have difficulty generating sufficient pressure to even make marks with a pencil. A number of options have proven to be beneficial (see below under Adaptive needs). Surgery is never appropriate.

### Dental concerns

**Fig. 40:**
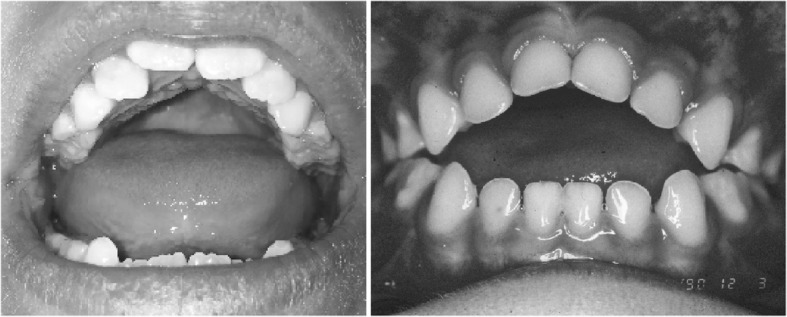
Two of the common occlusional abnormalities seen in children with achondroplasia. On the left note the narrow, V-shaped anterior palate and the resulting “palisading” of the upper incisors. The right photograph shows a severe anterior open bite

Because of hypoplasia of midfacial structures [305] malocclusion is common. In addition to maxillary hypoplasia, there is relative overgrowth of the mandible; it is uncertain whether mandibular growth is itself normal [305] or diminished [306] but less so than is the diminishment of maxillary growth. Given these features, one might expect an extensive literature concerning management of malocclusions in those with achondroplasia; it is surprising how little literature concerning this is available [307–312]. Most children with achondroplasia will benefit from orthodontic care. Primary problems that are most often seen include marked narrowing of the anterior palate, with palisading of the upper incisors (Fig. 40), open bite (Fig. 40) and, particularly in older children and adolescents, underjet related to the disproportionate (if normal) growth of the mandible (personal observations).

Many children appear to benefit from preparatory orthodontia. Palatal expansion [309] is often used to increase the width of the maxilla and to improve the effects of subsequent traditional orthodontia.

### Cardiovascular concerns

Mortality studies not only have shown an increased risk for death in infancy, but also greater rates of death across all ages [107, 108]. Particularly noteworthy is evidence of substantially greater risk of death secondary to cardiovascular conditions. Indeed heart disease related deaths were reported as ten times expected in young adults and, overall, twice the anticipated rate compared with average statured individuals [108].

A number of factors could cause these differences. It could be that there is a primary effect of FGFR3 on cardiovascular function. More likely it may be related to other risk factors that occur at higher frequency in adults with achondroplasia. These could include decreased physical activity (because of the physical challenges inherent in achondroplasia), increased obesity [172] or higher than typical incidence of lifestyle related risks.

Unrecognized hypertension is probably particularly important. Well over ½ of adults with achondroplasia are pre-hypertensive or hypertensive (J. Hoover-Fong, personal communication 2014). That this is underappreciated particularly arises because of the difficulty in obtaining accurate measurements because of the large circumference but short length of the upper arm. Therefore, many individuals with achondroplasia do not have routine blood pressure assessment and almost certainly are undertreated. Specific blood pressure cuffs (e.g. GE Small Adult Long Soft-Cuf), and use of the forearm when needed (J. Hoover-Fong, personal communication, 2014) should make better monitoring of this issue possible.

### Low frequency but non-coincidental processes

#### Acanthosis nigricans

Acanthosis nigricans is an infrequent but not rare consequence of achondroplasia [313–316]. It typically is demonstrable as thickened, velvety, excessively pigmented skin of the neck and, less frequently, of the axillae and inguinal region [317]. It most often arises in late childhood or adolescence [315, 317]. In a retrospective assessment of nearly 500 consecutive individuals, acanthosis nigricans was diagnosed in about 10% of those with achondroplasia [317]. Parents and care providers should be reassured, first, that this is not dirt and cannot be scrubbed away; secondly, that it typically remains clinically mild; and third, that it is a harmless accompaniment of achondroplasia. The latter is important since, in other circumstances acanthosis nigricans is a marker for insulin resistance and diabetes [318]. In this setting it is not [315].

Acanthosis nigricans appears to arise as a direct result of constitutive activation of *FGFR3* [319]. It is present in varying severity and varying frequency in many other *FGFR3* disorders, including hypochondroplasia [315, 320–322], thanatophoric dysplasia [86, 87], SADDAN syndrome [93], Crouzon syndrome with acanthosis nigricans [97, 323], and in isolation with little or no skeletal abnormality [99–101]. Note that in these last, descriptions there is inadequate radiologic and clinical detail to clearly rule out a diagnosis of hypochondroplasia.

#### Craniosynostosis

Craniosynostosis is the primary phenotypic consequence of certain *FGFR3* mutations, particularly in Muenke syndrome [98]. It is also a prominent feature of Crouzon syndrome with acanthosis nigricans [97] and thanatophoric dysplasias [324]. In these, and in achondroplasia, *FGFR3* mutations cause abnormalities of ossification in membranous bone including premature sutural fusion [325]. It is not especially surprising, then, that craniosynostosis infrequently accompanies achondroplasia [186, 326–330] (and personal observations).

In achondroplasia, when craniosynostosis is present it does not seem to be suture specific, with involvement of the metopic [326–328], coronal [326, 328, 330], sagittal [329], (and personal observation), lambdoidal [326, 328], (and personal observation), frontosphenoidal [327], (and personal observation) and squamosal [328, 330] sutures, etc. in varying combination.

In a retrospective review, 4 of 477 (0.8%) consecutively assessed individuals with achondroplasia had confirmed craniosynostosis (unpublished personal observations). So, while uncommon, nevertheless should any type of calvarial asymmetry or unusual head shape be apparent, particularly if it worsens over time, assessment for craniosynostosis should be undertaken. If needed, surgery may be complicated by the presence of prominent emissary veins [330] that arise, as described above, secondary to jugular venous outflow obstruction.

### Adaptive needs

Because of the biophysical differences present in both children and adults with achondroplasia, many adaptive needs may be present at all ages. At least in North America, most families and affected individuals have embraced the idea that the environment should be modified for the individual rather than requiring the individual to be ‘modified’ to fit a standard environment. Given the number of potential adaptations and the varied requirements of different ages, a complete description of all of these is beyond the purview of this review. Many alternatives can be found through the Little People of America (https://www.lpaonline.org). Likewise, general governmental mandates regarding accessible environments and accomodations for differences won’t be discussed. Four examples of adaptations needed by some individuals with achondroplasia are given below.

In young school-age children, the combination of brachydactyly and hand and wrist joint hypermobility often make certain fine motor tasks difficult. In particular, many children find it difficult to print with a standard pencil – making marks barely perceptible, or being unable to stably hold the pencil. Alternatives that can be tried to solve that difficulty can include use of free-flowing pens or markers instead of pencils, providing ‘fatty’ grips for the child’s pencils, using a small wrist stabilizing brace when fine motor tasks are being undertaken, or early transitioning to keyboarding. Involvement of an occupational therapist will often be beneficial.

**Fig. 41:**
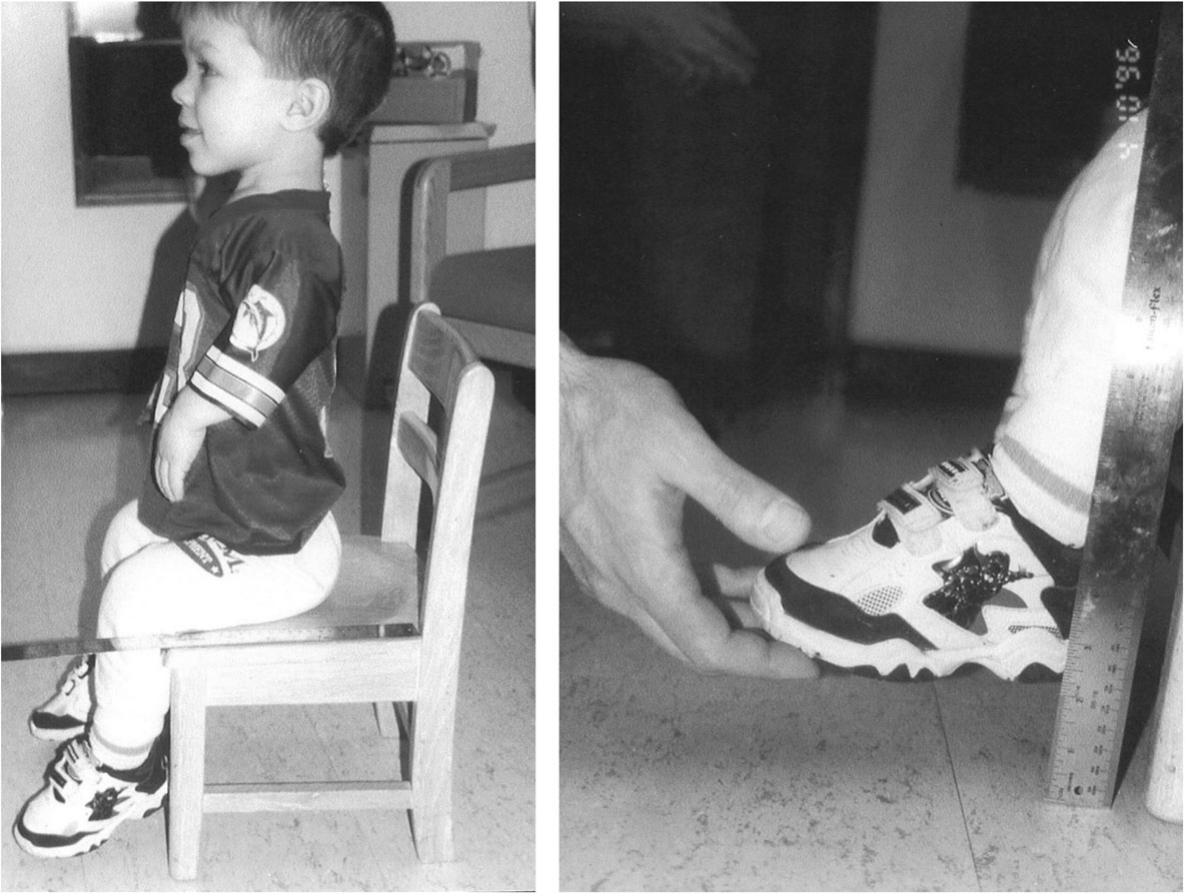
On the left – Demonstration of the difficulty a typical child with achondroplasia has when sitting in a school chair. Note that there is neither back nor foot support. Method of measuring for back support is also shown. On the right – measurement that can be made to guide modification to allow foot support

**Fig. 42:**
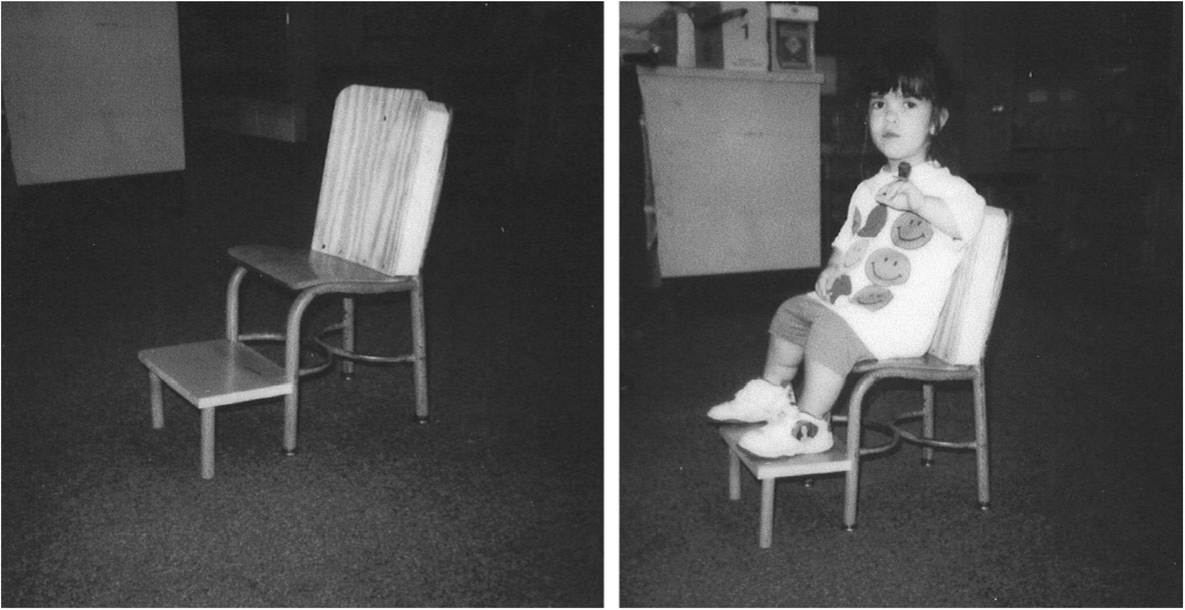
Left – Example of modifications made in a school chair to allow for back and foot support. Right – Resultant supported, comfortable sitting for a child with achondroplasia

Comfortable sitting for young children is also a challenge. Because of rhizomelic shortening of the legs, use of a standard, unmodified chair results in both an unsupported back and dangling legs. Often this then results in chronic back discomfort, leg numbness, or both. While various special chairs are available, children usually much prefer simple modifications of a typical chair for their classroom, since these will far less identify them as ‘special’, ‘disabled’, etc. These modifications can be made to wood or plastic chairs (Figs. 41 and 42).

**Fig. 43:**
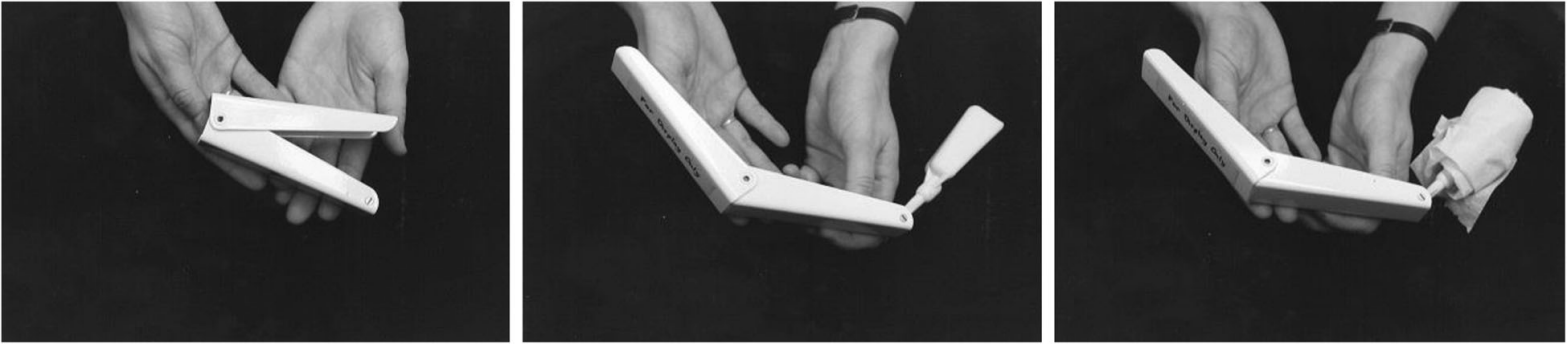
Example of a foldable ‘bottom-wiper’ that can be carried in a pocket or purse and used for personal hygiene by wrapping toilet paper around its end, and swishing it in the toilet to dispose of the paper after its use

Personal perineal hygiene is often an issue as well. In children, bottom wiping can be accomplished by a simple strategy – rather than wiping from the side, the child can be taught to hop off of the toilet, and bend far forward, reach between the legs and wipe (front to back, particularly in girls) in that manner. This precludes the reaching difficulty resulting from arm shortening and takes advantage of the truncal hypermobility that is prominent in most children with achondroplasia. In older individuals and in any who have limited trunk mobility (e.g. from prior spinal fusion), use of a ‘bottom wiper’ is an option (Fig. 43). Finally in a small number, in whom perineal hygiene just cannot be accomplished otherwise, humeral lengthening (see above) can be considered.

As a final example – driving. Persons with achondroplasia generally do not need major modifications of an automobile such as hand controls. Two issues do need to be addressed, however. First, leg shortness usually requires the use of pedal extenders, which are available from a number of sources and which can be straightforwardly applied and removed when an automobile is shared with an average stature driver. Secondly, arm shortening results in most individuals with achondroplasia sitting very near the steering wheel. This is an issue related to air bag deployment from the steering wheel. For anyone in whom measurement from the middle of the steering wheel to chin or chest is 12 in. (~ 30 cm) or less, the driver side air bag should be disabled, since its deployment can result in life threatening injury in those circumstances [1, 331].

### Quality of life

The impact of a medical condition such as achondroplasia can be thought of in a number of ways. In a broad sense, “quality of life” reflects all consequences of a disorder that are not narrowly medical. It can be divided between physical consequences that impact life’s quality and the psychosocial effects of the disorder. Unfortunately many quality of life studies have combined various skeletal dysplasias, and in only some can the specific effects of achondroplasia be parsed from the presented data [332–334]. Very few studies that can be considered assessments, in one way or another, of quality of life have been published specifically regarding achondroplasia [335–339].

In the medical domain, certain findings of quality of life studies stand out. First, pain is commonly present in adults with achondroplasia. Indeed, nearly 2/3 of adults report chronic pain, a figure dramatically higher than in the general population [340]. Secondly, pain is associated with compromised physical function; that includes limited walking endurance and loss of independence for activities of daily living [340]. In fact, 10–15% of adults were dependent on others for activities of daily living such as bathing and toileting [340]. Third, overall functional health status is impaired in adults with achondroplasia (but only surprisingly mildly so), with much of that apparent decline arising in the 4th and 5th decades of life, and much of that related to lumbosacral spine issues [341]. Gollust et al. also showed that quality of life in the health domain is diminished [342].

Psychosocial consequences of small stature have been described for a long time. That shortness of any cause can result in social problems, decreased self-esteem and so forth has been proclaimed by many, but remains a controversial issue [342]. For chondrodysplasias collectively, Hunter showed that there is both apparent increases in depression and diminished self-esteem [332, 333]. Gollust et al. documented that in those with achondroplasia, there is diminished income, less education and less successful employment, and that all non-health domains of quality of life – social/economic, psychosocial/spiritual, family – were negatively affected [342]. Achondroplasia, indeed, “constrains life”, despite obvious resilience in most of those affected [342].

Nishimura & Hanaki assessed psychosocial adaptation and coping in *children* with achondroplasia [343]. Remarkably, they demonstrated unimpaired self-concept and effective coping strategies despite encountering negative experiences related to their small stature. Similarly, Rohenkohl et al. found that overall quality of life in children and young adults with achondroplasa was nearly identical to that of normal controls [336]. The contrast with other, primarily adult-based studies is striking. Does self-image and self-esteem change with age? Or does this reflect a more positive attitude about dwarfing conditions directed toward this younger cohort? (Note, however, that Rohenkohl et al. [336] and Witt et al. [338] seem to have demonstrated improvement of quality of life with increasing age up to adulthood, a finding apparently incompatible with other results summarized).

A diagnosis specific instrument for assessment of quality of life in achondroplasia has been developed [337, 339]. Called the “APLES” (Achondroplasia Personal Life Experience Scale) this could be of utility in the assessment of individuals (older children and young adults) with achondroplasia compared with their diagnostically identical peers.

### Pregnancy

Other than anecdotes, case reports and small series [344, 345], there is very little in the literature regarding risks and management of pregnancy in women who have achondroplasia. A questionnaire survey of a convenience sample of 87 women [346] allows some tentative conclusions to be drawn. Most women with achondroplasia have near-normal trunk size, which is likely the reason that most can carry pregnancies to term. Pregnancy-related complications are uncommon; the most serious of these seem to be worsening spinal claudication symptoms and, rarely, respiratory failure [344] (and personal observations). Risk for cardiorespiratory problems is probably increased in those who are of very small stature and have shorter than typical trunks, in those with severe spinal deformity, and in those who have had apnea-associated complications in their past.

The possibility of respiratory problems should be addressed early in every pregnancy of women with achondroplasia. It is prudent to complete baseline pulmonary function testing early in pregnancy and to involve a pulmonologist so that, should respiratory issues arise, an expert with antecedent knowledge of the patient will be at hand. Rarely interruption of a pregnancy by termination (personal observation) or delivery prematurely [347] (and personal observations) may be necessary as a maternal life-saving measure.

All women with achondroplasia must be delivered by elective Cesarean section because of uniform narrowing of the pelvis and cephalopelvic disproportion [345]. (Parenthetically, one wonders what happened in centuries past if a woman with achondroplasia became pregnant.) This should be a planned Cesarean delivery without a trial of labor. Anesthesia for this procedure remains both controversial and challenging. No consensus has arisen whether or when general, spinal or epidural anesthesia is preferable [347] and there is virtually no data upon which to base such a decision. Dubiel et al. [347] outline the anesthetic challenges related to the statural, airway, spinal, respiratory and neurologic features of achondroplasia that can make decision making regarding anesthetic care so challenging.

### Prenatal diagnosis

It has long been recognized that anatomic prenatal diagnosis of achondroplasia is difficult and usually not feasible prior to mid-pregnancy whether by radiography [348] or ultrasonography [349]. The challenge of using ultrasound resulted in frequent missed diagnoses and misdiagnoses [350]. While considerable advances have been made in technique and interpretation [351, 352], recognition of achondroplasia, particularly in sporadic cases, remains difficult.

Now, of course, prenatal diagnosis is accomplished principally through molecular testing. One circumstance in which it may be utilized is when both parents are affected by achondroplasia. They then have a 25% risk that any conceptus will be homozygous and likely to either die in utero or neonatally. The community of little people has, for the most part, embraced prenatal testing for this purpose, while rejecting consideration of termination if results show either heterozygous achondroplasia or average stature [353]. The second scenario is when ultrasonographic investigations, either routine or for cause, suggest the presence of a short limb dwarfing disorder. In this circumstance it is critical to determine if achondroplasia (the most common of the short limb conditions) is or is not causal. This can be done either through direct analysis of *FGFR3*, as part of a skeletal dysplasia molecular panel, or using whole exome sequencing.

Recently non-invasive, cell free methods using maternal plasma have been developed, including specifically for achondroplasia [354–358].

### Possible future therapies

As understanding of the molecular processes underlying achondroplasia have become better understood, a number of suggestions for possible molecular pharmacologic therapy have emerged [359]. Whether any of these ideas will eventually lead to effective therapy of achondroplasia is, of course, unknown.

The most advanced of these possible therapeutic options is investigation of a C-type natriuretic peptide (CNP) analog. CNP in many ways acts as a counterbalance to the effects of FGFR3 on the growth plate. Loss of function mutations of the CNP receptor (the transmembrane natriuretic peptide receptor – NPR-B) results in another, severe dwarfing disorder called acromesomelic dysplasia, type Maroteaux [360]. This disorder is certainly not a mirror image of achondroplasia – sharing almost nothing phenotypically with it except for marked small stature. Nonetheless it was postulated that increasing CNP activity could counteract the excess negative signal of *FGFR3* mutations. And, indeed that appears to be true [361]. An analog was developed by BioMarin Pharmaceuticals that has greater stability, and animal experiments were begun using this peptide, called BMN-111 [362]. BMN-11 l (now called Vosoritide by BioMarin) reversed the achondroplasia phenotype in the mouse model of this disorder [363] and appeared to enhance bone growth in normal cynomolgus monkeys, including increases in growth plate thickness and increase in lumbar vertebral foraminal size [362]. CNP also seems to have beneficial effect on midfacial development [364]. Human trials have begun (https://www.biomarin.com/products/pipeline/bmn-111/). In fact, currently these trials include an expanded phase 2 trial in infants and young children as well as active recruitment for a phase 3 trial (https://clinicaltrials.gov). Whether this will be effective long term, whether undesired consequences of counteracting FGFR3 function will arise, and how early in life it would need to be administered to have salutary effects on health are all still unknown. Likewise, the demonstration of elevation of CNP at baseline in those with achondroplasia suggest that there might be tissue resistance to CNP (and its analogs) that could complicate this therapy [365].

Many other suggested therapies are at various stages of investigation. Unal & Tugan [366] have suggested use of mesenchymal stem cells as a source for native CNP. Yamashita et al. [367] showed that statin treatment ‘rescued’ stem cells (correction of degraded cartilage formation) and a mouse model of achondroplasia (with significant recovery of bone growth). If such therapy was seriously to be considered, the long term safety of chronic administration of statins to children would need to be demonstrated [368]. Recent studies raise questions about the mechanism whereby statins might have consequence on achondroplastic bone [369]. Meclizine (an over the counter medication for motion sickness) improves long bone growth in a mouse model of achondroplasia, apparently through inhibition of activity of downstream effectors of FGFR3 [370–372]. Apparently a phase I clinical trial of meclizine will commence shortly (http://www.growingstronger.org/).

Additional experimental protocols are investigating:

- Inhibition of chaperone proteins of FGFR3, which inhibition can result in ubiquitination and degradation of FGFR3 and, in turn, decreased signaling [373];
- Use of a small molecular inhibitor (tubacin) of histone deacetylase 6 which results in reduced accumulation of FGFR3 at the growth plate [374];
- Use of decoy, soluble FGFR3 to decrease binding of FGFs and consequently decreased signaling [375], which approach apparently has entered early clinical trials (http://www.growingstronger.org/);
- Direct blocking of the FGFR3 binding domain [376];
- Reducing the activity of FGFR3 using a tyrosine kinase inhibitor [377].

Is the future now? Many individuals within the small stature community would embrace treatments that will eliminate some of the medical consequences of achondroplasia summarized here, while at the same time showing little or no enthusiasm for treatments exclusively or primarily aimed at linear growth enhancement.

## Conclusions

A great deal has been learned about the consequences of achondroplasia on those who are affected. Nonetheless, the quality of care that can be provided is compromised by the limited quality of the evidence that is, for the most part, available. Although the future may include pathway driven therapies, there will remain a need for quality clinical investigations regarding the natural history and optimal interventions for the sequelae of achondroplasia.

## Abbreviations

APLES: Achondroplasia personal life experiences scale
bipap: Bilevel positive airway pressure
BMI: Body mass index
cm: Centimeter
CNP: C-type Natriuretic Peptide
cpap: Continuous positive airway pressure
CT: Computerized tomography
FGF: Fibroblast Growth Factor
*FGFR3*: Fibroblast Growth Factor Receptor 3 gene
FGFR3: Fibroblast Growth Factor Receptor 3 protein
FM: Foramen magnum
MRI: Magnetic resonance imaging
NPR-B: Natriuretic Peptide Receptor B
RAMP: Recurrent-autosomal dominant-male biased-paternal age effect
S.D.: Standard deviation
SADDAN: Severe achondroplasia – developmental delay – acanthosis nigricans
TLSO: Thoraco-lumbo-sacral orthosis
